# The anti-*Candida haemulonii* activity and bioactive metabolites of *Streptomyces anandii* NC-SA6

**DOI:** 10.3389/fcimb.2026.1851966

**Published:** 2026-06-30

**Authors:** Chunxi Yang, Chaoyu Cui, Yanru Chen, Fengyi Deng, Zimei Peng

**Affiliations:** 1 Institute of Clinical Medicine, Jiangxi Provincial People’s Hospital, The First Affiliated Hospital of Nanchang Medical College, Nanchang, China; 2 Jiangxi Key Laboratory for Excavation and Utilization of Agricultural Microorganisms, Jiangxi Agricultural University, Nanchang, China

**Keywords:** *Streptomyces anandii*, antifungal activity, metabolomics, genomics, *Candida haemulonii*

## Abstract

**Introduction:**

The rapid advancement of multi-omics strategies has profoundly facilitated the in-depth exploration of microbial physiological characteristics, accelerated the discovery of novel bioactive secondary metabolites, and promoted the mechanistic elucidation of their biological functions. As a dominant genus within the phylum Actinomycetota, *Streptomyces* is widely recognized for its remarkable capacity to synthesize a diverse spectrum of clinically applicable antibiotics. *Candida haemulonii*, an emerging opportunistic fungal pathogen, has emerged as a typical multidrug-resistant species closely associated with outbreaks of nosocomial infections, posing a severe threat to clinical antifungal therapy.

**Methods:**

In this study, a novel strain designated as *Streptomyces anandii* NC-SA6 was isolated and systematically identified *via* gradient dilution method, multilocus sequence analysis (MLSA), coupled with comprehensive physiological and biochemical characterization assays. The optimal fermentation condition was optimized by controling a single variable method and measuring the diameter of the inhibition zone. The antimicrobial spectrum was tested against a panel of pathogenic strains, and the MIC value was measured by using broth microdilution. Finally, antifungal compounds were analyzed by combnining genome and metabolomic.

**Results:**

We identified a strain with a spectrum antimicrobial activity against human pathogenic *Candida* species and Gram-positive bacteria. The optimal fermentation conditions are 4-day fermentation broth in No. 6 medium, and the MIC values of S. anandii NC-SA6 fermentation broth for Candida auris BJCA001 and Candida haemulonii 190070, the MIC50 values were 36.8 mg/ml and 18.4 mg/ml, respectively. Whole-genome sequencing analysis revealed the presence of 20 biosynthetic gene clusters (BGCs) responsible for secondary metabolite biosynthesis. Untargeted metabolomic analysis identified a total of 1703 metabolites. Functional annotation demonstrated that 43.5% of these metabolites were characterized bioactive compounds, including antimicrobial agents, antifungal agents, antitumor inhibitors, and other pharmaceutical molecules; the remaining 56.5% were uncharacterized metabolites, indicating the existence of potential secondary metabolites.

**Discussion:**

Collectively, this integrated multi-omics study identified *S. anandii* NC-SA6 as a promising microbial resource for mining antifungal metabolites targeting multidrug-resistant *C. haemulonii*, highlighting its tremendous potential for the discovery and developmental research of novel antifungal agents.

## Introduction

Invasive candidiasis, a severe systemic fungal infection caused by pathogenic *Candida* species, remains a prevalent and clinically challenging infectious disease with high morbidity and mortality. While *C. albicans* remains the primary etiological pathogen, accumulating clinical evidence has revealed a steadily increasing incidence of infections caused by non-albicans *Candida* (NAC) species, accompanied by the continuous emergence and spread of novel opportunistic fungal pathogens (Lass-Flörl et al., 2024; McCarty et al., 2021). First identified as a human pathogen in 1984 (Francisco et al., 2023), *C. haemulonii* has become a representative emerging rare fungal pathogen. Its clinical isolation rate has exhibited an upward trend in recent years, with mounting studies confirming its multidrug-resistant phenotype and potential for nosocomial cross-transmission and outbreak infections (Giusiano et al., 2006; Xiao et al., 2020). Consequently, the development of novel, efficient antifungal therapeutic agents targeting invasive candidiasis has become an urgent clinical demand, which is critical to overcoming fungal multidrug resistance and controlling the prevalence of invasive fungal infections.

Secondary metabolites, also referred to as natural products, are mainly biosynthesized by microorganisms, plants, and some marine organisms, covering a variety of clinically vital drugs including classic antibiotics, antitumor agents, cholesterol-lowering statins, and immunosuppressive drugs such as cyclosporine (Abdel-Razek et al., 2020; Katz and Baltz, 2016; Pacios-Michelena et al., 2021). The genus *Streptomyces* is highly valued in natural product research for its unparalleled ability to produce structurally diverse and biologically potent secondary metabolites, including antibiotics, siderophores, and various bioactive small molecules (Alam et al., 2022; Butt et al., 2023; Ribeiro and Van Der Sand, 2024; Sivalingam et al., 2024). Notably, *Streptomyces* species account for the biosynthesis of the majority of naturally occurring antibiotics, most of which are derived from soil-dwelling strains. Owing to their versatile biological activities, *Streptomyces* strains have been widely applied in aquaculture, agricultural production, and clinical medicine (Alam et al., 2022; Bhat and Nayaka, 2023; Bubici et al., 2019; Butt et al., 2023; Kemung et al., 2018; Wang et al., 2024; Zainuddin et al., 2023).

The discovery of novel microbial bioactive compounds from *Streptomyces* has evolved from traditional activity-guided fractionation and purification strategies to advanced integrated approaches, combining long-read genome sequencing and in silico genome mining tools such as antiSMASH, which enables efficient identification and prediction of BGCs mediating specialized metabolite biosynthesis (Blin et al., 2021). Nevertheless, a large number of BGCs in sequenced *Streptomyces* genomes remain transcriptionally silent or cryptic under conventional culture conditions, representing an enormous untapped reservoir of potential bioactive small molecules (Jensen, 2016; Rigali et al., 2018). Therefore, the integration of whole-genome mining with untargeted metabolomic profiling based on liquid chromatography-tandem mass spectrometry (LC-MS/MS) can achieve accurate and comprehensive identification of both known and novel bioactive metabolite derivatives, bridging the gap between genotype and phenotype.

In this study, we aimed to screen and identify *Candida*-antagonistic microbial strains *via* phylogenetic and phylogenomic analyses, and evaluate their potential to produce antifungal metabolites against multidrug-resistant *C. haemulonii*. Genome mining was performed to predict and characterize secondary metabolite BGCs in the target strain; subsequently, untargeted LC-MS/MS-based molecular networking was employed to systematically profile and identify the secondary metabolites in fermentation products. This study provides a scientific basis for the development of novel antifungal agents from environmental *Streptomyces* resources.

## Materials and methods

### Strains and culture conditions

All bacterial and fungal strains used in this study are listed in **Table 1**. Bacterial strains were cultured in Luria-Bertani (LB) broth (10 g tryptone, 5 g yeast extract, and 10 g NaCl per liter, pH 7.0). Fungal strains were maintained on Yeast Extract Peptone Dextrose (YPD) medium (10 g yeast extract, 20 g peptone, 20 g dextrose, and 20 g agar per liter). All tested strains were preserved in the laboratory strain collection.

**Table 1:** The list of the strains in this study.

Strain	Cultivation temperature	Reference or Source
Streptomyces anandii NC-SA6	28 °C	This study
Candida krusei ATCC6258	28 °C	Laboratory collection
Candida haemulonii 190070	28 °C	Laboratory collection
Candida auris BJCA001	28 °C	Laboratory collection
Candida albicans SC5314	28 °C	Laboratory collection
Escherichia coli ATCC25926	37 °C	Laboratory collection
Shigella flexneri ATCC12022	37 °C	Laboratory collection
Pseudomonas aeruginosa PA14	37 °C	Laboratory collection
Burkholderia cenocepacia H111	37 °C	Laboratory collection
Bacillus velezensis IF-1	28 °C	Laboratory collection
Bacillus cereus ATCC14579	30 °C	Laboratory collection
Staphylococcus aureus ATCC29213	37 °C	Laboratory collection

### Isolation, screening, and identification of antagonistic *Streptomycete* against *Candida haemulonii*

*Streptomyces anandii* NC-SA6 was isolated from soil samples collected in Nanchang, Jiangxi Province, China, using a selective isolation medium supplemented with 50 ppm potassium dichromate (K_2_Cr_2_O_7_). Because K_2_Cr_2_O_7_ can effectively inhibit the growth of other bacteria and fungi in the soil, but has no inhibitory effect on actinomycetes. K_2_Cr_2_O_7_ can be used as an efficient and inexpensive inhibitor for the selective separation of actinomycetes, thereby increasing the isolation rate of actinomycetes (Hei et al., 2021). Briefly, soil suspensions were prepared at a mass-volume ratio of 10% (w/v), followed by serial gradient dilution (10^-^¹-10^-5^). Subsequently, 100 µL of each dilution was evenly spread onto Gao’s No. 1 agar plates supplemented with 50 ppm potassium dichromate, and incubated at 28 °C until single colonies were visible. Single colonies were then transferred to fresh Gao’s No. 1 agar plates (without potassium dichromate) pre-inoculated with a lawn of *C. haemulonii* 190070 (10^8^ CFU/mL). Colonies that generated distinct inhibition zones were selected as candidate antagonistic strains.

For preliminary taxonomic identification, the *16S rRNA* gene of the candidate antagonistic strain was amplified and sequenced using the universal primers 16S-F/R (detailed in **Table 2**).

**Table 2:** The list of the primers in this study.

Primers	Sequence(5’-3’)	Tm (°C)	Length (bp)
16S-F	AGAGTTTGATCCTGGCTCAG	55	1400
16S-R	CTACGGCTACCTTGTTACGA	55	1400
atpD-F	ATGACCACCACTGTTGAGACCGCGA	70	1400
atpD-R	TCAGGAGACGCCCAGCTCCTTGGCG	70	1400
gyrB-F	GTGGCCGATTCCGGCAACCCCAACG	60	2100
gyrB-R	TCAGATGTCGAGGAAGCGGACGTCC	60	2100
recA-F	ATGGCAGGAACCGACCGCGAGAAGG	65	1131
recA-R	TCAGCTCTTGGCCGCCGCGGCCTTG	65	1131
rpoB-F	TTGGCCGCCTCGCGCAATGCCTCGA	67	3486
rpoB-R	TCAGACCTCTTCGACGCTGCTCGGC	67	3486
2trpB-F	AGGACCTGAACCACACCGGCT	65	800
2trpB-R	TCGATGGCCGGGATGATGC	65	800

### Genome sequencing and feature analysis

Genomic DNA of strain NC-SA6 was extracted using a commercial bacterial genome extraction kit (Beyotime Biotechnology, China) in strict accordance with the manufacturer’s protocols. Whole-genome sequencing was conducted on the Oxford Nanopore PromethION sequencing platform. The detailed overview of genome sequencing are followings: ① DNA quality control and library construction; ② Second-generation sequencing (Illumina, obtain short-read sequencing) and third-generation (Nanopore, obtain long-read sequencing); ③ Sequencing data quality control to remove low-quality sequences and adapter sequences(ensure accuracy and reliability); ④ Generate the complete genome sequence (hybrid assembly and error correction with short-read and long-read data); ⑤ Analyze the genomic features. Gene prediction for coding sequences (CDS), tRNA, rRNA, and non-coding RNA was performed using Prodigal (v2.6.3), Aragorn (v1.2.38), RNAmmer (v1.2), and Infernal (v1.1), respectively. The secondary metabolic gene clusters were analyzed using the antiSMASH (version: 6.0.0). Functional annotation of predicted genes was carried out *via* BLAST homology searches against the Cluster of Orthologous Groups (COG) and Kyoto Encyclopedia of Genes and Genomes (KEGG) databases.

### Phylogenetic and genome-based species delineation analysis

A polyphasic taxonomic approach integrating MLSA and whole-genome comparative analysis was adopted to accurately identify the taxonomic status of the isolated strain. For MLSA analysis, specific primers targeting the *16S rRNA* gene and five housekeeping genes (*atpD*, *gyrB*, *recA*, *rpoB*, and *trpB*) were used (detailed in **Table 2**). Reference sequences of closely related type strains were retrieved from the GenBank database. MAFFT version 7 software was used for multiple sequence alignment, and MEGA X software was utilized to initially construct the phylogenetic tree. A maximum likelihood (ML) phylogenetic tree was constructed from based on the concatenated gene sequences using IQ-TREE 2.4.0, with 1000 bootstrap replicates to assess branch support values (Nguyen et al., 2014).

The complete genome sequence of strain NC-SA6 was compared with those of its closely related strains screened *via* MLSA analysis. The Average Nucleotide Identity (ANI) values between the query genomes and reference genomes were calculated using the JSpeciesWS online tool with the BLAST-based algorithm (Richter et al., 2016). Digital DNA-DNA Hybridization (dDDH) values were estimated using the Genome-to-Genome Distance Calculator (GGDC) web server (version 3.0) with the BLAST method (Meier-Kolthoff et al., 2022). The widely accepted species delineation thresholds (≥95-96% for ANI, ≥70% for dDDH) were applied to determine the species classification (Chun et al., 2018).

### Physiological and biochemical characterization

The physiological and biochemical characteristics of strain NC-SA6 were systematically determined as follows. Salt tolerance was evaluated by inoculating the strain onto Gao’s No. 1 medium supplemented with 0-13% (w/v) NaCl (with a gradient of 1%); pH tolerance was tested on medium adjusted to pH 4-13 (1-unit gradient increments). All plates were incubated at 28 °C for 7–14 days, and growth status was recorded daily. For temperature tolerance testing, the strain was cultured on Gao’s No. 1 medium at 4, 10, 16, 20, 25, 28, 30, 37, 40, and 45 °C for 7–14 days, and growth viability was assessed.

Carbon source utilization was evaluated on basal medium (2.38 g KH_2_PO_4_, 2.64 g (NH_4_)_2_SO_4_, 5.65 g K_2_HPO_4_, 1.0 g MgSO_4_·7H_2_O, 0.0079 g MnCl_2_·4H_2_O, 0.0064 g CuSO_4_·5H_2_O, 0.0015 g ZnSO_4_·7H_2_O, 0.0011 g FeSO_4_·7H_2_O per liter) supplemented with 0.5% of various carbon sources: inositol, D-trehalose, D-fructose, D-raffinose, D-sorbitol, D-galactose, D-mannitol, D-glucose, rhamnose, xylose, sucrose, ribose, arabinose, and maltose. Nitrogen source utilization was tested on screening medium (10 g D-glucose, 0.5 g MgSO_4_·7H_2_O, 0.5 g NaCl, 0.01 g FeSO_4_·7H_2_O per liter) amended with 0.5% of diverse nitrogen sources: L-asparagine, L-lysine, L-cysteine, L-threonine, L-valine, L-methionine, L-serine, D-arginine, and creatine. All cultures were incubated at 28 °C for 7–14 days, and substrate utilization was determined based on growth status.

Nitrate reduction, H_2_S production, and gelatin liquefaction were performed using commercial kits (Hopebio, HBIG14). Melanin production was evaluated on ISP6 and ISP7 media. Aesculin hydrolysis activity was tested on aesculin medium (10 g peptone, 1 g aesculin, 0.5 g ferric citrate, 5 g NaCl per liter). Milk coagulation and peptonization were assessed on milk medium (200 g milk powder, 0.2 g CaCO_3_ per liter). Catalase activity was determined by the observation of bubble generation after adding 3% H_2_O_2_ to fresh bacterial colonies.

### Optimization of fermentation conditions

To optimize the fermentation conditions for maximum antifungal metabolite production, different medium types and fermentation durations were screened under consistent culture temperature, shaking speed, and inoculum size. Tested media included ISP1, ISP2, ISP3, ISP4, No. 6 medium (20 g sucrose, 30 g soluble starch, 2 g peptone, 8 g soybean meal, 0.5 g MgSO_4_·7H_2_O, 0.5 g K_2_HPO_4_·3H_2_O, 2 g NaCl, and 3 g CaCO_3_ per liter), and No. 7 medium (20 g soybean meal, 2 g soy peptone, 20 g glucose, 5 g soluble starch, 5 g yeast extract powder, 4 g NaCl, 0.5 g K_2_HPO_4_, 0.5 g MgSO_4_, and 2 g CaCO_3_ per liter). Cultures were incubated at 28 °C and 200 rpm with 1% (CFU = 10^8^) inoculum size for 2–7 days. 1.5ml of the fermentation supernatants were collected by centrifuging at 13,000 rpm (rotor type: eppendrof, FA-24×2) for 5 min. and antimicrobial activity was quantitatively evaluated by measuring the diameter of the inhibition zone. Specifically, the holes were making on the plate containing the *C. haemulonii* 190070, then 50μL of the prepared fermentation supernatant were added to each holes, and the inhibition zone diameters were measured after cultivation (Sunanda et al., 2025).

### Measurement of broad-spectrum antimicrobial activity

The antimicrobial spectrum of NC-SA6 fermentation broth cultured in No.6 medium for 4 days (optimal condition) was tested against a panel of pathogenic strains, including yeast-like fungi, Gram-positive bacteria, and Gram-negative bacteria: *C. krusei* ATCC6258, *C. haemulonii* 190070, *C. auris* BJCA001, *C. albicans* SC5314, *Escherichia coli* ATCC25926, *Shigella flexneri* ATCC12022, *Pseudomonas aeruginosa* PA14, *Burkholderia cenocepacia* H111, *Bacillus velezensis* IF-1, *Bacillus cereus* ATCC14579, and *Staphylococcus aureus* ATCC29213. Meanwhile, positive control antifungal agent itraconazole(VRC) and antibacterial gentamicin (Gen) were mentioned. Antimicrobial activity was assessed by measuring the diameter of inhibition zones using the agar diffusion method (Sunanda and Ramesh Babu, 2025).

### The MIC values assay

Fermentation broth was centrifuged at 12000 rpm for 10 min at 4 °C. The supernatant was filtered through a 0.22-μm syringe filter. An equal volume of blank medium was processed as control. All the samples were pre-frozen at −80 °C for 24 h and lyophilized (shelf −40 °C, chamber pressure ≤5 pa) until dry. The resulting powder was weighed and reconstituted in sterile distilled water for the antimicrobial assay (Sornsenee et al., 2024). The MIC value was measured by using broth microdilution (according to the CLSI standard). The standard strains ATCC6258 and ATCC22019 were used as controls. RPMI 1640 was used to dilute the fungal (final 1×10^5^-5×10^5^ CFU/ml) and drug which perform 2-fold serial dilution (the maximum concentration of NC-SA6 fermentation and medium control were 73.6 mg/ml, 69.65 mg/ml, respectively). Then, the palte was incubated at 35°C for 24h. Finally, the MIC_50_ was recorded.

### Metabolomic analysis and identification of antifungal compounds

S. *anandii* NC-SA6 was cultured under the optimized fermentation conditions (28 °C, 200 rpm, 1% inoculation). Antimicrobial activity was dynamically monitored *via* the agar well diffusion assay. Fermentation supernatants at the highly active stage (4 days, three repetitions) and inactive stage (2 days, three repetitions) were collected, and intracellular and extracellular metabolites were extracted using a Starlid™ automated workstation. For polar metabolite analysis, LC-MS/MS detection was performed on a Vanquish UHPLC system (Thermo Fisher Scientific) coupled with an Orbitrap Exploris 120 mass spectrometer (Thermo Fisher Scientific), equipped with a Waters ACQUITY UPLC BEH Amide column (2.1 mm × 50 mm, 1.7 µm). MS/MS spectra were acquired in information-dependent acquisition (IDA) mode controlled by Xcalibur software. Raw data were converted to mzXML format using ProteoWizard software (V3.0.24054) for metabolite identification, with BiotreeDB (V3.0) as the database. The data management as following: ①Outliers were filtered based on the interquartile range. ②Missing value filtering: Individual features were filtered, retaining only peak area data where missing values accounted for no more than 50% in a single group or across all groups. ③Missing values in the raw data were imputated using half of the minimum value as the imputation method. ④Normalization was performed using the total ion current (TIC) of each sample. The final dataset containing the information of feature number, sample name and normalized feature area was imported to SIMCA18.0.1 software package for multivariate analysis. PCA (principle component analysis, PCA) was carried out to visualize the distribution and the grouping of the samples. Supervised orthogonal projections to latent structures-discriminant analysis (OPLS-DA) was performed to visualize group separation and screen significantly differential metabolites. Variable importance in projection (VIP) scores of the first principal component were calculated to evaluate the contribution of each metabolite. Metabolites with a VIP > 1 and Q-value < 0.05 (FDR corrected P-values) were defined as significantly differential metabolites. Pathway enrichment analysis was conducted using the KEGG database and MetaboAnalyst 6.0 online platform (https://www.metaboanalyst.ca/home.xhtml).

## Results

### Isolation and screening of an antagonistic *Streptomyces* strain against *C. haemulonii*

**Figure 1:**
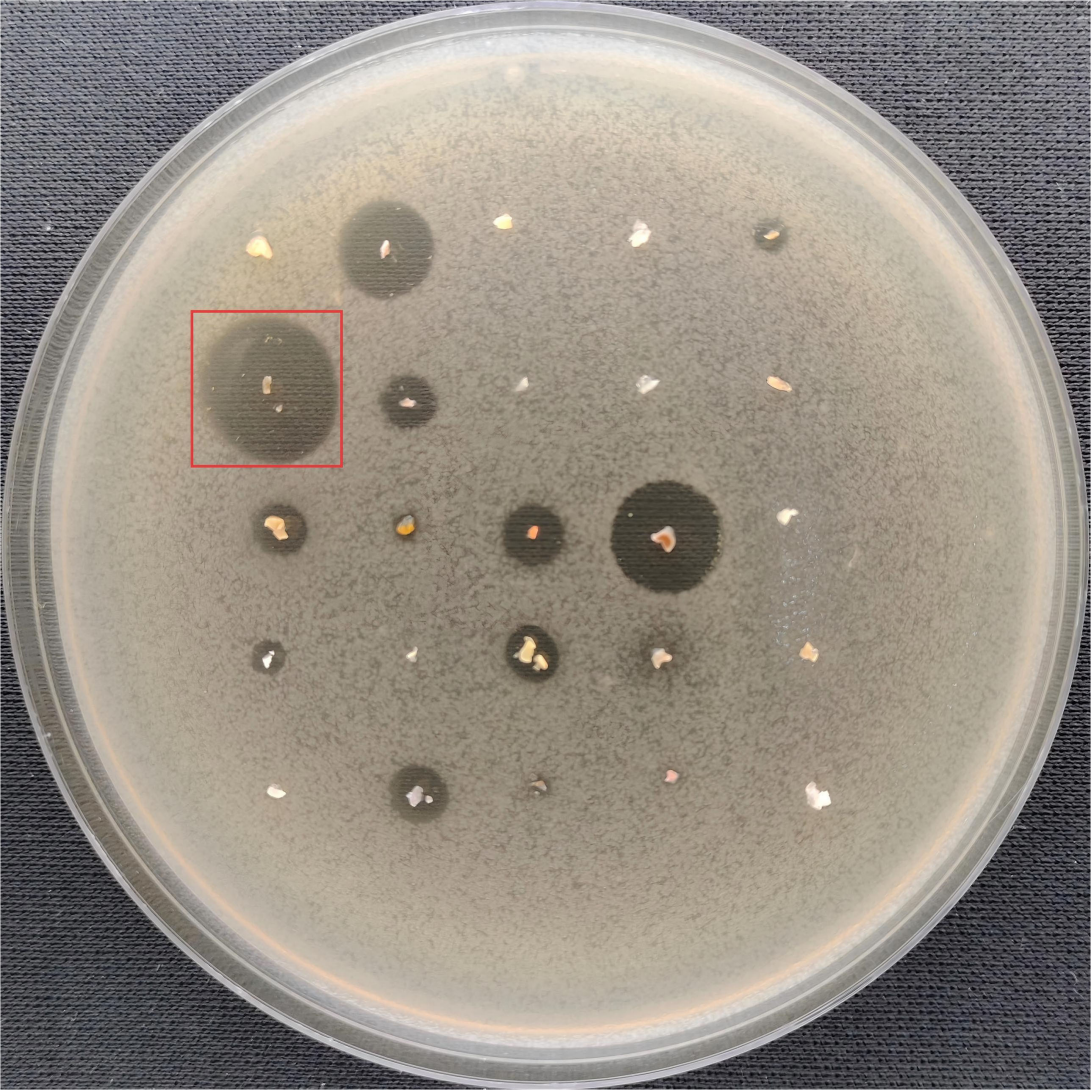
Preliminary screening of strains inhibiting *C. haemulonii*. Eight strains showed antifungal activity against *C. haemulonii* 190070. Isolate No. 6 (red box), designated *S. anandii* NC-SA6, produced the largest inhibition zone.

A total of 300 microbial isolates were obtained from soil samples in Nanchang, Jiangxi Province, China, using selective medium supplemented with 50 ppm potassium dichromate. Their antifungal activity against the multidrug-resistant pathogen *C. haemulonii* 190070 was screened *via* the plate confrontation assay. Among all isolates, eight strains generated distinct and stable inhibition zones against *C. haemulonii* (**Figure 1**). Based on colonial morphology, microscopic characteristics, and preliminary molecular identification, these active isolates were preliminarily classified into the genus *Streptomyces*. Notably, isolate No. 6 (strain NC-SA6) produced the largest inhibition zone with the most potent antagonistic activity (**Figure 1**), and was thus selected for further taxonomic identification and in-depth mechanistic research.

### Phylogenetic and genomic identification of strain NC-SA6 as *Streptomyces anandii*

**Figure 2:**
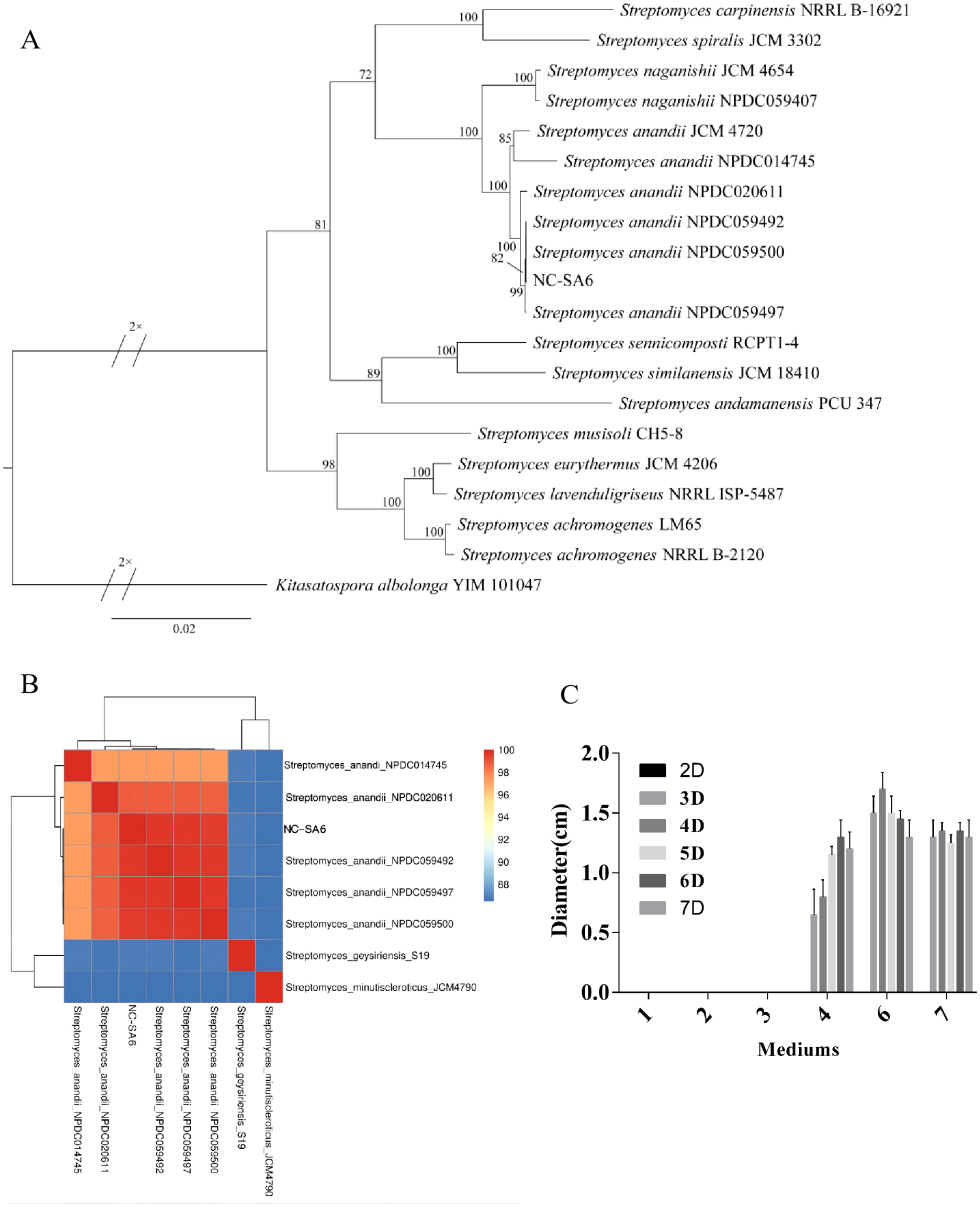
Identification and activity optimization of *S. anandii* NC-SA6. **(A)** Maximum likelihood phylogenetic tree. **(B)** ANIm heatmap. **(C)** Anti-*C. haemulonii* activity of *S. anandii* NC-SA6 in different media and at different fermentation times.

A polyphasic taxonomic strategy was employed to determine the precise taxonomic position of the highly antagonistic strain NC-SA6. First, a maximum likelihood phylogenetic tree was constructed based on the concatenated sequences of the *16S rRNA* gene and five housekeeping genes (*atpD*, *gyrB*, *recA*, *rpoB*, and *trpB*). Phylogenetic analysis revealed that strain NC-SA6 stably clustered with the type strains of *Streptomyces anandii*, with a bootstrap support value of 82% (**Figure 2A**), indicating its close phylogenetic relationship with *S. anandii*.

For definitive species-level classification, whole-genome comparative analysis was performed. The ANI values between strain NC-SA6 and five *S. anandii* strains were all above 97%, and the dDDH values were all higher than 70% (**Figure 2B**; **Table 3**). Both indices exceeded the widely accepted prokaryotic species delineation thresholds (95-96% for ANI, ≥70% for dDDH). Therefore, strain NC-SA6 was definitively identified as *Streptomyces anandii*, and was designated *S. anandii* NC-SA6 in subsequent studies.

**Table 3:** ANIm and dDDH values among 7 pairs of type *Streptomyces* species.

Query genome	Reference genome	dDDH	Prob. DDH >= 70%	ANIm
S. anandii NC-SA6	S.anandii_NPDC014745	74.3	84.88	97.23
S. anandii NC-SA6	S.anandii_NPDC020611	89.3	95.57	98.85
S. anandii NC-SA6	S.anandii_NPDC059492	96.1	97.47	99.6
S. anandii NC-SA6	S.anandii_NPDC059497	96.2	97.47	99.61
S. anandii NC-SA6	S.anandii_NPDC059500	95.9	97.42	99.58
S. anandii NC-SA6	S.geysiriensis_S19	26.9	0.02	86.77
S. anandii NC-SA6	S.minutiscleroticus_JCM4790	25.9	0.01	86.51

dDDH ≥70% and ANIm ≥95-96%,this indicates the two genomes belong to the same species.

### Physiological and biochemical characteristics of *S. anandii* NC-SA6

The physiological and biochemical properties of *S. anandii* NC-SA6 are comprehensively summarized in **Table 4**. The strain exhibited optimal growth at 28 °C, pH 7.0, and could tolerate NaCl concentrations up to 5% (w/v). It tested positive for nitrate reduction, aesculin hydrolysis, melanin and H2S production, milk coagulation, and milk peptonization, but negative for catalase activity and gelatin liquefaction. In terms of carbon source utilization, the strain could efficiently utilize inositol, D-trehalose, D-fructose, D-raffinose, D-sorbitol, D-glucose, and rhamnose, but could not utilize D-galactose, D-mannitol, xylose, or sucrose. For nitrogen source utilization, it could utilize L-cysteine, L-threonine, L-valine, L-methionine, L-serine, and creatine, but showed no utilization capacity for L-asparagine, L-lysine, or D-arginine.

**Table 4:** Physiological and biochemical characteristics of *S. anandii* NC-SA6.

Biolog	Result	Biolog	Result	Biolog	Result
Cell morphology		ribose	–	PH 7.0-9.0	+
Gram stain		arabinose	–	PH 10.0-13.0	–
0%-5% Nacl Salt tolerance test	++	maltose	–	phaseomannite	++
6%-13%Nacl Salt tolerance test	–	L-Asparagine	–	D-Trehalose	+
Temperature 4-10°C	–	L-lysine	–	D-fructose	++
Temperature 16-45°C	+	L-Cysteine	++	D-raffinose	++
Nitrate reduction	+	L-threonine	++	D-sorbitol	+
Gelatin liquefaction	–	L-valine	++	D-galactose	–
Milk coagulation and peptones	+	L-Methionine	++	D-mannitol	–
Hydrolysis test of aesculin	+	L-serine	++	D-glucose	++
H2S production	+	D-arginine	–	rhamnose	+
Melanin production	+	creatine	++	xylose	–
Contact enzyme test	–	PH 4.0-6.0	–	sucrose	–

++:grown well; +: growth or positive; -: ungrown or negative.

### Optimization of antifungal metabolite production

The antifungal activity of *S. anandii* NC-SA6 against *C. haemulonii* was significantly affected by culture medium type and fermentation duration (**Figure 2C**). The strongest antifungal activity was detected in the broth cultured in No. 6 medium for 4 days. Among the tested media, ISP4, No.6, and No.7 media supported the biosynthesis of active antifungal metabolites, whereas no detectable antifungal activity was observed in ISP1, ISP2, or ISP3 media. Specifically, the activity in ISP4 medium gradually increased from day 3 to day 7; the activity in No. 6 medium peaked sharply on day 4; and the activity in No. 7 medium remained stable from day 3 onwards. Accordingly, the 4-day fermentation broth in No. 6 medium was determined as the optimal condition, and was used for all subsequent activity validation and metabolomic analyses. The detailed means and SDs at different condotion were listed in **Table 5**.

**Table 5:** The values of mean±SD (figure 2C) at different conditions.

Medium/Time(day)	2D	3D	4D	5D	6D	7D
1	0	0	0	0	0	0
2	0	0	0	0	0	0
3	0	0	0	0	0	0
4	0	0.65±0.15	0.8±0.1	1.15±0.05	1.3±0.1	1.2±0.1
6	0	1.5±0.1	1.7±0.1	1.5±0.1	1.45±0.05	1.3±0.1
7	0	1.3±0.1	1.35±0.05	1.25±0.05	1.35±0.05	1.3±0.1

### *S. anandii* NC-SA6 exhibits broad-spectrum antimicrobial activity

**Figure 3:**
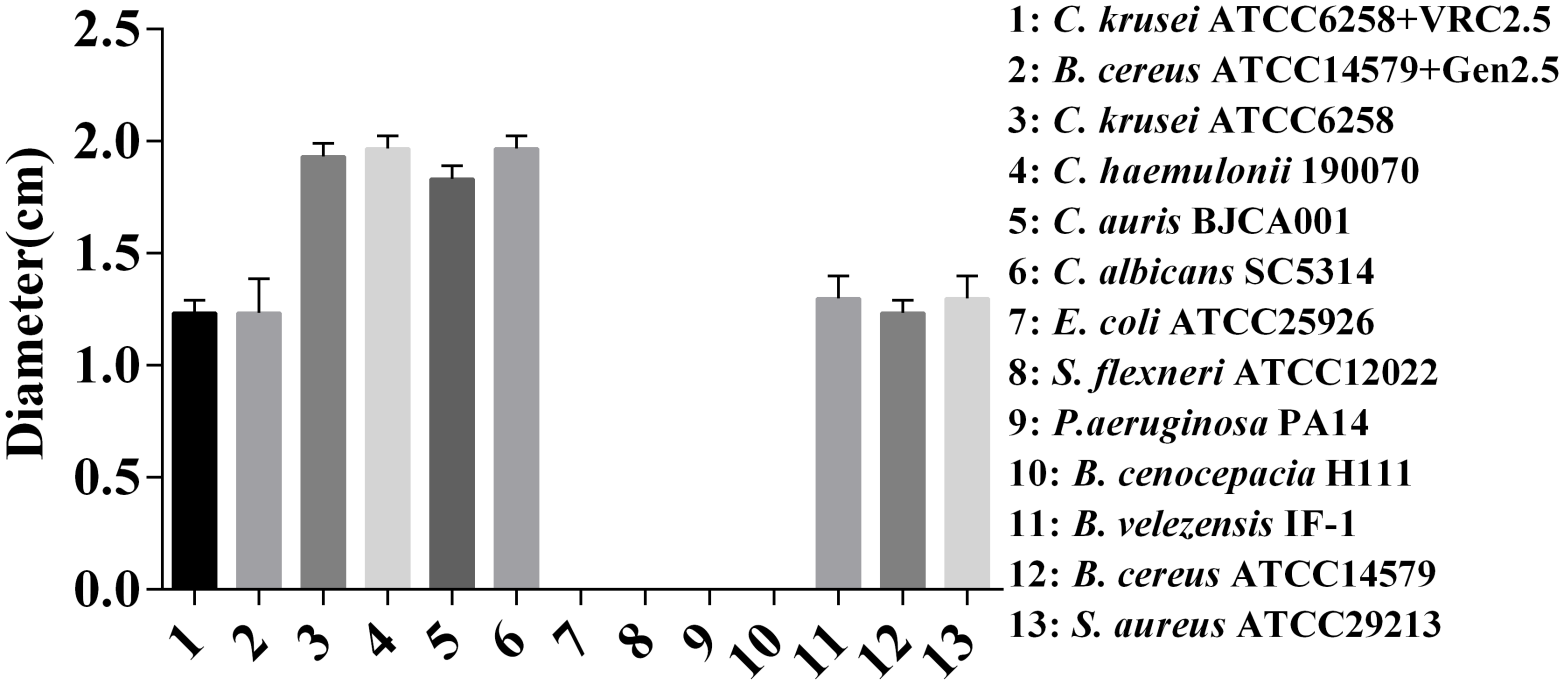
Antagonistic effect of *S. anandii* NC-SA6 against 11 pathogenic strains. Experiments were performed in triplicate, the average values and standard deviations of number 1–13 are 1.23 ± 0.05, 1.23 ± 0.12, 1.93 ± 0.05, 1.97 ± 0.05, 1.83 ± 0.05, 1.97 ± 0.05, 0 ± 0, 0 ± 0, 0 ± 0, 0 ± 0, 1.30 ± 0.08, 1.23 ± 0.05, 1.30 ± 0.08, respectively.

The antimicrobial spectrum of the optimal fermentation broth was evaluated against 11 clinically relevant pathogenic strains (**Figure 3**). The positive controls (*C. krusei* ATCC6258 + 2.5μg/ml VRC; *B. cereus* ATCC14579 + 2.5μg/ml Gen) showed a certain inhibition zones. And the fermentation filtrate displayed potent inhibitory activity against multiple pathogenic yeasts, including *C. krusei* ATCC6258, *C. haemulonii* 190070, *C. auris* BJCA001, and *C. albicans* SC5314. It also exerted remarkable antibacterial activity against Gram-positive bacteria, including *B. velezensis* IF-1, *B. cereus* ATCC14579, and *S. aureus* ATCC29213. In contrast, no inhibitory activity was observed against the tested Gram-negative bacteria, including *E. coli* ATCC25926, *S. flexneri* ATCC12022, *B. cenocepacia* H111, and *P. aeruginosa* PA14. Finally, we measured the MIC values of *S. anandii* NC-SA6 fermentation broth for *Candida auris* BJCA001 and *Candida haemulonii* 190070, the MIC_50_ values were 36.8 mg/ml and 18.4 mg/ml, respectively.

### Genomic insights into the biosynthetic potential of *S. anandii* NC-SA6

**Figure 4:**
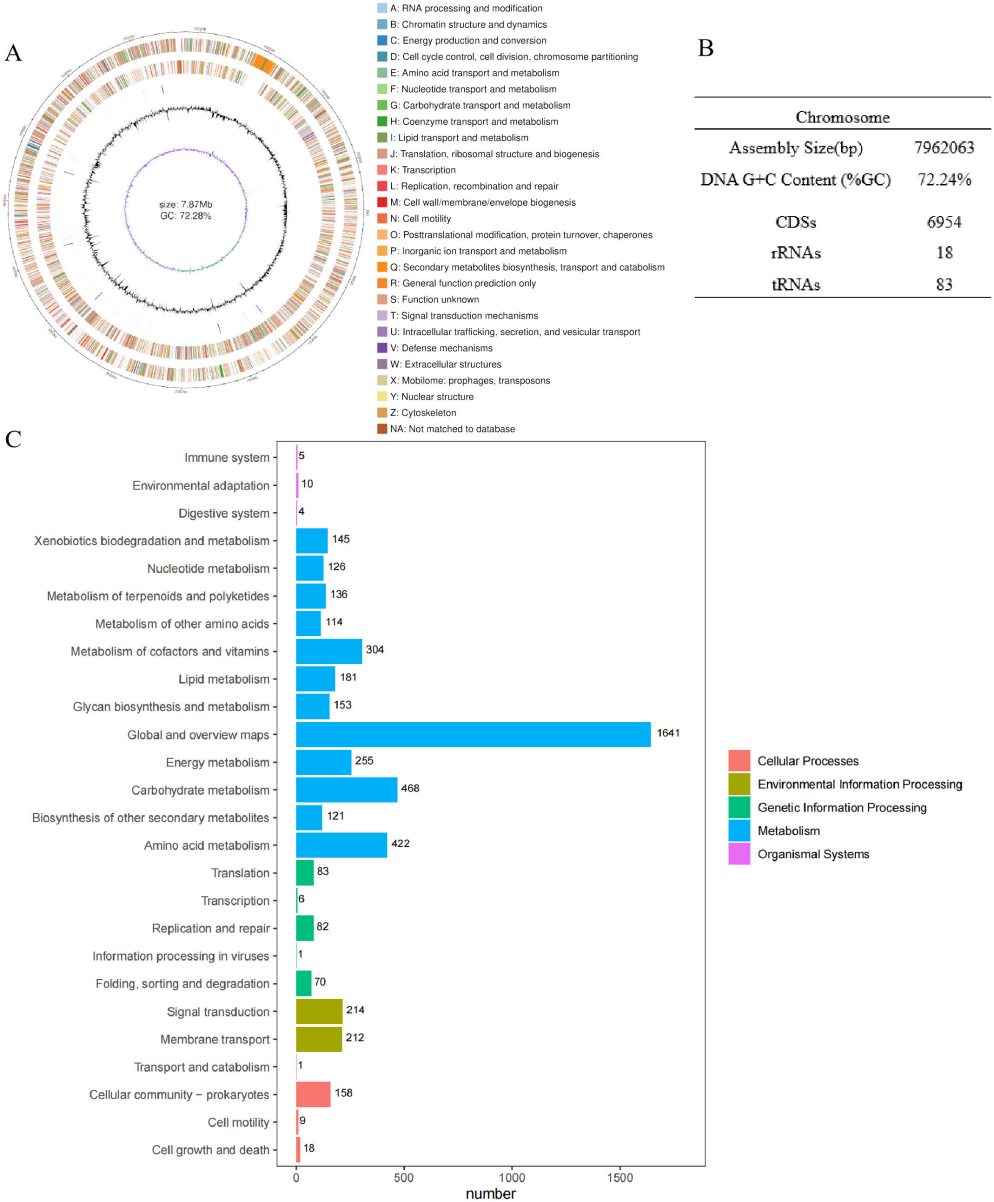
Genomic features of *S. anandii* NC-SA6. **(A)** Circular genome map. Circles from outside to center: genomic coordinates, forward-strand CDS, reverse-strand CDS, rRNA and tRNA genes, GC content, GC skew. **(B)** Genome assembly overview. **(C)** KEGG functional classification of genes.

The high-quality complete genome of *S. anandii* NC-SA6 was assembled into two contigs: a circular chromosome with a length of 7,867,603 bp and GC content of 72.28% (**Figure 4A**), and a circular plasmid with a length of 94,460 bp and GC content of 69.13%. A total of 7120 functional genes were predicted, including 6945 protein-coding genes (CDS), 83 tRNAs, 18 rRNAs, 1 tmRNA, and 73 miscellaneous RNAs (**Figure 4B**). Functional annotation results showed that 5057, 5518, and 5159 genes were successfully annotated to the KEGG, COG, and GO databases, respectively. KEGG functional classification revealed that the majority of predicted CDS were associated with metabolic pathways (82.3%), followed by environmental information processing (8.6%), genetic information processing (4.9%), cellular processes (3.8%), and organismal systems (0.4%) (**Figure 4C**).

A total of 19 secondary metabolite biosynthetic gene clusters were identified in the genome, encompassing three types of polyketide synthases (two type 1 PKS, two type 2 PKS, and one type 3 PKS), one non-ribosomal peptide synthase (NRPS), one ribosomally synthesized and post-translationally modified peptide (RiPPs) cluster, two melanin clusters, one ectoine cluster, two siderophore clusters, four terpene clusters, one butyrolactone cluster, and one lanthipeptide-class-iv cluster. Among these, 8 putative BGCs showed high similarity (>70%) to known functional clusters, 4 showed moderate similarity (30–70%), and 7 clusters exhibited low similarity (<30%), indicating their potential to encode novel secondary metabolites. The detailed information of homologous BGCs and similarity percentages is listed in **Table 6**, all information were from MIBiG database. The whole-genome sequence of *S. anandii* NC-SA6 has been deposited in GenBank under the accession number GCF_056171855.1.

**Table 6:** The list of secondary metabolism clusters prediction of *S. anandii* NC-SA6.

Compound	Synthetase type	Core genes	Size(Kb)	Most similar known cluster	Similarity	Bioactivity	BGC coordinates	MIBiG accession
melanin	melanin	iolS_1,adhA	8.7	melanin	57%	antimicrobial	1 - 6,497 nt	BGC0000908
herboxidiene	T3PKS	qorA_1,ahpF,gabD1,acdA_1,def_1,rocD_1	41.1	herboxidiene	7%	herbicidal, anti-cholesterol	3,785 - 90,616 nt	BGC0001065
ectoine	ectoine	mltF_1,ppaT,ectB,ectC,cefD	10.4	ectoine	100%	skin protection	1 - 3,366 nt	BGC0000853
melanin	melanin		10.5	melanin	60%	antimicrobial	1 - 6,625 nt	BGC0000909
desferrioxamin B / desferrioxamine E	siderophore	ddc_2,iucD_2,iucB,iucC	11.2	desferrioxamin B / desferrioxamine E	83%	antibacterial	3,024,895 - 3,054,682 nt	BGC0000940
spore pigment	lanthipeptide-class-iii,T2PKS	gpgS,lacC_2,nphA2,mnmC_2,mmcR,tcmI_1,dntB_1,cugP_3,ramS,ramC	70	spore pigment	83%	other	1 - 11,118 nt	BGC0000271
phosphonoglycans	NRPS	caeB_2,dhbF,tycC_2,ddl_1,asnB_2,thadh_2,hprA	49	phosphonoglycans	3%	Promote lipid metabolism	1 - 70,887 nt	BGC0000806
xantholipin	RRE-containing,T2PKS	pccB_1,lgrE,baiA,tcmI_2,fabG_6,garR_1,dntB_2,asnO_2,dntB_3,adoK_2,acs,mycG_1	72.5	xantholipin	44%	Cytotoxic, antibacterial	1 - 52,255 nt	BGC0000279
albaflavenone	terpene	def_2,cyc1	20.5	albaflavenone	100%	novel antibiotic	5,671,016 - 5,692,101 nt	BGC0000660
ficellomycin	siderophore	dat_2,iucA	10.7	ficellomycin	3%	antibiotic	11,780 - 55,969 nt	BGC0001593
geosmin	terpene	cyc2,pepE,treZ	21	geosmin	100%	other	6,656,219 - 6,678,399 nt	BGC0001181
ECO-02301	T1PKS,NRPS-like	ggt_1,map_2,argD_2,fgd_5,dltA_2,pikAV_1,elmGT,fadA_4-10,pikAI,eryA,fabD_2,strL,novW,cugP_4,strE_2,pikAV_2,lcfB_6,npt,pccB_3	196.2	ECO-02301	85%	Novel antifungal agent	1 - 161,719 nt	BGC0000052
tetronasin	T1PKS	yecD_1,fabH_2,Vejahgd,bioF_2,cmoM_3,dasA_3	44.5	tetronasin	3%	antibiotic	1 - 169,690 nt	BGC0000163
hopene	terpene	galE_3,glgC_1,aroB_2,crtB_1,hpnE,shc,moaA_2,dxs_2,pigE	25.9	hopene	92%	other	7,506,017 - 7,532,758 nt	BGC0000663
informatipeptin	RiPP-like	lagD	7.8	informatipeptin	42%	other	8,165,944 - 8,193,261 nt	BGC0000518
isorenieratene	RiPP-like,terpene	fabH_3,ilvB,crtB_2,crtI,crtY,ubiE	25.5	isorenieratene	75%	antioxidant	1 - 9,777 nt	BGC0001456
cyphomycin	butyrolactone	hldD_2,hsaA,menC	16	cyphomycin	5%	antimicrobial	423,393 - 622,305 nt	BGC0001877
rabelomycin / dehydrorabelomycin / fluostatin F, G, H	lanthipeptide-class-iv	iscS_3,maa,yfeW_3,pknD_22,cpo_2,nicF	22.4	rabelomycin / dehydrorabelomycin / fluostatin F, G, H	3%	other	1 - 66,898 nt	BGC0000223
colicin V	terpene	uppS	22.6	colicin V	1%	activate protein kinase C	15,749 - 19,808 nt	BGC0001555

### Metabolomic profiling identifies metabolites associated with bioactivity

**Figure 5:**
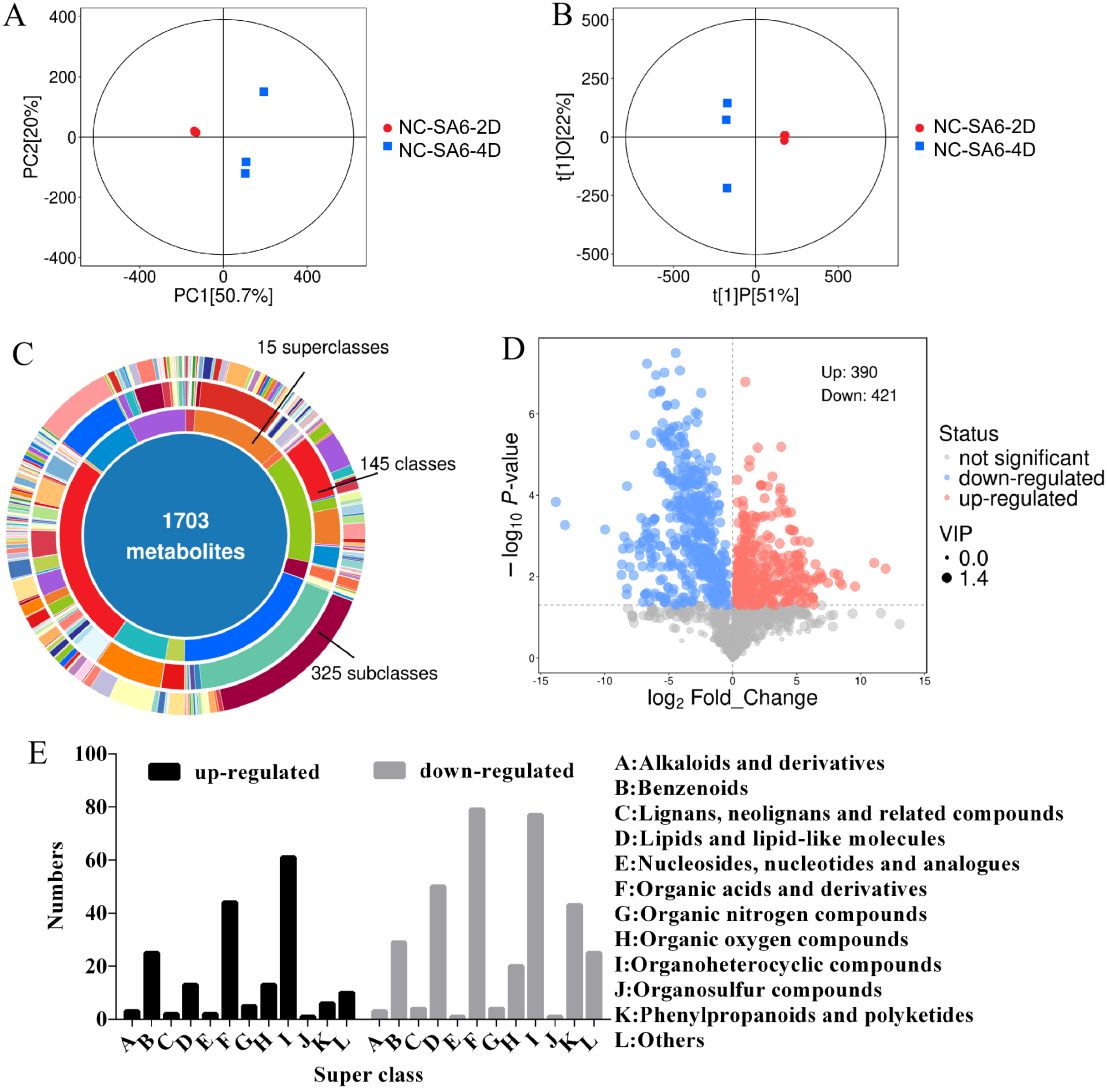
Secondary metabolite analysis of *S. anandii* NC-SA6. **(A)** Principal Component Analysis (PCA). **(B)** OPLS-DA Data. **(C)** Total number and classification of secondary metabolites. **(D)** Volcano plot of up- and down-regulated secondary metabolites. **(E)** Number and classification of up-regulated (black) and down-regulated (gray) secondary metabolites comparing active and inactive supernatants.

To correlate the observed antifungal activity with specific secondary metabolites, comparative LC-MS/MS-based untargeted metabolomic analysis was performed between the inactive (day-2) and highly active (day-4) fermentation broths. Firstly, we performed PCA analysis on the samples. As shown in **Figure 5A**, the PCA analysis revealed that all 6 samples fell within the 95% confidence interval (Hotelling’s T-squared ellipse). PC1 accounted for 50.7% of the variance, while PC2 contributed 20%. Three red samples clustered together. Although the blue group showed a deviation on PC2, the contribution rate of PC2 was relatively lower, and the samples were closely clustered on PC1 which had a higher contribution rate. OPLS-DA model validation parameters were listed in **Table 7**. In figure 5B, the abscissa t[1]P represents the predicted principal component score of the first principal component, showing the differences between sample groups, while the ordinate t[1]O represents the orthogonal principal component score, showing the differences within sample groups. The results indicate that there is a very small difference within groups, while the differences between groups are significant. Finally, a total of 1703 metabolites were identified and categorized into 15 superclasses, mainly classified into organoheterocyclic compounds, organic acids and their derivatives (**Figure 5C**; **Table 8**). Among 1703 metabolites, 402 metabolites belong to Level 1(confirmed with authentic standard), 1230 metabolites belong to Level 2; Level 2(matched to public library); 71 metabolites belong to Level 3 (matched to theoretical library). The volcano plot (**Figure 5D**) indicated that varying fermentation time significantly influenced metabolite production in *S. anandii* NC-SA6. The results of differential metabolite numbers showed significant alterations under varying fermentation time points, and these metabolic shifts contributed to the observed enhancement in antifungal activity (**Figure 5E**). Statistical analysis screened 521 significantly differential metabolites, with 185 up-regulated and 336 down-regulated in the active fermentation broth (VIP > 1, P-Value < 0.05, Q-Value < 0.05; **Figure 5E**). Among the 185 up-regulated metabolites, 5 metabolites have antimicrobial activity, and their mass spectra are shown in **Supplementary Figure 1**, and the detailed information were listed in **Supplementary Table S1**.

**Table 7:** The parameters of OPLS-DA model validation.

Type	A	N	R2X(cum)	R2Y(cum)	Q2(cum)
OPLS-DA	1+1+0	6	0.731	1.0	0.964

**Table 8:** The list of total secondary metabolites superclass of *S. anandii* NC-SA6.

Superclass	Freq	Percent
Organoheterocyclic compounds	430	25.25%
Organic acids and derivatives	331	19.44%
Lipids and lipid-like molecules	243	14.27%
Benzenoids	203	11.92%
Phenylpropanoids and polyketides	128	7.52%
Organic oxygen compounds	121	7.11%
Others	121	7.11%
Organic nitrogen compounds	41	2.41%
Nucleosides, nucleotides, and analogues	38	2.23%
Alkaloids and derivatives	21	1.23%
Lignans, neolignans and related compounds	17	1%
Organosulfur compounds	6	0.35%
Organic 1,3-dipolar compounds	1	0.06%
Organohalogen compounds	1	0.06%
Organophosphorus compounds	1	0.06%

**Figure 6:**
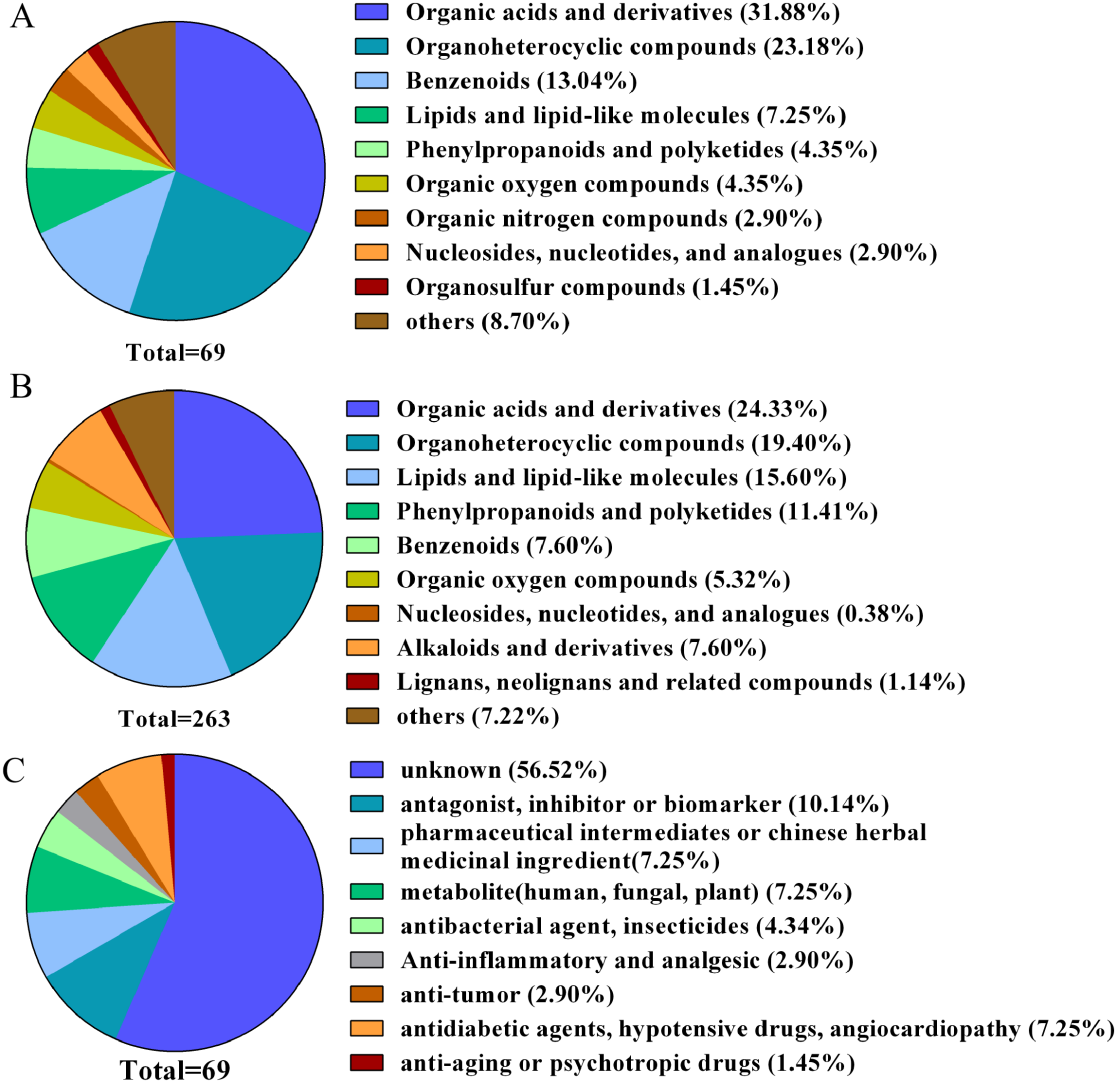
Analysis of metabolites with a fold-change >5 between active and inactive supernatants. **(A)** Number and classification of up-regulated secondary metabolites. **(B)** Number and classification of down-regulated secondary metabolites. **(C)** Functional analysis of up-regulated secondary metabolites.

In order to further study the functions of differential metabolites, especially up-regulated differential metabolites, we selected metabolites with differences of more than five times for analysis. Among the up-regulated and down-regulated compounds, organic acids and derivatives (31.88%, 23.18%) and organoheterocyclic compounds (24.33%, 19.40%) are both around 50% (**Figures 6A, B**), and the detailed information is listed in **Table 9** and **Table 10**, respectively. Then, we assigned functional categories using Chemical Book website according to the CAS number (**Tables 9**, **10**). We also classified the metabolites that were up-regulated by more than five times according to their functions, as shown in **Figure 6C**. Functional annotation demonstrated that 43.5% of the up-regulated metabolites were characterized as bioactive compounds, including antimicrobial agents, antifungal agents, and antitumor molecules; the remaining 56.5% were uncharacterized metabolites, representing potential novel bioactive secondary metabolites responsible for the strain’s antifungal and antibacterial activity. In conclusion, we revealed that *S. anandii* NC-SA6 is a promising producer of biologically important antibiotics and their novel analogs.

**Table 9:** The list of up-regulate Secondary metabolites (Fold change≥5).

Super.class	Level	m/z	rt	MS2 name	MS2 score	CAS	Description
Benzenoids	level2	257.1117	74.3	N-(2,6-Dimethylphenyl)-N'-phenylthiourea	2.58	25347-93-7	Inhibitors of phenol oxidase
Benzenoids	level2	287.0987	118.8	N-(3-Methoxyphenyl)-2-methyl-3-nitrobenzamide	2.44	123400-20-4	unknown
Benzenoids	level2	201.0857	188.5	Diphenylmethylphosphine	2.51	1486-28-8	unknown
Benzenoids	level2	273.083	116.6	2-Hydroxy-N-(4-methylphenyl)-5-nitrobenzamide	2.46	68507-96-0	unknown
Benzenoids	level2	233.0908	117.3	N-(1,3-Dioxolan-2-ylmethoxy)benzimidoyl cyanide	2.5	74782-23-3	unknown
Benzenoids	level2	332.0722	48.8	4-{2-[(5-Chloro-2-methoxybenzoyl)amino]ethyl}benzoic acid	2.18	54870-28-9	antidiabetic agents with hypoglycemic activity
Benzenoids	level2	344.1785	293.7	Clemastine	2.21	15686-51-8	unknown
Benzenoids	level2	223.1428	121.1	Neostigmine cation	2.65	59-99-4	A cholinesterase inhibitor
Benzenoids	level2	281.0529	76.7	Niflumic acid	2.29	4394-00-7	an analgesic and anti-inflammatory agent
Lipids and lipid-like molecules	level2	855.4709	99.8	Ophiopogonin D	2.2	945619-74-9	Related research of inflammation and cardiovascular diseases
Lipids and lipid-like molecules	level2	147.0656	31	(2R)-2,4-Dihydroxy-3,3-dimethylbutanoic acid	2.67	1112-33-0	unknown
Lipids and lipid-like molecules	level2	411.2704	151.8	3-Methoxylimaprost	2.07	1224443-96-2	3-methoxylimaprost is a prostanoid
Lipids and lipid-like molecules	level2	445.2568	147.5	Bufotalin	2.35	471-95-4	anti-tumor
Lipids and lipid-like molecules	level2	553.2778	260.6	Euphorbiasteroid	2.27	28649-59-4	Antibacterial
Nucleosides, nucleotides, and analogues	level1	312.1287	102.3	N2,N2-Dimethylguanosine	3.86	2140-67-2	It has a role as a human metabolite
Nucleosides, nucleotides, and analogues	level1	336.1649	29	Isopentenyladenosine	3.83	7724-76-7	Pharmaceutical Intermediates
Organic acids and derivatives	level2	187.154	162.2	3-(4-Methyl-1-piperazinyl)propanohydrazide	2.52	24636-93-9	unknown
Organic acids and derivatives	level2	301.2006	100	Arg-Lys	2.67	40968-46-5	unknown
Organic acids and derivatives	level2	157.0962	38.5	N-Acetyl-L-Prolinamide	2.63	16395-58-7	Pharmaceutical Intermediates
Organic acids and derivatives	level2	240.0951	188.6	(2S)-2-Amino-3-(2,4-diketo-6,7-dihydro-5H-cyclopenta[d]pyrimidin-1-yl)propionic acid	2.36	389888-02-2	unknown
Organic acids and derivatives	level1	141.0663	198.1	Ectoine	3.88	96702-03-3	Chinese herbal medicinal ingredient
Organic acids and derivatives	level1	172.0971	103	Acetylleucine	3.83	1188-21-2	Pharmaceutical Intermediates
Organic acids and derivatives	level2	160.0958	153.7	Betonicine	2.64	515-25-3	It has a role as a plant metabolite
Organic acids and derivatives	level1	218.1122	188.1	N-Acetylcitrulline	3.87	33965-42-3	unknown
Organic acids and derivatives	level1	427.2998	141.2	Leupeptin (hemisulfate)	3.78	103476-89-7	protease inhibitor
Organic acids and derivatives	level2	445.3106	145.3	2-Stearyl_citrate	2.21		unknown
Organic acids and derivatives	level2	269.1118	34.7	Serinyl-Tyrosine	2.21		unknown
Organic acids and derivatives	level1	217.1281	218	N-Acetyl-arginine	3.86	155-84-0	It has a role as a human metabolite
Organic acids and derivatives	level2	205.117	221.2	3-Methylpentane-2,4-diyl dicarbamate	2.57	5667-70-9	unknown
Organic acids and derivatives	level1	208.0956	89.8	N-Acetylphenylalanine	3.85	2018-61-3	anti-inflammatory agent
Organic acids and derivatives	level2	132.1123	232	1-(4-Aminobutyl)urea	2.61	6851-51-0	unknown
Organic acids and derivatives	level2	293.0729	49.5	Etrimfos	2.56	38260-54-7	Pesticides and insecticides
Organic acids and derivatives	level2	293.148	122.3	N2-(1-Oxo-4-phenylbutyl)-L-glutamine	2.27	491851-62-8	Phenylbutyrylglutamine is a glutamine derivative
Organic acids and derivatives	level1	265.1166	130.5	Phenylacetylglutamine	3.82	28047-15-6	It has a role as a human metabolite
Organic acids and derivatives	level2	211.1428	147.5	Cyclo(leucylprolyl)	2.64	5654-86-4	unknown
Organic acids and derivatives	level2	160.1071	199.6	Creatine, ethyl ester	2.62	15366-29-7	unknown
Organic acids and derivatives	level1	230.185	201.5	N1,N8-Diacetylspermidine	3.86	82414-35-5	unknown
Organic acids and derivatives	level1	247.1064	105.6	N-Acetyltryptophan	3.9	1218-34-4	It has a role as a metabolite
Organic nitrogen compounds	level2	201.1024	19.7	Pesticide4_Dinotefuran_C7H14N4O3_Guanidine, N-methyl-N''-nitro-N'-[(tetrahydro-3-furanyl)methyl]-	2.55	165252-70-0	Pesticides and insecticides
Organic nitrogen compounds	level1	142.0966	219.7	Histidinol	3.72	4836-52-6	glycylpeptide N-tetradecanoyltransferase inhibitor
Organic oxygen compounds	level2	396.2107	207.7	Spiperone	2.49	749-02-0	Small molecule inhibitors
Organic oxygen compounds	level2	290.21	20.6	1-Heptanone, 1-(4-methoxyphenyl)-2-(1-pyrrolidinyl)-	2.66	1801552-04-4	unknown
Organic oxygen compounds	level1	255.0958	221.4	Nicotinamide riboside (NR)	3.8	1341-23-7	anti-aging
Organoheterocyclic compounds	level2	285.0824	183.4	Nolatrexed	2.64	147149-76-6	anti-tumor
Organoheterocyclic compounds	level2	289.0318	48.3	5-Chloro-N-[4-(2-pyridinyl)-1,3-thiazol-2-yl]-2-pyridinamine	2.69	696628-24-7	unknown
Organoheterocyclic compounds	level2	207.073	185.9	5-(2,3-Difluorophenyl)-2-pyridinamine	2.73	875166-91-9	unknown
Organoheterocyclic compounds	level2	492.2404	129.3	6b-Hydroxy-3,14b,14c-trimethyl-5-oxo-2,3,4,5,6b,7,8,8a,9,14,14b,14c,15,16-tetradecahydro-4,16a-epoxy[1]benzoxocino[7',8':6,7]indeno[1,2-b]indol-3-yl acetate	2.18		unknown
Organoheterocyclic compounds	level2	409.2897	141.5	6a,7,10,10a-Tetrahydro-3-[5-(1H-imidazol-1-yl)-1,1-dimethylpentyl]-6,6,9-trimethyl-6H-dibenzo[b,d]pyran-1-ol	2.56	1347588-01-5	unknown
Organoheterocyclic compounds	level2	203.1169	19.9	2-(Piperidin-1-yl)benzo[d]oxazole	2.67	2851-09-4	unknown
Organoheterocyclic compounds	level2	251.141	292.8	Methyl (E)-3-[5-[(E)-4-hydroxy-3-methylbut-2-enyl]-1-methylimidazol-4-yl]prop-2-enoate	2.49	2066909-80-4	unknown
Organoheterocyclic compounds	level1	155.0091	124.7	Orotic acid	3.9	65-86-1	unknown
Organoheterocyclic compounds	level2	201.1123	110	2-[5-(2-Hydroxypropyl)oxolan-2-yl]propanoic acid	2.44	3688-54-8	unknown
Organoheterocyclic compounds	level1	161.07	27.2	SD-169	3.77	1670-87-7	treat diabetes
Organoheterocyclic compounds	level2	151.0606	62.8	5-(2-Furyl)-4H-1,2,4-triazol-3-amine	2.66	3663-61-4	unknown
Organoheterocyclic compounds	level2	310.1143	102.4	2,6-Bis(2-benzimidazolyl)pyridine	2.5	28020-73-7	unknown
Organoheterocyclic compounds	level2	470.1865	253.5	1-[(4-Chlorophenyl)methyl]-3-[(1,1-dimethylethyl)thio]-.alpha., .alpha.- dimethyl-5-(1-methylethyl)-1H-indole-2-propanoic acid	2.33	118414-82-7	Small molecule inhibitors
Organoheterocyclic compounds	level2	115.0858	232.1	4,4-Dimethyl-4,5-dihydro-1,3-oxazol-2-amine	2.6	52832-91-4	unknown
Organoheterocyclic compounds	level2	256.0801	195.9	6-Methyl-3-phenylpyrimido[5,4-e][1,2,4]triazine-5,7(6H,8H)-dione	2.1	42285-76-7	unknown
Organoheterocyclic compounds	level2	557.267	265.3	Dipiperamide_C	2.35		unknown
Organosulfur compounds	level2	184.0556	41.2	3-(Allylsulfanyl)-6-methyl-1,2,4-triazin-5-ol	2.56	87450-64-4	unknown
Phenylpropanoids and polyketides	level2	291.0938	34.4	(2R,3R)-2-(3,4-dihydroxyphenyl)-3,4-dihydro-2H-chromene-3,5,7-triol	2.53	490-46-0	Small molecule inhibitors, anti-inflammatory
Phenylpropanoids and polyketides	level2	271.0909	49.5	Alloimperatorin	2.7		unknown
Phenylpropanoids and polyketides	level2	212.0905	122.3	Methyldopa	2.65	555-30-6	hypotensive drugs
Others	level2	883.5021	99.5	(3.beta., 5.Xi., 9.Xi.)-23-Hydroxy-3-((.beta.-D-xylopyranosyl-(1->3)-6-deoxy-.alpha.-L-mannopyranosyl-(1->2)-.alpha.-L-arabinopyranosyl)oxy)olean-12-en-28-oic acid	2.27		unknown
Others	level3.1	459.3263	137.8	N-Eicosapentaenoyl Arginine	1.69		unknown
Others	level3.1	441.3159	136.1	N-[1-[[5-(Diaminomethylideneamino)-1-oxopentan-2-yl]amino]-4-methyl-1-oxopentan-2-yl]-4-methyl-2-(propanoylamino)pentanamide	1.75		unknown
Others	level3.2	457.3105	155.9	M457T156	1.77		unknown
Others	level2	615.2713	251.7	Norethindrone 2,3,4-tri-O-acetyl-.beta.-D-glucuronide methyl ester	2.03		unknown
Others	level3.1	270.1431	148	1-[2-[[Pyrrolidine-2-carbonyl]amino]acetyl]pyrrolidine-2-carboxylic Acid	1.73		unknown

**Table 10:** The list of down-regulate Secondary metabolites (Fold change≥5) .

Super.class	level	mz	rt	MS2 name	MS2 score	CAS	Description
Alkaloids and derivatives	level2	460.1651	212.2	Hydromorphone-3-.beta.-D-glucuronide	2.13	40505-76-8	unknown
Alkaloids and derivatives	level2	779.4114	271.9	Vinorelbine	2.48	71486-22-1	Antitumor drugs
Benzenoids	level2	432.0994	39.3	Nonanamide, N-[(4-hydroxy-2-iodo-5-methoxyphenyl)methyl]-8-methyl-	2.09	1177195-52-6	unknown
Benzenoids	level2	428.2596	224.3	Ranolazine	2.53	95635-55-5	Small molecule inhibitors
Benzenoids	level1	269.0445	19.7	Emodin	3.92	518-82-1	Antitumor drugs
Benzenoids	level1	431.0969	110.2	Emodin-8-glucoside	3.65	38840-23-2	Chinese herbal medicinal ingredient
Benzenoids	level2	436.2141	183.5	Benzoic acid, 4-hydroxy-3-[[2-(4-tricyclo[3.3.1.1(3,7)]dec-1-ylphenoxy)acetyl]amino]-, methyl ester	2.18	934593-90-5	inhibitors
Benzenoids	level1	161.1064	119.2	Tolazoline (hydrochloride)	3.65	59-98-3	Pharmaceutical Intermediates
Benzenoids	level2	320.1747	273.9	Imidafenacin	2.21	170105-16-5	Pharmaceutical Intermediates
Benzenoids	level2	344.0385	191.7	Phenol, 2-[4-[(3,5-dichlorophenyl)amino]-6-methyl-2-pyrimidinyl]-	2.62	381683-04-1	unknown
Benzenoids	level1	138.0905	136	Tyramine	3.74	51-67-2	Pharmaceutical Intermediates
Benzenoids	level2	397.1459	197.9	Kadethrine	2.22	58769-20-3	pesticide
Benzenoids	level2	353.1202	201.3	(3Z,5E)-3,5-Bis(4-methoxybenzylidene)tetrahydro-4H-thiopyran-4-one	2.25		unknown
Benzenoids	level2	321.17	254.3	[2-[4-Methyl-2-(2-methylpropanoyloxy)phenyl]oxiran-2-yl]methyl 2-methylpropanoate	2.73	22518-06-5	Antibacterial
Benzenoids	level2	357.1764	198.1	[(1S)-1-Benzyl-2-[[5-(3-methyl-2H-indazol-5-yl)-3-pyridyl]oxy]ethyl]amine	2.55	552325-73-2	Small molecule inhibitors, Signal transduction pathway kinase inhibitors
Benzenoids	level2	268.1271	181.2	4-tert-Butyl-2,6-dimethyl-3,5-dinitroaniline	2.39	107342-55-2	unknown
Benzenoids	level2	286.0774	54.7	(2,6-Dichlorophenyl)(3,5-dimethylpiperidin-1-yl)methanone	2.65	346724-91-2	unknown
Benzenoids	level2	249.1435	269	N-Cycloheptyl-N'-phenylthiourea	2.59	102936-60-7	unknown
Benzenoids	level2	144.08	118.7	2-Aminonaphthalene	2.65	91-59-8	unknown
Benzenoids	level2	319.1594	203.8	Fluvoxamine	2.45	54739-18-3	Medication for the nervous system
Benzenoids	level1	137.0237	86.4	4-Hydroxybenzoic acid	3.81	99-96-7	Small molecule inhibitors
Benzenoids	level1	137.0237	86.4	3-Hydroxybenzoic acid	3.73	36320	Novel selective and partial agonists of 5-HT3 receptors
Lignans, neolignans and related compounds	level2	481.108	33.1	2,3-Dehydrosilybin	2.55	25166-14-7	used in the treatment of hepatitis; liver cirrhosis; and chemical and drug induced liver injury
Lignans, neolignans and related compounds	level2	419.2119	266.4	2-(4-Allyl-2,6-dimethoxyphenoxy)-1-(3,4,5-trimethoxyphenyl)-1-propanol	2.12	41551-58-0	unknown
Lignans, neolignans and related compounds	level2	362.2003	270.9	4-[5-(4-hydroxy-3-methoxyphenyl)-3,4-dimethyloxolan-2-yl]-2-methoxyphenol	2.07	112652-46-7	unknown
Lipids and lipid-like molecules	level1	214.0476	230.2	Glycerophosphoethanolamine	3.81	1190-00-7	Active candidate
Lipids and lipid-like molecules	level2	477.2681	152.6	Betamethasone-17-valerate	2.2		unknown
Lipids and lipid-like molecules	level2	543.1235	38.2	(1aR,5S)-5b-((Benzoyloxy)methyl)-5a-(.beta.-D-glucopyranosyloxy)-5-methylhexahydro-2H-2,5-methano-3,4-dioxacyclobuta[cd]pentalene-2-sulfonic acid	2.09		unknown
Lipids and lipid-like molecules	level2	476.2745	130.8	LPE(18:3)	2.64		unknown
Lipids and lipid-like molecules	level1	258.1083	226.9	Glycerophosphocholine	3.86	28319-77-9	as a parasympatholytic, a neuroprotective agent
Lipids and lipid-like molecules	level2	311.2214	27.7	(9Z,12E)-15,16-Dihydroxyoctadeca-9,12-dienoic acid	2.66		unknown
Lipids and lipid-like molecules	level2	478.2905	124.7	PE(18:2(9Z,12Z)/0:0)	2.23	85046-18-0	as a human metabolite
Lipids and lipid-like molecules	level1	277.216	17.9	alpha-Linolenic acid	3.91	463-40-1	Shown to have an antithrombotic effect. It has a role as a micronutrient, a nutraceutical and a mouse metabolite.
Lipids and lipid-like molecules	level1	277.216	17.9	gamma-Linolenic acid	3.9	506-26-3	It has a role as a human metabolite, a plant metabolite and a mouse metabolite
Lipids and lipid-like molecules	level2	518.3214	123.1	LysoPC(18:3(6Z,9Z,12Z))	2.34		unknown
Lipids and lipid-like molecules	level2	375.219	22.6	Resolvin_D2	2.69	82864-77-5	unknown
Lipids and lipid-like molecules	level1	313.237	26.2	Octadecanedioic acid	3.66	871-70-5	Pharmaceutical Intermediates
Lipids and lipid-like molecules	level1	520.3371	121.8	LPC(18:2/0:0)	3.81	22252-07-9	It has a role as a mouse metabolite
Lipids and lipid-like molecules	level2	667.3284	287.1	14,16,17,20-Tetrahydroxy-1,26-dioxo-22,26-epoxyergosta-5,24-dien-3-yl .beta.-D-glucopyranoside	2.18		unknown
Lipids and lipid-like molecules	level2	427.2274	258.8	Cinnzeylanine	2.06	62203-47-8	unknown
Lipids and lipid-like molecules	level2	465.2042	208.7	Beclomethasone 21-propionate	2.3	69224-79-9	unknown
Lipids and lipid-like molecules	level2	555.3107	188.7	FUSIDIC ACID	2.18	6990-06-3	It has a role as a protein synthesis inhibitor
Lipids and lipid-like molecules	level2	337.1829	125.3	3-(2-Hydroxyethyl)-4-(hydroxymethyl)hex-5-en-1-yl .beta.-D-glucopyranoside	2.12		unknown
Lipids and lipid-like molecules	level2	495.215	209.7	Fluocinonide	2.13	356-12-7	as a Corticosteroid Hormone Receptor Agonist
Lipids and lipid-like molecules	level2	528.3113	243.6	LysoPE(22:5(7Z,10Z,13Z,16Z,19Z)/0:0)	2.21		unknown
Lipids and lipid-like molecules	level2	526.2398	223.9	Sonolisib	2.17	502632-66-8	small-molecule wortmannin analogue inhibitor
Lipids and lipid-like molecules	level2	554.3422	141.6	3,5,9-Trioxa-4-phosphatetracosan-1-aminium, 7-(acetyloxy)-24-carboxy-4-hydroxy-N,N,N-trimethyl-, inner salt, 4-oxide, (R)-	2.54	129879-41-0	unknown
Lipids and lipid-like molecules	level2	309.2055	23.9	FA 18:3+2O	2.69		unknown
Lipids and lipid-like molecules	level2	359.2188	22.3	12-(Acetyloxy)pimara-7,15-dien-18-oic acid	2.56		unknown
Lipids and lipid-like molecules	level1	279.2315	19.5	cis-​9,​10-​Epoxystearic acid	3.84	24560-98-3	unknown
Lipids and lipid-like molecules	level2	335.2177	19.5	Kaurane-17,18-dioic acid	2.55	58648-80-9	unknown
Lipids and lipid-like molecules	level2	483.2705	55.3	1-Palmitoyl-2-hydroxy-sn-glycero-3-phospho-(1'-rac-glycerol)	2.65	749875-10-3	unknown
Lipids and lipid-like molecules	level2	298.1007	236.9	L-Menthone_1,2-glycerol_ketal	2.73	63187-91-7	unknown
Lipids and lipid-like molecules	level2	526.2955	275.1	PE(22:6(4Z,7Z,10Z,13Z,16Z,19Z)/0:0)	2.22		unknown
Lipids and lipid-like molecules	level2	337.2344	18.9	Prostaglandin F2.alpha. 1,15-lactone	2.55	55314-49-3	unknown
Lipids and lipid-like molecules	level2	295.2252	53.5	13-HOTE	2.47		unknown
Lipids and lipid-like molecules	level2	401.2316	251.8	Lucidone_B	2.64	97653-93-5	Chinese herbal medicinal ingredient
Lipids and lipid-like molecules	level2	391.2172	246.8	Neoquassin	2.16	76-77-7	Chinese herbal medicinal ingredient
Lipids and lipid-like molecules	level2	632.3121	293.8	Mesaconitine	2.6		unknown
Lipids and lipid-like molecules	level2	529.2292	258.4	Dutasteride	2.72	164656-23-9	as an EC 1.3.1.22 [3-oxo-5alpha-steroid 4-dehydrogenase (NADP(+))] inhibitor and an antihyperplasia drug
Lipids and lipid-like molecules	level2	471.2192	200.1	Benzoic acid, 4-[[(1R,2E,4E,6Z,9Z)-1-[(1S)-4-carboxy-1-hydroxybutyl]-2,4,6,9-pentadecatetraen-1-yl]thio]-	2.18	154978-38-8	as a leukotriene antagonist
Lipids and lipid-like molecules	level2	359.2267	151.7	Piperoic_acid	2.07	110979-04-9	unknown
Lipids and lipid-like molecules	level2	539.2628	284.8	Lucidenic Acid E2	2.25	98665-17-9	Chinese herbal medicinal ingredient
Lipids and lipid-like molecules	level2	529.2826	286	17-(Furan-3-yl)-3-hydroxy-4,4,8-trimethyl-16-oxo-14,15-epoxyandrostane-1,7-diyl diacetate	2.21	1273559-94-6	unknown
Lipids and lipid-like molecules	level2	371.2266	157.2	17-Phenyltrinor-13,14-dihydroprostaglandin A2	2.11	130209-80-2	unknown
Lipids and lipid-like molecules	level2	497.2693	204	Leukotriene_D4	2.24	73836-78-9	as a human metabolite, a bronchoconstrictor agent and a mouse metabolite
Nucleosides, nucleotides, and analogues	level1	227.0662	42.8	2'-Deoxyuridine	3.85	951-78-0	small-molecule inhibitor
Organic acids and derivatives	level1	150.0573	170.5	Methionine	3.87	59-51-8	Pharmaceutical Intermediates
Organic acids and derivatives	level2	293.1131	204.8	Phe-Glu	2.51	3617-45-6	It has a role as a metabolite
Organic acids and derivatives	level1	295.1273	205.8	gamma-Glutamylphenylalanine	3.88	7432-24-8	It has a role as a human urinary metabolite
Organic acids and derivatives	level1	166.0851	155.4	Phenylalanine	3.84	63-91-2	alkaline phosphatase inhibitor
Organic acids and derivatives	level1	132.101	157.2	Leucine	3.87	61-90-5	Chinese herbal medicinal ingredient
Organic acids and derivatives	level1	132.101	157.2	Isoleucine	3.85	73-32-5	It is important in hemoglobin synthesis and regulation of blood sugar and energy levels.
Organic acids and derivatives	level1	132.101	157.2	Norleucine	3.84	104809-14-5	unknown
Organic acids and derivatives	level1	145.0611	201.3	Ala-Gly	3.82	687-69-4	unknown
Organic acids and derivatives	level1	147.0755	201.2	Gly-Ala	3.87	3695-73-6	It has a role as a metabolite
Organic acids and derivatives	level1	147.0755	201.2	N-Methyl-L-asparagine	3.74	7175-34-0	unknown
Organic acids and derivatives	level1	134.0439	234.1	Aspartate	3.87	56-84-8	Pharmaceutical Intermediates
Organic acids and derivatives	level2	328.2209	144	Diprotin B	2.39	90614-49-6	Antibiotics
Organic acids and derivatives	level2	264.0865	42.2	3,4-Dihydroxycinnamic acid (L-alanine methyl ester) amide	2.66	778624-05-8	unknown
Organic acids and derivatives	level2	175.1066	177.9	Val-Gly	2.64	686-43-1	It has a role as a metabolite
Organic acids and derivatives	level1	203.0663	242.4	Glycyl-glutamate	3.87	7412-78-4	It has a role as a metabolite
Organic acids and derivatives	level1	246.1546	247.6	Arg-Ala	3.83	40968-45-4	It has a role as a metabolite
Organic acids and derivatives	level1	263.1374	142.1	Pro-Phe	3.87	13589-02-1	It has a role as a metabolite
Organic acids and derivatives	level2	189.1221	170.6	Ile-Gly	2.63	868-28-0	It has a role as a metabolite
Organic acids and derivatives	level1	118.0504	208.5	Homoserine	3.86	672-15-1	It has a role as a metabolite
Organic acids and derivatives	level1	189.1334	292	Homoarginine	3.86	156-86-5	It has a role as an EC 3.1.3.1 (alkaline phosphatase) inhibitor
Organic acids and derivatives	level2	232.139	256.3	Gly-Arg	2.62	18635-55-7	It has a role as a metabolite
Organic acids and derivatives	level1	187.1078	163.8	Glycylleucine	3.9	869-19-2	It has a role as a metabolite
Organic acids and derivatives	level1	180.0657	181.1	Tyrosine	3.82	60-18-4	It has a role as a metabolite
Organic acids and derivatives	level1	180.0657	181.1	o-Tyrosine	3.58	2370-61-8	small-molecule inhibitor
Organic acids and derivatives	level2	900.4184	239.1	Lyciumin_D	2.32	150415-40-0	unknown
Organic acids and derivatives	level2	248.1226	191.9	Thr-Gln	2.52	96337-79-0	It has a role as a metabolite
Organic acids and derivatives	level2	457.1121	33.2	Raltitrexed	2.46	112887-68-0	an antimetabolite used in chemotherapy. an inhibitor of thymidylate synthase
Organic acids and derivatives	level1	104.0699	219.3	4-Aminobutyric acid (GABA)	3.87	56-12-2	It has a role as a signalling molecule, a neurotransmitter.
Organic acids and derivatives	level2	281.0994	154.2	Methionyl-Methionine	2.61		unknown
Organic acids and derivatives	level1	90.0544	205.5	Alanine	3.85	56-41-7	EC 4.3.1.15 (diaminopropionate ammonia-lyase) inhibitor
Organic acids and derivatives	level1	90.0544	205.5	Sarcosine	3.8	107-97-1	It has a role as a glycine transporter 1 inhibitor, a glycine receptor agonist
Organic acids and derivatives	level1	274.1032	236.7	gamma-Glutamylglutamine	3.86	10148-81-9	It has a role as a human metabolite
Organic acids and derivatives	level1	130.0866	161.5	3-Amino-4-methylpentanoic acid	3.86	5699-54-7	It has a role as a human metabolite
Organic acids and derivatives	level1	120.0647	207.5	Threonine	3.87	72-19-5	Pharmaceutical Intermediates
Organic acids and derivatives	level2	116.0711	178.8	3-Aminopentanoic acid	2.66	18664-78-3	unknown
Organic acids and derivatives	level2	644.322	225.2	S-(PGA1)-glutathione	2.4		unknown
Organic acids and derivatives	level1	132.065	205	4-Hydroxyproline	3.92	51-35-4	It has a role as a metabolite
Organic acids and derivatives	level2	329.1913	268.9	Gly-Pro-Arg	2.63	47295-77-2	unknown
Organic acids and derivatives	level2	246.1084	192.7	Gln-Thr	2.55	74408-69-8	unknown
Organic acids and derivatives	level2	229.1532	147.5	Ile-Pro	2.64	37462-92-3	It has a role as a metabolite
Organic acids and derivatives	level1	175.1176	295.9	Arginine	3.75	74-79-3	It has a role as a nutraceutical, a biomarker
Organic acids and derivatives	level2	272.1701	264.3	Pro-Arg	2.61	2418-74-8	It has a role as a metabolite
Organic acids and derivatives	level1	133.0135	233.2	Malic acid	3.88	6915-15-7	It has a role as a food acidity regulator and a fundamental metabolite
Organic acids and derivatives	level2	193.0222	35.2	[3-(Trifluoromethyl)-1H-pyrazol-1-yl]acetic acid	2.35	926241-24-9	unknown
Organic acids and derivatives	level2	246.1433	179.9	Ile-Asn	2.58	59652-59-4	It has a role as a metabolite
Organic acids and derivatives	level1	244.129	179.8	Gly-Gly-Leu	3.84	14857-82-0	It has a role as a metabolite
Organic acids and derivatives	level1	116.0698	183.2	Proline	3.87	147-85-3	as a micronutrient, a nutraceutical
Organic acids and derivatives	level2	255.1438	188.9	His-Val	2.56	76019-15-3	It has a role as a metabolite
Organic acids and derivatives	level1	173.0923	185.3	Gly-Val	3.83	1963-21-9	It has a role as a human metabolite
Organic acids and derivatives	level1	173.0923	185.3	N5-acetyl-l-ornithine	3.68	2185-16-2	unknown
Organic acids and derivatives	level2	172.0593	182.8	2-Amino-5,5,5-trifluoropentanoic acid	2.61	2365-80-2	unknown
Organic acids and derivatives	level2	274.1857	224.3	Arg-Val	2.58	2896-20-0	It has a role as a metabolite
Organic acids and derivatives	level2	269.1592	180.1	His-Ile	2.64	129050-48-2	unknown
Organic acids and derivatives	level2	497.2804	269.1	APGPR_Enterostatin	2.38	117830-79-2	can reduce fat intake
Organic acids and derivatives	level1	156.0758	239.4	Histidine	3.7	71-00-1	Pharmaceutical Intermediates
Organic acids and derivatives	level1	213.097	221.4	Gly-His	3.82	2489-13-6	It has a role as a metabolite
Organic acids and derivatives	level1	163.1067	293.5	5-Hydroxylysine	3.86	1190-94-9	It has a role as a human metabolite
Organic acids and derivatives	level1	147.1119	299.9	Lysine	3.81	56-87-1	unknown
Organic acids and derivatives	level2	182.08	188.7	Amino(4-methoxyphenyl)acetic acid	2.36	19789-59-4	Pharmaceutical Intermediates
Organic acids and derivatives	level2	213.0162	44.4	2-Deoxyribose 5-phosphate	2.3	7685-50-9	It has a role as a metabolite
Organic acids and derivatives	level2	188.1381	59	N6-(1-Iminoethyl)-L-lysine	2.18	53774-63-3	Inhibitors of inducible nitric oxide synthase
Organic acids and derivatives	level2	525.3097	178.9	VPGPR_Enterostatin	2.34	144964-56-7	unknown
Organic acids and derivatives	level2	276.1537	174	Lys-Glu	2.54	45234-02-4	flavouring agent
Organic acids and derivatives	level1	88.0399	204.9	beta-Alanine	3.9	107-95-9	It has a role as an inhibitor and an agonist
Organic oxygen compounds	level2	121.064	136	Acetophenone	2.63	98-86-2	Used as a flavoring, solvent, and polymerization catalyst
Organic oxygen compounds	level1	179.0552	174.8	Galactose	3.83	10257-28-0	used in trials studying the treatment and diagnosis of Hepatitis C, Hepatic Cancer, Wilsons Disease, Diabetic Macular Oedema, and Focal Segmental Glomerulosclerosis
Organic oxygen compounds	level2	245.0935	49	Ethanone, 1-(3,4-dihydroxyphenyl)-2-(2-ethyl-1H-imidazol-1-yl)-	2.72	1135683-24-7	unknown
Organic oxygen compounds	level2	367.1033	167.9	3-O-Feruloylquinic acid	2.38	62929-69-5	It has a role as a plant metabolite
Organic oxygen compounds	level2	164.0708	156.2	1-(4-Hydroxyphenyl)-2-methylaminoethanone	2.66	21213-89-8	unknown
Organic oxygen compounds	level2	367.1033	145.5	Cnidioside A	2.35	141896-53-9	unknown
Organic oxygen compounds	level2	593.2705	153.4	Curine	2.56	436-05-5	Anti-inflammatory and analgesic
Organic oxygen compounds	level2	308.0972	175	N-Acetylneuraminic acid	2.31	131-48-6	In neural transmission, white blood cell vascular exudation, viral or bacterial infections play a biological role
Organic oxygen compounds	level2	226.0811	227.7	Cyclocytidine	2.66	31698-14-3	It has a role as a prodrug, an antimetabolite and an antineoplastic agent
Organic oxygen compounds	level2	499.296	232	5,6,16-Trihydroxygrayanotox-10-en-3-yl hexopyranoside	2.46		unknown
Organic oxygen compounds	level2	590.2917	280.3	Norbuprenorphine glucuronide	2.62	469887-29-4	unknown
Organic oxygen compounds	level2	663.2889	235.3	ACARBOSE	2.14		unknown
Organic oxygen compounds	level2	570.2968	276.1	isepamicin	2.44	58152-03-7	exerts a long postantibiotic effect
Organic oxygen compounds	level2	513.269	289	Andrographiside	2.25	82209-76-5	unknown
Organoheterocyclic compounds	level2	222.9869	200.7	3-Bromoquinolin-4-amine	2.69	36825-36-2	unknown
Organoheterocyclic compounds	level2	188.0694	157.6	Atrazine desethyl	2.71	6190-65-4	cause developmental toxicity and female reproductive toxicity
Organoheterocyclic compounds	level2	542.3191	121.8	1H-Indazole-4-carboxamide, N-[(1,2-dihydro-6-methyl-2-oxo-4-propyl-3-pyridinyl)methyl]-1-(1-methylethyl)-6-[2-(4-methyl-1-piperazinyl)-4-pyridinyl]-	2.13	1346704-33-3	cause developmental toxicity and female reproductive toxicity
Organoheterocyclic compounds	level1	111.0195	35.6	Uracil	3.91	66-22-8	Pharmaceutical Intermediates
Organoheterocyclic compounds	level1	151.0254	116.5	Xanthine	3.84	69-89-6	It has a role as a Saccharomyces cerevisiae metabolite.
Organoheterocyclic compounds	level1	151.0254	116.5	Oxypurinol	3.67	2465-59-0	It has a role as an xanthine oxidase inhibitor and a drug metabolite.
Organoheterocyclic compounds	level2	415.1377	40.1	[(6R,7R)-7-Hydroxy-7-methyl-8-oxo-3-[(E)-prop-1-enyl]-5,6-dihydro-1H-isochromen-6-yl] 3,6-dihydroxy-4-methoxy-2-methylbenzoate	2.17		unknown
Organoheterocyclic compounds	level2	248.0752	155.1	Ethyl 2-amino-4-phenylthiophene-3-carboxylate	2.65	4815-36-5	unknown
Organoheterocyclic compounds	level1	161.1064	119.2	Tryptamine	3.7	61-54-1	small-molecule inhibitor
Organoheterocyclic compounds	level2	589.2953	284.4	7-Hydroxy-10-(2-hydroxypropan-2-yl)-2,2a,4,7b,13a-pentamethyl-6-oxo-2a,3,4,6,7,7a,7b,8,9,10,11a,12,13,13a-tetradecahydro-2H-oxeto[2',3':1,5]cyclopenta[1,2-h]pyrano[3,2-a]xanthene-3,8-diyl diacetate	2.31		unknown
Organoheterocyclic compounds	level2	424.1572	202.5	4H-[1,2,4]Triazolo[4,3-a][1,4]benzodiazepine-4-acetamide, 6-(4-chlorophenyl)-N-ethyl-8-methoxy-1-methyl-, (4S)-	2.53	1260907-17-2	as a bromodomain-containing protein 4 inhibitor, an antineoplastic agent and an apoptosis inducer
Organoheterocyclic compounds	level2	148.0347	41.7	4-(Trifluoromethyl)pyridine	2.5	3796-24-5	unknown
Organoheterocyclic compounds	level2	470.233	279.7	Nefazodone	2.65	83366-66-9	It has a role as an antidepressant, a serotonergic antagonist, a serotonin uptake inhibitor, an alpha-adrenergic antagonist and an analgesic.
Organoheterocyclic compounds	level2	391.0754	200.6	5H-Thieno[2,3-c]pyran-3-carboxylic acid, 2-[[(benzoylamino)thioxomethyl]amino]-4,7-dihydro-5,5-dimethyl-	2.14	314042-01-8	can competitively inhibit RAS-related proteins in Brain 7 (Rab7)
Organoheterocyclic compounds	level2	333.0829	203.8	2-Hydroxyethylflurazepam	2.6	20971-53-3	unknown
Organoheterocyclic compounds	level2	219.0764	32.5	(S)-5-(4-Hydroxyphenyl)-5-ethylhydantoin	2.66	65567-35-3	unknown
Organoheterocyclic compounds	level1	158.0604	18.5	4-Methylquinolin-2-ol	3.91	607-66-9	unknown
Organoheterocyclic compounds	level2	452.1884	183.7	Doxazosin	2.58	74191-85-8	an antihypertensive agent, an alpha-adrenergic antagonist, an antineoplastic agent, a vasodilator agent and an antihyperplasia drug
Organoheterocyclic compounds	level1	205.0958	157.8	Tryptophan	3.83	73-22-3	unknown
Organoheterocyclic compounds	level2	369.119	166.7	2-[3-(2-Quinolylmethoxy)anilino]benzoic acid	2	105350-26-3	unknown
Organoheterocyclic compounds	level2	393.1745	191.9	Sparfloxacin	2.2	110871-86-8	antibacterial activity
Organoheterocyclic compounds	level2	205.0806	242.4	5,6,7,8-Tetrahydrothieno[2,3-b]quinolin-4-amine	2.65	122914-50-5	unknown
Organoheterocyclic compounds	level2	309.1231	26.3	1H-Pyrrole-3-propanoic acid, 5-[(1,2-dihydro-2-oxo-3H-indol-3-ylidene)methyl]-2,4-dimethyl-	2.58	252916-29-3	used in trials studying the treatment of Lung Cancer, Breast Cancer, Kidney Cancer, Gastric Cancer, and Prostate Cancer
Organoheterocyclic compounds	level2	443.2307	203.3	Rhodamine B cation	2.66	64381-98-2	used as tracer dyes
Organoheterocyclic compounds	level2	390.2097	289	Alfuzosin	2.58	81403-68-1	is indicated for benign prostatic hyperplasia
Organoheterocyclic compounds	level2	448.2193	258.3	Aspergamide B	2.58	863132-99-4	unknown
Organoheterocyclic compounds	level2	274.1395	174.3	5-Heptyl-2,4-dihydro-4-phenyl-3H-1,2,4-triazole-3-thione	2.65	111650-94-3	unknown
Organoheterocyclic compounds	level2	443.2224	204.2	LOVASTATIN	2.16	75330-75-5	an anticholesteremic drug and an antineoplastic agent
Organoheterocyclic compounds	level2	175.0854	57.4	Indole-3-acetamide	2.45	879-37-8	unknown
Organoheterocyclic compounds	level2	198.1225	206.7	1,3-Dimethyl-6-(propylamino)-2,4(1H,3H)-pyrimidinedione	2.64	5770-45-6	unknown
Organoheterocyclic compounds	level2	275.1389	26.4	5-[4-(Isopentyloxy)phenyl]-5-methyl-2,4-imidazolidinedione	2.68	68524-21-0	unknown
Organoheterocyclic compounds	level2	340.1447	152.1	N-(1,3-Diphenyl-1H-pyrazol-5-yl)benzamide	2.72	77746-90-8	unknown
Organoheterocyclic compounds	level2	372.222	259.2	7H-Pyrrolo[3,2-f]quinazoline-1,3-diamine, N3-cyclopropyl-7-[[4-(1-methylethyl)phenyl]methyl]-	2.58	245520-69-8	as an antibacterial agent, a protease-activated receptor-1 antagonist, a cardioprotective agent and an apoptosis inducer
Organoheterocyclic compounds	level1	72.0803	178.4	Pyrrolidine	3.79	123-75-1	unknown
Organoheterocyclic compounds	level2	205.0629	143.2	1-(5-Nitro-1H-indol-3-yl)ethan-1-one	2.57	4771-10-2	unknown
Organoheterocyclic compounds	level2	374.1666	207.6	1H-Indazole-3-carboxamide, 1-(5-fluoropentyl)-N-1-naphthalenyl-	2.6	1445581-91-8	unknown
Organoheterocyclic compounds	level1	130.0644	22.2	Isoquinoline	3.86	119-65-3	unknown
Organoheterocyclic compounds	level2	301.128	105.5	3-(1H-Indol-4-yl)-N-(3-methoxypropyl)-1,2,4-oxadiazole-5-carboxamide	2.31	1010925-99-1	unknown
Organoheterocyclic compounds	level2	315.1645	206.3	Safranine cation	2.52	7006-08-8	It has a role as a fluorochrome and a histological dye.
Organoheterocyclic compounds	level2	535.2699	289.4	Pyropheophorbide-a	2.73	24533-72-0	unknown
Organoheterocyclic compounds	level2	149.0448	41.9	1-Methyl-1H-pyrazolo[3,4-d]pyrimidin-4-ol	2.52	5334-56-5	unknown
Organoheterocyclic compounds	level1	123.0546	28.8	Nicotinamide	3.86	98-92-0	an antioxidant, a neuroprotective agent, an anti-inflammatory agent
Organoheterocyclic compounds	level2	380.1882	153.6	1,3,5-Trihydroxy-2,4-bis(3-methylbut-2-enyl)-10H-acridin-9-one	2.15	28233-35-4	unknown
Organoheterocyclic compounds	level2	156.0646	25.7	5-Isopropyl-3-isoxazolecarboxylic acid	2.65	89776-74-9	unknown
Organoheterocyclic compounds	level2	411.2146	281.4	Risperidone	2.45	106266-06-2	antipsychotic, a dopaminergic antagonist
Organoheterocyclic compounds	level2	531.2745	195	4-(2-Oxo-1,2,3,4-tetrahydroquinolin-6-yl)oxycilostazol	2.22	1796891-27-4	unknown
Organoheterocyclic compounds	level2	331.1609	266.7	[4-[(E)-2-(1H-Indazol-3-yl)vinyl]phenyl]piperazinomethanone	2.52	1000669-72-6	potential antineoplastic activity
Organoheterocyclic compounds	level2	189.0858	203.1	6-(2-Pyridinyl)-1,3,5-triazine-2,4-diamine	2.53	25007-79-8	unknown
Organoheterocyclic compounds	level2	451.123	113.8	[(3R,3'R,4R,6'S,7R)-4,7-Diacetyloxy-5-hydroxy-6',7-dimethyl-6,8-dioxospiro[4H-isochromene-3,2'-oxane]-3'-yl] acetate	2.63	2117753-48-5	unknown
Organoheterocyclic compounds	level2	456.2444	184.2	(3E)-10b-(2-Methylbut-3-en-2-yl)-3-((5-(2-methylbut-3-en-2-yl)-1H-imidazol-4-yl)methylidene)-6,10b,11,11a-tetrahydro-2H-pyrazino[1',2':1,5]pyrrolo[2,3-b]indole-1,4(3H,5aH)-dione	2.6	871982-52-4	unknown
Organoheterocyclic compounds	level2	345.1749	205	10H-Phenoxazine-10-butanamine, 2-chloro-N,N-diethyl-	2.25	201788-90-1	unknown
Phenylpropanoids and polyketides	level2	593.1485	54.1	Oroxin B	2.67	114482-86-9	pharmaceutical intermediate
Phenylpropanoids and polyketides	level2	533.1278	23.3	5,7-Dihydroxy-2-(4-hydroxyphenyl)-6,8-bis(3,4,5-trihydroxyoxan-2-yl)chromen-4-one	2.26	1236257-31-0	unknown
Phenylpropanoids and polyketides	level2	609.143	39.8	2-Mercaptobenzothiazole	2.18	26544-34-3	It has a role as an EC 3.2.1.18 (exo-alpha-sialidase) inhibitor
Phenylpropanoids and polyketides	level2	475.1218	54.6	Cirsimarin	2.15	13020-19-4	It has an effective anti-fat effect
Phenylpropanoids and polyketides	level2	255.0637	54.7	7,2'-Dihydroxyflavone	2.66	77298-66-9	unknown
Phenylpropanoids and polyketides	level2	477.1017	39.5	Ethylparaben	2.54	20486-34-4	unknown
Phenylpropanoids and polyketides	level2	461.1067	55	Flavone base + 3O, 1MeO, C-Hex	2.71	6980-25-2	unknown
Phenylpropanoids and polyketides	level1	565.1526	101	Apiin	3.83	26544-34-3	It has a role as an EC 3.2.1.18 (exo-alpha-sialidase) inhibitor
Phenylpropanoids and polyketides	level2	415.1012	54.1	Puerarin	2.68	3681-99-0	an autophagy inducer, a cardioprotective agent, an antioxidant, an anti-inflammatory agent, an antipyretic and a ferroptosis inhibitor
Phenylpropanoids and polyketides	level2	405.1171	37.7	2,3,4',5-Tetrahydroxystilbene 2-glucoside	2.67	82373-94-2	It has a role as an antioxidant, a cyclooxygenase 2 inhibitor, an anti-inflammatory agent, a cardioprotective agent, a platelet aggregation inhibitor and an apoptosis inhibitor
Phenylpropanoids and polyketides	level2	623.156	54.1	5-Hydroxy-2-(4-hydroxyphenyl)-7-methoxy-4-oxo-4H-chromen-3-yl 2-O-.beta.-D-galactopyranosyl-.beta.-D-glucopyranoside	2.1		unknown
Phenylpropanoids and polyketides	level2	435.1263	115.8	Coatline A	2.6	87441-88-1	unknown
Phenylpropanoids and polyketides	level2	463.1211	39.7	4-(3,4-Dihydroxyphenyl)-7-methoxy-2-oxo-2H-chromen-5-yl .beta.-D-glucopyranoside	2.66	116310-58-8	unknown
Phenylpropanoids and polyketides	level2	469.1072	54.7	Trifolirhizin	2.65	6807-83-6	antifungal effect
Phenylpropanoids and polyketides	level2	279.0371	146.1	(E)-1-[4-4-Methoxyphenyl]-2-(3,5-dichlorophenyl)ethene	2.53	688348-37-0	unknown
Phenylpropanoids and polyketides	level1	255.0638	24	4',5-Dihydroxyflavone	3.79	6665-67-4	LOX-1/α-glucosidase inhibitor
Phenylpropanoids and polyketides	level2	305.0655	79.9	(-)-Epigallocatechin	2.64	970-74-1	It has a role as an antioxidant
Phenylpropanoids and polyketides	level1	447.1259	54.7	Glycitin	3.86	40246-10-4	It has a role as a plant metabolite
Phenylpropanoids and polyketides	level1	253.0494	24	7,8-Dihydroxyflavone	3.9	38183-03-8	it has shown efficacy against several diseases of the nervous system, including Alzheimer's, Parkinson's, and Huntington's.
Phenylpropanoids and polyketides	level2	509.2093	192.8	(7'R,8'R)-4,7'-Epoxy-3'-methoxy-4',5,9,9'-lignanetetrol_9'-glucoside	2.31		unknown
Phenylpropanoids and polyketides	level2	265.1255	153.8	Benzenepropanoic acid, 4-[2-(2-methylphenyl)ethynyl]-	2.55	1082058-99-8	unknown
Phenylpropanoids and polyketides	level2	641.3328	254.6	N1,N5,N10-Tris-trans-p-coumaroylspermine	2.41		unknown
Phenylpropanoids and polyketides	level1	273.0742	74.5	(±)-Naringenin	3.65	67604-48-2|480-41-1	unknown
Phenylpropanoids and polyketides	level2	283.0599	23.9	Wogonin	2.67	632-85-9	It has a role as a cyclooxygenase 2 inhibitor, an antineoplastic agent, an angiogenesis inhibitor
Phenylpropanoids and polyketides	level2	285.0742	54.8	5,6-Dihydroxy-7-methoxyflavone	2.66	29550-13-8	neuroprotective agents, anti-inflammatory
Phenylpropanoids and polyketides	level2	431.0963	60.7	Apigenin-7-O-glucoside	2.75	578-74-5	It has a role as a non-steroidal anti-inflammatory drug, a metabolite and an antibacterial agent.
Phenylpropanoids and polyketides	level2	449.1066	149.2	Hovetrichoside C	2.64	210050-28-5	unknown
Phenylpropanoids and polyketides	level2	547.1437	31.6	5,7-Dihydroxy-2-phenyl-6-[3,4,5-trihydroxy-6-(hydroxymethyl)oxan-2-yl]-8-(3,4,5-trihydroxyoxan-2-yl)chromen-4-one	2.27	1214688-92-2	unknown
Phenylpropanoids and polyketides	level1	489.1366	31.7	6''-O-Acetylglycitin	3.84	134859-96-4	unknown
Phenylpropanoids and polyketides	level2	271.0587	110	5,7,2'-Trihydroxyflavone	2.66	73046-40-9	unknown
Others	level3.1	476.2764	125.2	1-(linoleoyl)-sn-glycero-3-phosphoethanolamine	1.83		unknown
Others	level2	491.1171	58.8	Oenin	2.67		unknown
Others	level3.1	317.1453	203	Imidazolone A	1.83		unknown
Others	level3.1	519.1121	44.1	Eujambolin	1.75		unknown
Others	level3.2	331.1955	172.9	M331T173	1.71		unknown
Others	level2	851.4344	259.2	(2S,3R)-3-((2'R,3S,4b'R,7'S,10a'R)-7'-((2-O-(6-Deoxy-.alpha.-L-mannopyranosyl)-.beta.-D-glucopyranosyl)oxy)-4b',8',8',10a'-tetramethyl-5-oxotetradecahydro-2'H-spiro[furan-3,1'-phenanthren]-2'-yl)-1-((2R)-4-methyl-5-oxo-2,5-dihydrofuran-2-yl)butan-2-yl acetate	2.28		unknown
Others	level3.1	481.1788	205	1-(glutathion-S-yl)-N-hydroxy-omega-(methylsulfanyl)heptan-1-imine	1.75		unknown
Others	level3.1	245.092	87.8	(indol-3-yl)acetyl-alanine	1.64		unknown
Others	level3.2	458.2582	183.9	M458T184	1.8		unknown
Others	level2	273.1192	206.9	Gln-Gln	2.63	54419-93-1	It has a role as a Mycoplasma genitalium metabolite
Others	level2	334.1778	253	4'-Cyano[1,1'-biphenyl]-4-yl 4-ethylcyclohexanecarboxylate	2.15	67284-56-4	unknown
Others	level3.1	242.0787	227.2	gamma-Glutamyl-beta-cyanoalanine	1.65		unknown
Others	level2	579.2887	151.7	(4E,10E)-13,21-Dihydroxy-8,14,19-trimethoxy-4,10,12,16-tetramethyl-3,20,22-trioxo-2-azabicyclo[16.3.1]docosa-1(21),4,10,18-tetraen-9-yl carbamate	2.27	1467661-98-8	unknown
Others	level3.2	343.1957	185.4	M343T185	1.82		unknown
Others	level2	303.1757	254.8	Arg-Gln	2.62	2483-17-2	It has a role as a metabolite.
Others	level2	597.2996	151.7	LPI(18:2)	2.64		unknown
Others	level2	595.2862	152.2	D-myo-Inositol, 1-[2-hydroxy-3-[(1-oxo-9,12-octadecadienyl)oxy]propyl hydrogen phosphate], [S-(Z,Z)]-	2.64	149056-39-3	unknown
Others	level3.1	516.2254	162.2	Alpha-Trisaccharide	1.83		unknown
Others	level2	404.2119	264.9	N-[(3s,5s,7s)-Adamantan-1-yl]-1-(4-fluorobenzyl)-1H-indazole-3-carboxamide	2.2		unknown

## Discussion

The genus *Streptomyces* is one of the most prolific producers of structurally diverse and biologically valuable natural products in the microbial world. These secondary metabolites possess a wide spectrum of biological activities with extensive applications in clinical medicine, environmental governance, food industry, and agricultural production, including antibacterial, antifungal, antihypertensive, antiviral, antitumor, immunosuppressive, and insecticidal functions (Watve et al., 2001). For instance, *S. termitum* N-15 exhibits remarkable antibacterial activity against multiple aquatic fish pathogens (Peng et al., 2022); *Streptomyces* sp. CA-297274 produces glycinocins E-H, which exert potent inhibitory activity against *Zymoseptoria tritici*, the causative agent of wheat *Septoria tritici* blotch (Fernández-Pastor et al., 2025); marine-derived *Streptomyces* sp. VITSDK1 synthesizes furan-2-yl acetate (C_6_H_6_O_3_), which effectively inhibits fish nodavirus replication in cell lines; and *Streptomyces* sp. AJ8 also displays significant antiviral activity (Suthindhiran et al., 2011). Moreover, numerous *Streptomyces* strains with outstanding antibacterial and anticancer activities have been isolated and characterized from extreme environments such as deep sea, deserts, volcanic areas, and cold regions (Jenifer et al., 2015). In line with previous studies, our research isolated eight *Streptomyces* strains with distinct antagonistic activity against *C. haemulonii* from soil samples (**Figure 1**), further verifying the potential of environmental *Streptomyces* as a potential antifungal resource.

To further explore the antifungal potential and secondary metabolite profile, strain NC-SA6 with the strongest antagonistic activity was selected for in-depth taxonomic identification and fermentation condition optimization. Compared with the *16S rRNA* gene, the five housekeeping genes (*atpD*, *gyrB*, *recA*, *rpoB*, *trpB*) feature higher sequence divergence, making MLSA based on these genes a reliable molecular marker for accurate species-level discrimination of *Streptomyces* (Rong and Huang, 2010). As shown in **Figure 2A**, strain NC-SA6 clustered tightly with *S. anandii* strains with 82% bootstrap support, indicating its close phylogenetic affinity. The physiological and biochemical characteristics are also listed in **Table 4**. Whole-genome comparative analysis further confirmed the taxonomic status: the ANI and dDDH values between NC-SA6 and reference *S. anandii* strains exceeded 97% and 74%, respectively, both surpassing the standard species delineation thresholds, definitively classifying the strain as *S. anandii* (**Figure 2B**; **Table 3**). At present, only a few literature reports focus on *S. anandii*, all of which analyze the activity of a certain class or type of metabolite (Kim and Kwak, 2025; Zhang et al., 2016; Balitz et al., 1981; Zhang et al., 2017; Batra and Bajaj, 1966). Our project integrates genome and metabolome analyses, and investigates its antimicrobial spectrum, aiming to lay a foundation for the discovery of more active substances and their metabolic pathways in the future.

The composition of the fermentation medium and the culture duration are key factors regulating the transcriptional expression of secondary metabolite BGCs and the yield of metabolites. Therefore, we systematically optimized the culture conditions to enhance antifungal metabolite production. The results showed that the maximum antifungal activity against *C. haemulonii* was achieved when cultured in No. 6 medium for 4 days, while no antifungal activity was detected in ISP1, ISP2, or ISP3 media (**Figure 2C**). This result indicates that medium components significantly affect the biosynthesis of active metabolites in *S. anandii* NC-SA6, and the optimized condition was adopted for subsequent functional assays.

Consistent with previous reports on the antimicrobial spectrum of *Streptomyces*, *S. anandii* NC-SA6 exerted broad-spectrum antagonistic activity against pathogenic *Candida* species and Gram-positive bacteria, but no inhibitory activity against the tested Gram-negative bacteria (**Figure 3**). This selective antimicrobial activity may be related to the structural characteristics of target microbial cell walls and the action mechanism of active metabolites.

Whole-genome sequencing and mining revealed that approximately 10% of the *S. anandii* NC-SA6 genome is dedicated to secondary metabolism, highlighting its robust potential for bioactive metabolite biosynthesis. A total of 19 secondary metabolite BGCs were predicted (**Table 6**), including 12 clusters with high homology to known functional BGCs and 7 novel cryptic clusters. Notably, nearly half of the total BGC length comprises clusters associated with antibacterial and antifungal activities, providing a genetic basis for the strain’s prominent antimicrobial phenotype.

Comparative metabolomics analysis further demonstrated that NC-SA6 is a highly promising source of active metabolites. A total of 15 superclasses of secondary metabolites were identified in the fermentation broth (**Table 8**) with hundreds of significantly differential metabolites between active and inactive broths. And the up-regulated and down-regulated compounds (over 5 times) were listed in **Table 9** and **Table 10**, respectively. Among them, the up-regulated metabolites were considered the main contributors to the antimicrobial activity, among which over 50% were uncharacterized novel metabolites, and nearly 4.34% were known antifungal and antibacterial compounds (**Figure 6C**). Unfortunately, we did not perform complementary reversed-phase analysis which may lead to incomplete coverage of the secondary metabolome. In future research, we will employ two-dimensional analysis to achieve comprehensive coverage of both polar and apolar components of the metabolome. But it indicates that *S. anandii* NC-SA6 is a promising resource for novel antifungal drug discovery.

Numerous previous studies have validated the potential of *Streptomyces* secondary metabolites as novel antimicrobial agents. For example, *Streptomyces* sp. YC69 exhibits dose-dependent antagonistic activity against phytopathogenic fungi and anticancer activity (Bubici et al., 2019); *Streptomyces* sp. SRMA3 displays potent antibacterial and antibiofilm activity against drug-resistant clinical pathogens (Dhandapani et al., 2022); *S. virginiae* W18 and *S. amritsarensis* N1–32 show broad-spectrum antibacterial activity against aquatic pathogens (Hu et al., 2021; Li et al., 2020). In this study, *S. anandii* NC-SA6 was shown to produce a variety of bioactive secondary metabolites with significant antagonistic effects on clinical pathogenic fungi, especially multidrug-resistant *C. haemulonii*. The integrated genome and metabolomic analysis provides solid scientific evidence for the development and utilization of this strain as a biological control and antifungal therapeutic potential resource. Future research will focus on the isolation, purification, and structural identification of key active metabolites, as well as the in-depth elucidation of their antifungal mechanisms.

## Data Availability

The datasets presented in this study can be found in online repositories. The names of the repository/repositories and accession number(s) can be found in the article/**Supplementary Material.**

## Supplementary material

Supplementary Table 1 The list of antimicrobial from up-regulate Secondary metabolites.

Supplementary Figure 1 Mass spectrogram of antimicrobial from up-regulate Secondary metabolites.
