# Supplementary material for: The anti-Candida haemulonii activity and bioactive metabolites of Streptomyces anandii NC-SA6
Source: Front Cell Infect Microbiol. 2026 Jun 30;16:1851966. doi: 10.3389/fcimb.2026.1851966 (PMC13364919; doi:10.3389/fcimb.2026.1851966)
Supplement: Supplementary Table 1 — The list of antimicrobial from up-regulate Secondary metabolites. [file Table1.docx]

Table 1 The list of the strains in this study

| **Strain** | **Cultivation temperature** | **Reference or Source** |
| --- | --- | --- |
| *Streptomyces anandii* NC-SA6 | 28 ℃ | This study |
| *Candida krusei* ATCC6258 | 28 ℃ | Laboratory collection |
| *Candida haemulonii* 190070 | 28 ℃ | Laboratory collection |
| *Candida auris* BJCA001 | 28 ℃ | Laboratory collection |
| *Candida albicans* SC5314 | 28 ℃ | Laboratory collection |
| *Escherichia coli* ATCC25926 | 37 ℃ | Laboratory collection |
| *Shigella flexneri* ATCC12022 | 37 ℃ | Laboratory collection |
| *Pseudomonas aeruginosa* PA14 | 37 ℃ | Laboratory collection |
| *Burkholderia cenocepacia* H111 | 37 ℃ | Laboratory collection |
| *Bacillus velezensis* IF-1 | 28 ℃ | Laboratory collection |
| *Bacillus cereus* ATCC14579 | 30 ℃ | Laboratory collection |
| *Staphylococcus aureus* ATCC29213 | 37 ℃ | Laboratory collection |

Table 2 The list of the primers in this study

| **Primers** | **Sequence(5’-3’)** | **Tm (℃)** | **Length (bp)** |
| --- | --- | --- | --- |
| 16S-F | AGAGTTTGATCCTGGCTCAG | 55 | 1400 |
| 16S-R | CTACGGCTACCTTGTTACGA | 55 | 1400 |
| atpD-F | ATGACCACCACTGTTGAGACCGCGA | 70 | 1400 |
| atpD-R | TCAGGAGACGCCCAGCTCCTTGGCG | 70 | 1400 |
| gyrB-F | GTGGCCGATTCCGGCAACCCCAACG | 60 | 2100 |
| gyrB-R | TCAGATGTCGAGGAAGCGGACGTCC | 60 | 2100 |
| recA-F | ATGGCAGGAACCGACCGCGAGAAGG | 65 | 1131 |
| recA-R | TCAGCTCTTGGCCGCCGCGGCCTTG | 65 | 1131 |
| rpoB-F | TTGGCCGCCTCGCGCAATGCCTCGA | 67 | 3486 |
| rpoB-R | TCAGACCTCTTCGACGCTGCTCGGC | 67 | 3486 |
| 2trpB-F | AGGACCTGAACCACACCGGCT | 65 | 800 |
| 2trpB-R | TCGATGGCCGGGATGATGC | 65 | 800 |

Table 3 ANIm and dDDH values among 7 pairs of type *Streptomyces* species

| **Query genome** | **Reference genome** | **dDDH** | **Prob. DDH >= 70%** | **ANIm** |
| --- | --- | --- | --- | --- |
| S. anandii NC-SA6 | S.anandii_NPDC014745 | 74.3 | 84.88 | 97.23 |
| S. anandii NC-SA6 | S.anandii_NPDC020611 | 89.3 | 95.57 | 98.85 |
| S. anandii NC-SA6 | S.anandii_NPDC059492 | 96.1 | 97.47 | 99.6 |
| S. anandii NC-SA6 | S.anandii_NPDC059497 | 96.2 | 97.47 | 99.61 |
| S. anandii NC-SA6 | S.anandii_NPDC059500 | 95.9 | 97.42 | 99.58 |
| S. anandii NC-SA6 | S.geysiriensis_S19 | 26.9 | 0.02 | 86.77 |
| S. anandii NC-SA6 | S.minutiscleroticus_JCM4790 | 25.9 | 0.01 | 86.51 |

Note: dDDH ≥70% and ANIm ≥95-96%，this indicates the two genomes belong to the same species.

Table 4 Physiological and biochemical characteristics of *S. anandii* NC-SA6

| **Biolog** | **Result** | **Biolog** | **Result** | **Biolog** | **Result** |
| --- | --- | --- | --- | --- | --- |
| Cell morphology |  | ribose | - | PH 7.0-9.0 | + |
| Gram stain |  | arabinose | - | PH 10.0-13.0 | - |
| 0%-5% Nacl Salt tolerance test | ++ | maltose | - | phaseomannite | ++ |
| 6%-13%Nacl Salt tolerance test | - | L-Asparagine | - | D-Trehalose | + |
| Temperature 4-10℃ | - | L-lysine | - | D-fructose | ++ |
| Temperature 16-45℃ | + | L-Cysteine | ++ | D-raffinose | ++ |
| Nitrate reduction | + | L-threonine | ++ | D-sorbitol | + |
| Gelatin liquefaction | - | L-valine | ++ | D-galactose | - |
| Milk coagulation and peptones | + | L-Methionine | ++ | D-mannitol | - |
| Hydrolysis test of aesculin | + | L-serine | ++ | D-glucose | ++ |
| H2S production | + | D-arginine | - | rhamnose | + |
| Melanin production | + | creatine | ++ | xylose | - |
| Contact enzyme test | - | PH 4.0-6.0 | - | sucrose | - |

Note: ++:grown well; +: growth or positive; -: ungrown or negative

Table 5 The values of mean±SD (figure 2C) at different conditions

| Medium/Time(day) | **2D** | **3D** | **4D** | **5D** | **6D** | **7D** |
| --- | --- | --- | --- | --- | --- | --- |
| **1** | 0 | 0 | 0 | 0 | 0 | 0 |
| **2** | 0 | 0 | 0 | 0 | 0 | 0 |
| **3** | 0 | 0 | 0 | 0 | 0 | 0 |
| **4** | 0 | 0.65±0.15 | 0.8±0.1 | 1.15±0.05 | 1.3±0.1 | 1.2±0.1 |
| **6** | 0 | 1.5±0.1 | 1.7±0.1 | 1.5±0.1 | 1.45±0.05 | 1.3±0.1 |
| **7** | 0 | 1.3±0.1 | 1.35±0.05 | 1.25±0.05 | 1.35±0.05 | 1.3±0.1 |

Table 6 The list of secondary metabolism clusters prediction of *S. anandii* NC-SA6

| **Compound** | **Synthetase Type** | **Core genes** | **Size(Kb)** | **Most similar known cluster** | **Similarity** | **Bioactivity** | **BGC coordinates** | **MIBiG accession** |
| --- | --- | --- | --- | --- | --- | --- | --- | --- |
| melanin | melanin | iolS_1,adhA | 8.7 | melanin | 57% | antimicrobial | 1 - 6,497 nt | BGC0000908 |
| herboxidiene | T3PKS | qorA_1,ahpF,gabD1,acdA_1,def_1,rocD_1 | 41.1 | herboxidiene | 7% | herbicidal, anti-cholesterol | 3,785 - 90,616 nt | BGC0001065 |
| ectoine | ectoine | mltF_1,ppaT,ectB,ectC,cefD | 10.4 | ectoine | 100% | skin protection | 1 - 3,366 nt | BGC0000853 |
| melanin | melanin |  | 10.5 | melanin | 60% | antimicrobial | 1 - 6,625 nt | BGC0000909 |
| desferrioxamin B / desferrioxamine E | siderophore | ddc_2,iucD_2,iucB,iucC | 11.2 | desferrioxamin B / desferrioxamine E | 83% | antibacterial | 3,024,895 - 3,054,682 nt | BGC0000940 |
| spore pigment | lanthipeptide-class-iii,T2PKS | gpgS,lacC_2,nphA2,mnmC_2,mmcR,tcmI_1,dntB_1,cugP_3,ramS,ramC | 70 | spore pigment | 83% | other | 1 - 11,118 nt | BGC0000271 |
| phosphonoglycans | NRPS | caeB_2,dhbF,tycC_2,ddl_1,asnB_2,thadh_2,hprA | 49 | phosphonoglycans | 3% | Promote lipid metabolism | 1 - 70,887 nt | BGC0000806 |
| xantholipin | RRE-containing,T2PKS | pccB_1,lgrE,baiA,tcmI_2,fabG_6,garR_1,dntB_2,asnO_2,dntB_3,adoK_2,acs,mycG_1 | 72.5 | xantholipin | 44% | Cytotoxic, antibacterial | 1 - 52,255 nt | BGC0000279 |
| albaflavenone | terpene | def_2,cyc1 | 20.5 | albaflavenone | 100% | novel antibiotic | 5,671,016 - 5,692,101 nt | BGC0000660 |
| ficellomycin | siderophore | dat_2,iucA | 10.7 | ficellomycin | 3% | antibiotic | 11,780 - 55,969 nt | BGC0001593 |
| geosmin | terpene | cyc2,pepE,treZ | 21 | geosmin | 100% | other | 6,656,219 - 6,678,399 nt | BGC0001181 |
| ECO-02301 | T1PKS,NRPS-like | ggt_1,map_2,argD_2,fgd_5,dltA_2,pikAV_1,elmGT,fadA_4-10,pikAI,eryA,fabD_2,strL,novW,cugP_4,strE_2,pikAV_2,lcfB_6,npt,pccB_3 | 196.2 | ECO-02301 | 85% | Novel antifungal agent | 1 - 161,719 nt | BGC0000052 |
| tetronasin | T1PKS | yecD_1,fabH_2,Vejahgd,bioF_2,cmoM_3,dasA_3 | 44.5 | tetronasin | 3% | antibiotic | 1 - 169,690 nt | BGC0000163 |
| hopene | terpene | galE_3,glgC_1,aroB_2,crtB_1,hpnE,shc,moaA_2,dxs_2,pigE | 25.9 | hopene | 92% | other | 7,506,017 - 7,532,758 nt | BGC0000663 |
| informatipeptin | RiPP-like | lagD | 7.8 | informatipeptin | 42% | other | 8,165,944 - 8,193,261 nt | BGC0000518 |
| isorenieratene | RiPP-like,terpene | fabH_3,ilvB,crtB_2,crtI,crtY,ubiE | 25.5 | isorenieratene | 75% | antioxidant | 1 - 9,777 nt | BGC0001456 |
| cyphomycin | butyrolactone | hldD_2,hsaA,menC | 16 | cyphomycin | 5% | antimicrobial | 423,393 - 622,305 nt | BGC0001877 |
| rabelomycin / dehydrorabelomycin / fluostatin F, G, H | lanthipeptide-class-iv | iscS_3,maa,yfeW_3,pknD_22,cpo_2,nicF | 22.4 | rabelomycin / dehydrorabelomycin / fluostatin F, G, H | 3% | other | 1 - 66,898 nt | BGC0000223 |
| colicin V | terpene | uppS | 22.6 | colicin V | 1% | activate protein kinase C | 15,749 - 19,808 nt | BGC0001555 |

Table 7 The parameters of OPLS-DA model validation

| **Type** | **A** | **N** | **R2X(cum)** | **R2Y(cum)** | **Q2(cum)** |
| --- | --- | --- | --- | --- | --- |
| OPLS-DA | 1+1+0 | 6 | 0.731 | 1.0 | 0.964 |

Table 8 The list of total secondary metabolites superclass of *S. anandii* NC-SA6

| **Superclass** | **Freq** | **Percent** |
| --- | --- | --- |
| Organoheterocyclic compounds | 430 | 25.25% |
| Organic acids and derivatives | 331 | 19.44% |
| Lipids and lipid-like molecules | 243 | 14.27% |
| Benzenoids | 203 | 11.92% |
| Phenylpropanoids and polyketides | 128 | 7.52% |
| Organic oxygen compounds | 121 | 7.11% |
| Others | 121 | 7.11% |
| Organic nitrogen compounds | 41 | 2.41% |
| Nucleosides, nucleotides, and analogues | 38 | 2.23% |
| Alkaloids and derivatives | 21 | 1.23% |
| Lignans, neolignans and related compounds | 17 | 1% |
| Organosulfur compounds | 6 | 0.35% |
| Organic 1,3-dipolar compounds | 1 | 0.06% |
| Organohalogen compounds | 1 | 0.06% |
| Organophosphorus compounds | 1 | 0.06% |

Table 9 The list of up-regulate Secondary metabolites (Fold change≥5)

| **Super.Class** | **Level** | **m/z** | **rt** | **MS2 name** | **MS2 score** | **CAS** | **Description** |
| --- | --- | --- | --- | --- | --- | --- | --- |
| Benzenoids | level2 | 257.1117 | 74.3 | N-(2,6-Dimethylphenyl)-N'-phenylthiourea | 2.58 | 25347-93-7 | Inhibitors of phenol oxidase |
| Benzenoids | level2 | 287.0987 | 118.8 | N-(3-Methoxyphenyl)-2-methyl-3-nitrobenzamide | 2.44 | 123400-20-4 | unknown |
| Benzenoids | level2 | 201.0857 | 188.5 | Diphenylmethylphosphine | 2.51 | 1486-28-8 | unknown |
| Benzenoids | level2 | 273.083 | 116.6 | 2-Hydroxy-N-(4-methylphenyl)-5-nitrobenzamide | 2.46 | 68507-96-0 | unknown |
| Benzenoids | level2 | 233.0908 | 117.3 | N-(1,3-Dioxolan-2-ylmethoxy)benzimidoyl cyanide | 2.5 | 74782-23-3 | unknown |
| Benzenoids | level2 | 332.0722 | 48.8 | 4-{2-[(5-Chloro-2-methoxybenzoyl)amino]ethyl}benzoic acid | 2.18 | 54870-28-9 | antidiabetic agents with hypoglycemic activity |
| Benzenoids | level2 | 344.1785 | 293.7 | Clemastine | 2.21 | 15686-51-8 | unknown |
| Benzenoids | level2 | 223.1428 | 121.1 | Neostigmine cation | 2.65 | 59-99-4 | A cholinesterase inhibitor |
| Benzenoids | level2 | 281.0529 | 76.7 | Niflumic acid | 2.29 | 4394-00-7 | an analgesic and anti-inflammatory agent |
| Lipids and lipid-like molecules | level2 | 855.4709 | 99.8 | Ophiopogonin D | 2.2 | 945619-74-9 | Related research of inflammation and cardiovascular diseases |
| Lipids and lipid-like molecules | level2 | 147.0656 | 31 | (2R)-2,4-Dihydroxy-3,3-dimethylbutanoic acid | 2.67 | 1112-33-0 | unknown |
| Lipids and lipid-like molecules | level2 | 411.2704 | 151.8 | 3-Methoxylimaprost | 2.07 | 1224443-96-2 | 3-methoxylimaprost is a prostanoid |
| Lipids and lipid-like molecules | level2 | 445.2568 | 147.5 | Bufotalin | 2.35 | 471-95-4 | anti-tumor |
| Lipids and lipid-like molecules | level2 | 553.2778 | 260.6 | Euphorbiasteroid | 2.27 | 28649-59-4 | Antibacterial |
| Nucleosides, nucleotides, and analogues | level1 | 312.1287 | 102.3 | N2,N2-Dimethylguanosine | 3.86 | 2140-67-2 | It has a role as a human metabolite |
| Nucleosides, nucleotides, and analogues | level1 | 336.1649 | 29 | Isopentenyladenosine | 3.83 | 7724-76-7 | Pharmaceutical Intermediates |
| Organic acids and derivatives | level2 | 187.154 | 162.2 | 3-(4-Methyl-1-piperazinyl)propanohydrazide | 2.52 | 24636-93-9 | unknown |
| Organic acids and derivatives | level2 | 301.2006 | 100 | Arg-Lys | 2.67 | 40968-46-5 | unknown |
| Organic acids and derivatives | level2 | 157.0962 | 38.5 | N-Acetyl-L-Prolinamide | 2.63 | 16395-58-7 | Pharmaceutical Intermediates |
| Organic acids and derivatives | level2 | 240.0951 | 188.6 | (2S)-2-Amino-3-(2,4-diketo-6,7-dihydro-5H-cyclopenta[d]pyrimidin-1-yl)propionic acid | 2.36 | 389888-02-2 | unknown |
| Organic acids and derivatives | level1 | 141.0663 | 198.1 | Ectoine | 3.88 | 96702-03-3 | Chinese herbal medicinal ingredient |
| Organic acids and derivatives | level1 | 172.0971 | 103 | Acetylleucine | 3.83 | 1188-21-2 | Pharmaceutical Intermediates |
| Organic acids and derivatives | level2 | 160.0958 | 153.7 | Betonicine | 2.64 | 515-25-3 | It has a role as a plant metabolite |
| Organic acids and derivatives | level1 | 218.1122 | 188.1 | N-Acetylcitrulline | 3.87 | 33965-42-3 | unknown |
| Organic acids and derivatives | level1 | 427.2998 | 141.2 | Leupeptin (hemisulfate) | 3.78 | 103476-89-7 | protease inhibitor |
| Organic acids and derivatives | level2 | 445.3106 | 145.3 | 2-Stearyl_citrate | 2.21 |  | unknown |
| Organic acids and derivatives | level2 | 269.1118 | 34.7 | Serinyl-Tyrosine | 2.21 |  | unknown |
| Organic acids and derivatives | level1 | 217.1281 | 218 | N-Acetyl-arginine | 3.86 | 155-84-0 | It has a role as a human metabolite |
| Organic acids and derivatives | level2 | 205.117 | 221.2 | 3-Methylpentane-2,4-diyl dicarbamate | 2.57 | 5667-70-9 | unknown |
| Organic acids and derivatives | level1 | 208.0956 | 89.8 | N-Acetylphenylalanine | 3.85 | 2018-61-3 | anti-inflammatory agent |
| Organic acids and derivatives | level2 | 132.1123 | 232 | 1-(4-Aminobutyl)urea | 2.61 | 6851-51-0 | unknown |
| Organic acids and derivatives | level2 | 293.0729 | 49.5 | Etrimfos | 2.56 | 38260-54-7 | Pesticides and insecticides |
| Organic acids and derivatives | level2 | 293.148 | 122.3 | N2-(1-Oxo-4-phenylbutyl)-L-glutamine | 2.27 | 491851-62-8 | Phenylbutyrylglutamine is a glutamine derivative |
| Organic acids and derivatives | level1 | 265.1166 | 130.5 | Phenylacetylglutamine | 3.82 | 28047-15-6 | It has a role as a human metabolite |
| Organic acids and derivatives | level2 | 211.1428 | 147.5 | Cyclo(leucylprolyl) | 2.64 | 5654-86-4 | unknown |
| Organic acids and derivatives | level2 | 160.1071 | 199.6 | Creatine, ethyl ester | 2.62 | 15366-29-7 | unknown |
| Organic acids and derivatives | level1 | 230.185 | 201.5 | N1,N8-Diacetylspermidine | 3.86 | 82414-35-5 | unknown |
| Organic acids and derivatives | level1 | 247.1064 | 105.6 | N-Acetyltryptophan | 3.9 | 1218-34-4 | It has a role as a metabolite |
| Organic nitrogen compounds | level2 | 201.1024 | 19.7 | Pesticide4_Dinotefuran_C7H14N4O3_Guanidine, N-methyl-N''-nitro-N'-[(tetrahydro-3-furanyl)methyl]- | 2.55 | 165252-70-0 | Pesticides and insecticides |
| Organic nitrogen compounds | level1 | 142.0966 | 219.7 | Histidinol | 3.72 | 4836-52-6 | glycylpeptide N-tetradecanoyltransferase inhibitor |
| Organic oxygen compounds | level2 | 396.2107 | 207.7 | Spiperone | 2.49 | 749-02-0 | Small molecule inhibitors |
| Organic oxygen compounds | level2 | 290.21 | 20.6 | 1-Heptanone, 1-(4-methoxyphenyl)-2-(1-pyrrolidinyl)- | 2.66 | 1801552-04-4 | unknown |
| Organic oxygen compounds | level1 | 255.0958 | 221.4 | Nicotinamide riboside (NR) | 3.8 | 1341-23-7 | anti-aging |
| Organoheterocyclic compounds | level2 | 285.0824 | 183.4 | Nolatrexed | 2.64 | 147149-76-6 | anti-tumor |
| Organoheterocyclic compounds | level2 | 289.0318 | 48.3 | 5-Chloro-N-[4-(2-pyridinyl)-1,3-thiazol-2-yl]-2-pyridinamine | 2.69 | 696628-24-7 | unknown |
| Organoheterocyclic compounds | level2 | 207.073 | 185.9 | 5-(2,3-Difluorophenyl)-2-pyridinamine | 2.73 | 875166-91-9 | unknown |
| Organoheterocyclic compounds | level2 | 492.2404 | 129.3 | 6b-Hydroxy-3,14b,14c-trimethyl-5-oxo-2,3,4,5,6b,7,8,8a,9,14,14b,14c,15,16-tetradecahydro-4,16a-epoxy[1]benzoxocino[7',8':6,7]indeno[1,2-b]indol-3-yl acetate | 2.18 |  | unknown |
| Organoheterocyclic compounds | level2 | 409.2897 | 141.5 | 6a,7,10,10a-Tetrahydro-3-[5-(1H-imidazol-1-yl)-1,1-dimethylpentyl]-6,6,9-trimethyl-6H-dibenzo[b,d]pyran-1-ol | 2.56 | 1347588-01-5 | unknown |
| Organoheterocyclic compounds | level2 | 203.1169 | 19.9 | 2-(Piperidin-1-yl)benzo[d]oxazole | 2.67 | 2851-09-4 | unknown |
| Organoheterocyclic compounds | level2 | 251.141 | 292.8 | Methyl (E)-3-[5-[(E)-4-hydroxy-3-methylbut-2-enyl]-1-methylimidazol-4-yl]prop-2-enoate | 2.49 | 2066909-80-4 | unknown |
| Organoheterocyclic compounds | level1 | 155.0091 | 124.7 | Orotic acid | 3.9 | 65-86-1 | unknown |
| Organoheterocyclic compounds | level2 | 201.1123 | 110 | 2-[5-(2-Hydroxypropyl)oxolan-2-yl]propanoic acid | 2.44 | 3688-54-8 | unknown |
| Organoheterocyclic compounds | level1 | 161.07 | 27.2 | SD-169 | 3.77 | 1670-87-7 | treat diabetes |
| Organoheterocyclic compounds | level2 | 151.0606 | 62.8 | 5-(2-Furyl)-4H-1,2,4-triazol-3-amine | 2.66 | 3663-61-4 | unknown |
| Organoheterocyclic compounds | level2 | 310.1143 | 102.4 | 2,6-Bis(2-benzimidazolyl)pyridine | 2.5 | 28020-73-7 | unknown |
| Organoheterocyclic compounds | level2 | 470.1865 | 253.5 | 1-[(4-Chlorophenyl)methyl]-3-[(1,1-dimethylethyl)thio]-.alpha.,.alpha.- dimethyl-5-(1-methylethyl)-1H-indole-2-propanoic acid | 2.33 | 118414-82-7 | Small molecule inhibitors |
| Organoheterocyclic compounds | level2 | 115.0858 | 232.1 | 4,4-Dimethyl-4,5-dihydro-1,3-oxazol-2-amine | 2.6 | 52832-91-4 | unknown |
| Organoheterocyclic compounds | level2 | 256.0801 | 195.9 | 6-Methyl-3-phenylpyrimido[5,4-e][1,2,4]triazine-5,7(6H,8H)-dione | 2.1 | 42285-76-7 | unknown |
| Organoheterocyclic compounds | level2 | 557.267 | 265.3 | Dipiperamide_C | 2.35 |  | unknown |
| Organosulfur compounds | level2 | 184.0556 | 41.2 | 3-(Allylsulfanyl)-6-methyl-1,2,4-triazin-5-ol | 2.56 | 87450-64-4 | unknown |
| Phenylpropanoids and polyketides | level2 | 291.0938 | 34.4 | (2R,3R)-2-(3,4-dihydroxyphenyl)-3,4-dihydro-2H-chromene-3,5,7-triol | 2.53 | 490-46-0 | Small molecule inhibitors, anti-inflammatory |
| Phenylpropanoids and polyketides | level2 | 271.0909 | 49.5 | Alloimperatorin | 2.7 |  | unknown |
| Phenylpropanoids and polyketides | level2 | 212.0905 | 122.3 | Methyldopa | 2.65 | 555-30-6 | hypotensive drugs |
| Others | level2 | 883.5021 | 99.5 | (3.beta.,5.Xi.,9.Xi.)-23-Hydroxy-3-((.beta.-D-xylopyranosyl-(1->3)-6-deoxy-.alpha.-L-mannopyranosyl-(1->2)-.alpha.-L-arabinopyranosyl)oxy)olean-12-en-28-oic acid | 2.27 |  | unknown |
| Others | level3.1 | 459.3263 | 137.8 | N-Eicosapentaenoyl Arginine | 1.69 |  | unknown |
| Others | level3.1 | 441.3159 | 136.1 | N-[1-[[5-(Diaminomethylideneamino)-1-oxopentan-2-yl]amino]-4-methyl-1-oxopentan-2-yl]-4-methyl-2-(propanoylamino)pentanamide | 1.75 |  | unknown |
| Others | level3.2 | 457.3105 | 155.9 | M457T156 | 1.77 |  | unknown |
| Others | level2 | 615.2713 | 251.7 | Norethindrone 2,3,4-tri-O-acetyl-.beta.-D-glucuronide methyl ester | 2.03 |  | unknown |
| Others | level3.1 | 270.1431 | 148 | 1-[2-[[Pyrrolidine-2-carbonyl]amino]acetyl]pyrrolidine-2-carboxylic Acid | 1.73 |  | unknown |


Table 10 The list of down-regulate Secondary metabolites (Fold change≥5)

| **Super.Class** | **level** | **mz** | **rt** | **MS2 name** | **MS2 score** | **CAS** | **Description** |
| --- | --- | --- | --- | --- | --- | --- | --- |
| Alkaloids and derivatives | level2 | 460.1651 | 212.2 | Hydromorphone-3-.beta.-D-glucuronide | 2.13 | 40505-76-8 | unknown |
| Alkaloids and derivatives | level2 | 779.4114 | 271.9 | Vinorelbine | 2.48 | 71486-22-1 | Antitumor drugs |
| Benzenoids | level2 | 432.0994 | 39.3 | Nonanamide, N-[(4-hydroxy-2-iodo-5-methoxyphenyl)methyl]-8-methyl- | 2.09 | 1177195-52-6 | unknown |
| Benzenoids | level2 | 428.2596 | 224.3 | Ranolazine | 2.53 | 95635-55-5 | Small molecule inhibitors |
| Benzenoids | level1 | 269.0445 | 19.7 | Emodin | 3.92 | 518-82-1 | Antitumor drugs |
| Benzenoids | level1 | 431.0969 | 110.2 | Emodin-8-glucoside | 3.65 | 38840-23-2 | Chinese herbal medicinal ingredient |
| Benzenoids | level2 | 436.2141 | 183.5 | Benzoic acid, 4-hydroxy-3-[[2-(4-tricyclo[3.3.1.1(3,7)]dec-1-ylphenoxy)acetyl]amino]-, methyl ester | 2.18 | 934593-90-5 | inhibitors |
| Benzenoids | level1 | 161.1064 | 119.2 | Tolazoline (hydrochloride) | 3.65 | 59-98-3 | Pharmaceutical Intermediates |
| Benzenoids | level2 | 320.1747 | 273.9 | Imidafenacin | 2.21 | 170105-16-5 | Pharmaceutical Intermediates |
| Benzenoids | level2 | 344.0385 | 191.7 | Phenol, 2-[4-[(3,5-dichlorophenyl)amino]-6-methyl-2-pyrimidinyl]- | 2.62 | 381683-04-1 | unknown |
| Benzenoids | level1 | 138.0905 | 136 | Tyramine | 3.74 | 51-67-2 | Pharmaceutical Intermediates |
| Benzenoids | level2 | 397.1459 | 197.9 | Kadethrine | 2.22 | 58769-20-3 | pesticide |
| Benzenoids | level2 | 353.1202 | 201.3 | (3Z,5E)-3,5-Bis(4-methoxybenzylidene)tetrahydro-4H-thiopyran-4-one | 2.25 |  | unknown |
| Benzenoids | level2 | 321.17 | 254.3 | [2-[4-Methyl-2-(2-methylpropanoyloxy)phenyl]oxiran-2-yl]methyl 2-methylpropanoate | 2.73 | 22518-06-5 | Antibacterial |
| Benzenoids | level2 | 357.1764 | 198.1 | [(1S)-1-Benzyl-2-[[5-(3-methyl-2H-indazol-5-yl)-3-pyridyl]oxy]ethyl]amine | 2.55 | 552325-73-2 | Small molecule inhibitors, Signal transduction pathway kinase inhibitors |
| Benzenoids | level2 | 268.1271 | 181.2 | 4-tert-Butyl-2,6-dimethyl-3,5-dinitroaniline | 2.39 | 107342-55-2 | unknown |
| Benzenoids | level2 | 286.0774 | 54.7 | (2,6-Dichlorophenyl)(3,5-dimethylpiperidin-1-yl)methanone | 2.65 | 346724-91-2 | unknown |
| Benzenoids | level2 | 249.1435 | 269 | N-Cycloheptyl-N'-phenylthiourea | 2.59 | 102936-60-7 | unknown |
| Benzenoids | level2 | 144.08 | 118.7 | 2-Aminonaphthalene | 2.65 | 91-59-8 | unknown |
| Benzenoids | level2 | 319.1594 | 203.8 | Fluvoxamine | 2.45 | 54739-18-3 | Medication for the nervous system |
| Benzenoids | level1 | 137.0237 | 86.4 | 4-Hydroxybenzoic acid | 3.81 | 99-96-7 | Small molecule inhibitors |
| Benzenoids | level1 | 137.0237 | 86.4 | 3-Hydroxybenzoic acid | 3.73 | 36320 | Novel selective and partial agonists of 5-HT3 receptors |
| Lignans, neolignans and related compounds | level2 | 481.108 | 33.1 | 2,3-Dehydrosilybin | 2.55 | 25166-14-7 | used in the treatment of hepatitis; liver cirrhosis; and chemical and drug induced liver injury |
| Lignans, neolignans and related compounds | level2 | 419.2119 | 266.4 | 2-(4-Allyl-2,6-dimethoxyphenoxy)-1-(3,4,5-trimethoxyphenyl)-1-propanol | 2.12 | 41551-58-0 | unknown |
| Lignans, neolignans and related compounds | level2 | 362.2003 | 270.9 | 4-[5-(4-hydroxy-3-methoxyphenyl)-3,4-dimethyloxolan-2-yl]-2-methoxyphenol | 2.07 | 112652-46-7 | unknown |
| Lipids and lipid-like molecules | level1 | 214.0476 | 230.2 | Glycerophosphoethanolamine | 3.81 | 1190-00-7 | Active candidate |
| Lipids and lipid-like molecules | level2 | 477.2681 | 152.6 | Betamethasone-17-valerate | 2.2 |  | unknown |
| Lipids and lipid-like molecules | level2 | 543.1235 | 38.2 | (1aR,5S)-5b-((Benzoyloxy)methyl)-5a-(.beta.-D-glucopyranosyloxy)-5-methylhexahydro-2H-2,5-methano-3,4-dioxacyclobuta[cd]pentalene-2-sulfonic acid | 2.09 |  | unknown |
| Lipids and lipid-like molecules | level2 | 476.2745 | 130.8 | LPE(18:3) | 2.64 |  | unknown |
| Lipids and lipid-like molecules | level1 | 258.1083 | 226.9 | Glycerophosphocholine | 3.86 | 28319-77-9 | as a parasympatholytic, a neuroprotective agent |
| Lipids and lipid-like molecules | level2 | 311.2214 | 27.7 | (9Z,12E)-15,16-Dihydroxyoctadeca-9,12-dienoic acid | 2.66 |  | unknown |
| Lipids and lipid-like molecules | level2 | 478.2905 | 124.7 | PE(18:2(9Z,12Z)/0:0) | 2.23 | 85046-18-0 | as a human metabolite |
| Lipids and lipid-like molecules | level1 | 277.216 | 17.9 | alpha-Linolenic acid | 3.91 | 463-40-1 | Shown to have an antithrombotic effect. It has a role as a micronutrient, a nutraceutical and a mouse metabolite. |
| Lipids and lipid-like molecules | level1 | 277.216 | 17.9 | gamma-Linolenic acid | 3.9 | 506-26-3 | It has a role as a human metabolite, a plant metabolite and a mouse metabolite |
| Lipids and lipid-like molecules | level2 | 518.3214 | 123.1 | LysoPC(18:3(6Z,9Z,12Z)) | 2.34 |  | unknown |
| Lipids and lipid-like molecules | level2 | 375.219 | 22.6 | Resolvin_D2 | 2.69 | 82864-77-5 | unknown |
| Lipids and lipid-like molecules | level1 | 313.237 | 26.2 | Octadecanedioic acid | 3.66 | 871-70-5 | Pharmaceutical Intermediates |
| Lipids and lipid-like molecules | level1 | 520.3371 | 121.8 | LPC(18:2/0:0) | 3.81 | 22252-07-9 | It has a role as a mouse metabolite |
| Lipids and lipid-like molecules | level2 | 667.3284 | 287.1 | 14,16,17,20-Tetrahydroxy-1,26-dioxo-22,26-epoxyergosta-5,24-dien-3-yl .beta.-D-glucopyranoside | 2.18 |  | unknown |
| Lipids and lipid-like molecules | level2 | 427.2274 | 258.8 | Cinnzeylanine | 2.06 | 62203-47-8 | unknown |
| Lipids and lipid-like molecules | level2 | 465.2042 | 208.7 | Beclomethasone 21-propionate | 2.3 | 69224-79-9 | unknown |
| Lipids and lipid-like molecules | level2 | 555.3107 | 188.7 | FUSIDIC ACID | 2.18 | 6990-06-3 | It has a role as a protein synthesis inhibitor |
| Lipids and lipid-like molecules | level2 | 337.1829 | 125.3 | 3-(2-Hydroxyethyl)-4-(hydroxymethyl)hex-5-en-1-yl .beta.-D-glucopyranoside | 2.12 |  | unknown |
| Lipids and lipid-like molecules | level2 | 495.215 | 209.7 | Fluocinonide | 2.13 | 356-12-7 | as a Corticosteroid Hormone Receptor Agonist |
| Lipids and lipid-like molecules | level2 | 528.3113 | 243.6 | LysoPE(22:5(7Z,10Z,13Z,16Z,19Z)/0:0) | 2.21 |  | unknown |
| Lipids and lipid-like molecules | level2 | 526.2398 | 223.9 | Sonolisib | 2.17 | 502632-66-8 | small-molecule wortmannin analogue inhibitor |
| Lipids and lipid-like molecules | level2 | 554.3422 | 141.6 | 3,5,9-Trioxa-4-phosphatetracosan-1-aminium, 7-(acetyloxy)-24-carboxy-4-hydroxy-N,N,N-trimethyl-, inner salt, 4-oxide, (R)- | 2.54 | 129879-41-0 | unknown |
| Lipids and lipid-like molecules | level2 | 309.2055 | 23.9 | FA 18:3+2O | 2.69 |  | unknown |
| Lipids and lipid-like molecules | level2 | 359.2188 | 22.3 | 12-(Acetyloxy)pimara-7,15-dien-18-oic acid | 2.56 |  | unknown |
| Lipids and lipid-like molecules | level1 | 279.2315 | 19.5 | cis-​9,​10-​Epoxystearic acid | 3.84 | 24560-98-3 | unknown |
| Lipids and lipid-like molecules | level2 | 335.2177 | 19.5 | Kaurane-17,18-dioic acid | 2.55 | 58648-80-9 | unknown |
| Lipids and lipid-like molecules | level2 | 483.2705 | 55.3 | 1-Palmitoyl-2-hydroxy-sn-glycero-3-phospho-(1'-rac-glycerol) | 2.65 | 749875-10-3 | unknown |
| Lipids and lipid-like molecules | level2 | 298.1007 | 236.9 | L-Menthone_1,2-glycerol_ketal | 2.73 | 63187-91-7 | unknown |
| Lipids and lipid-like molecules | level2 | 526.2955 | 275.1 | PE(22:6(4Z,7Z,10Z,13Z,16Z,19Z)/0:0) | 2.22 |  | unknown |
| Lipids and lipid-like molecules | level2 | 337.2344 | 18.9 | Prostaglandin F2.alpha. 1,15-lactone | 2.55 | 55314-49-3 | unknown |
| Lipids and lipid-like molecules | level2 | 295.2252 | 53.5 | 13-HOTE | 2.47 |  | unknown |
| Lipids and lipid-like molecules | level2 | 401.2316 | 251.8 | Lucidone_B | 2.64 | 97653-93-5 | Chinese herbal medicinal ingredient |
| Lipids and lipid-like molecules | level2 | 391.2172 | 246.8 | Neoquassin | 2.16 | 76-77-7 | Chinese herbal medicinal ingredient |
| Lipids and lipid-like molecules | level2 | 632.3121 | 293.8 | Mesaconitine | 2.6 |  | unknown |
| Lipids and lipid-like molecules | level2 | 529.2292 | 258.4 | Dutasteride | 2.72 | 164656-23-9 | as an EC 1.3.1.22 [3-oxo-5alpha-steroid 4-dehydrogenase (NADP(+))] inhibitor and an antihyperplasia drug |
| Lipids and lipid-like molecules | level2 | 471.2192 | 200.1 | Benzoic acid, 4-[[(1R,2E,4E,6Z,9Z)-1-[(1S)-4-carboxy-1-hydroxybutyl]-2,4,6,9-pentadecatetraen-1-yl]thio]- | 2.18 | 154978-38-8 | as a leukotriene antagonist |
| Lipids and lipid-like molecules | level2 | 359.2267 | 151.7 | Piperoic_acid | 2.07 | 110979-04-9 | unknown |
| Lipids and lipid-like molecules | level2 | 539.2628 | 284.8 | Lucidenic Acid E2 | 2.25 | 98665-17-9 | Chinese herbal medicinal ingredient |
| Lipids and lipid-like molecules | level2 | 529.2826 | 286 | 17-(Furan-3-yl)-3-hydroxy-4,4,8-trimethyl-16-oxo-14,15-epoxyandrostane-1,7-diyl diacetate | 2.21 | 1273559-94-6 | unknown |
| Lipids and lipid-like molecules | level2 | 371.2266 | 157.2 | 17-Phenyltrinor-13,14-dihydroprostaglandin A2 | 2.11 | 130209-80-2 | unknown |
| Lipids and lipid-like molecules | level2 | 497.2693 | 204 | Leukotriene_D4 | 2.24 | 73836-78-9 | as a human metabolite, a bronchoconstrictor agent and a mouse metabolite |
| Nucleosides, nucleotides, and analogues | level1 | 227.0662 | 42.8 | 2'-Deoxyuridine | 3.85 | 951-78-0 | small-molecule inhibitor |
| Organic acids and derivatives | level1 | 150.0573 | 170.5 | Methionine | 3.87 | 59-51-8 | Pharmaceutical Intermediates |
| Organic acids and derivatives | level2 | 293.1131 | 204.8 | Phe-Glu | 2.51 | 3617-45-6 | It has a role as a metabolite |
| Organic acids and derivatives | level1 | 295.1273 | 205.8 | gamma-Glutamylphenylalanine | 3.88 | 7432-24-8 | It has a role as a human urinary metabolite |
| Organic acids and derivatives | level1 | 166.0851 | 155.4 | Phenylalanine | 3.84 | 63-91-2 | alkaline phosphatase inhibitor |
| Organic acids and derivatives | level1 | 132.101 | 157.2 | Leucine | 3.87 | 61-90-5 | Chinese herbal medicinal ingredient |
| Organic acids and derivatives | level1 | 132.101 | 157.2 | Isoleucine | 3.85 | 73-32-5 | It is important in hemoglobin synthesis and regulation of blood sugar and energy levels. |
| Organic acids and derivatives | level1 | 132.101 | 157.2 | Norleucine | 3.84 | 104809-14-5 | unknown |
| Organic acids and derivatives | level1 | 145.0611 | 201.3 | Ala-Gly | 3.82 | 687-69-4 | unknown |
| Organic acids and derivatives | level1 | 147.0755 | 201.2 | Gly-Ala | 3.87 | 3695-73-6 | It has a role as a metabolite |
| Organic acids and derivatives | level1 | 147.0755 | 201.2 | N-Methyl-L-asparagine | 3.74 | 7175-34-0 | unknown |
| Organic acids and derivatives | level1 | 134.0439 | 234.1 | Aspartate | 3.87 | 56-84-8 | Pharmaceutical Intermediates |
| Organic acids and derivatives | level2 | 328.2209 | 144 | Diprotin B | 2.39 | 90614-49-6 | Antibiotics |
| Organic acids and derivatives | level2 | 264.0865 | 42.2 | 3,4-Dihydroxycinnamic acid (L-alanine methyl ester) amide | 2.66 | 778624-05-8 | unknown |
| Organic acids and derivatives | level2 | 175.1066 | 177.9 | Val-Gly | 2.64 | 686-43-1 | It has a role as a metabolite |
| Organic acids and derivatives | level1 | 203.0663 | 242.4 | Glycyl-glutamate | 3.87 | 7412-78-4 | It has a role as a metabolite |
| Organic acids and derivatives | level1 | 246.1546 | 247.6 | Arg-Ala | 3.83 | 40968-45-4 | It has a role as a metabolite |
| Organic acids and derivatives | level1 | 263.1374 | 142.1 | Pro-Phe | 3.87 | 13589-02-1 | It has a role as a metabolite |
| Organic acids and derivatives | level2 | 189.1221 | 170.6 | Ile-Gly | 2.63 | 868-28-0 | It has a role as a metabolite |
| Organic acids and derivatives | level1 | 118.0504 | 208.5 | Homoserine | 3.86 | 672-15-1 | It has a role as a metabolite |
| Organic acids and derivatives | level1 | 189.1334 | 292 | Homoarginine | 3.86 | 156-86-5 | It has a role as an EC 3.1.3.1 (alkaline phosphatase) inhibitor |
| Organic acids and derivatives | level2 | 232.139 | 256.3 | Gly-Arg | 2.62 | 18635-55-7 | It has a role as a metabolite |
| Organic acids and derivatives | level1 | 187.1078 | 163.8 | Glycylleucine | 3.9 | 869-19-2 | It has a role as a metabolite |
| Organic acids and derivatives | level1 | 180.0657 | 181.1 | Tyrosine | 3.82 | 60-18-4 | It has a role as a metabolite |
| Organic acids and derivatives | level1 | 180.0657 | 181.1 | o-Tyrosine | 3.58 | 2370-61-8 | small-molecule inhibitor |
| Organic acids and derivatives | level2 | 900.4184 | 239.1 | Lyciumin_D | 2.32 | 150415-40-0 | unknown |
| Organic acids and derivatives | level2 | 248.1226 | 191.9 | Thr-Gln | 2.52 | 96337-79-0 | It has a role as a metabolite |
| Organic acids and derivatives | level2 | 457.1121 | 33.2 | Raltitrexed | 2.46 | 112887-68-0 | an antimetabolite used in chemotherapy. an inhibitor of thymidylate synthase |
| Organic acids and derivatives | level1 | 104.0699 | 219.3 | 4-Aminobutyric acid (GABA) | 3.87 | 56-12-2 | It has a role as a signalling molecule, a neurotransmitter. |
| Organic acids and derivatives | level2 | 281.0994 | 154.2 | Methionyl-Methionine | 2.61 |  | unknown |
| Organic acids and derivatives | level1 | 90.0544 | 205.5 | Alanine | 3.85 | 56-41-7 | EC 4.3.1.15 (diaminopropionate ammonia-lyase) inhibitor |
| Organic acids and derivatives | level1 | 90.0544 | 205.5 | Sarcosine | 3.8 | 107-97-1 | It has a role as a glycine transporter 1 inhibitor, a glycine receptor agonist |
| Organic acids and derivatives | level1 | 274.1032 | 236.7 | gamma-Glutamylglutamine | 3.86 | 10148-81-9 | It has a role as a human metabolite |
| Organic acids and derivatives | level1 | 130.0866 | 161.5 | 3-Amino-4-methylpentanoic acid | 3.86 | 5699-54-7 | It has a role as a human metabolite |
| Organic acids and derivatives | level1 | 120.0647 | 207.5 | Threonine | 3.87 | 72-19-5 | Pharmaceutical Intermediates |
| Organic acids and derivatives | level2 | 116.0711 | 178.8 | 3-Aminopentanoic acid | 2.66 | 18664-78-3 | unknown |
| Organic acids and derivatives | level2 | 644.322 | 225.2 | S-(PGA1)-glutathione | 2.4 |  | unknown |
| Organic acids and derivatives | level1 | 132.065 | 205 | 4-Hydroxyproline | 3.92 | 51-35-4 | It has a role as a metabolite |
| Organic acids and derivatives | level2 | 329.1913 | 268.9 | Gly-Pro-Arg | 2.63 | 47295-77-2 | unknown |
| Organic acids and derivatives | level2 | 246.1084 | 192.7 | Gln-Thr | 2.55 | 74408-69-8 | unknown |
| Organic acids and derivatives | level2 | 229.1532 | 147.5 | Ile-Pro | 2.64 | 37462-92-3 | It has a role as a metabolite |
| Organic acids and derivatives | level1 | 175.1176 | 295.9 | Arginine | 3.75 | 74-79-3 | It has a role as a nutraceutical, a biomarker |
| Organic acids and derivatives | level2 | 272.1701 | 264.3 | Pro-Arg | 2.61 | 2418-74-8 | It has a role as a metabolite |
| Organic acids and derivatives | level1 | 133.0135 | 233.2 | Malic acid | 3.88 | 6915-15-7 | It has a role as a food acidity regulator and a fundamental metabolite |
| Organic acids and derivatives | level2 | 193.0222 | 35.2 | [3-(Trifluoromethyl)-1H-pyrazol-1-yl]acetic acid | 2.35 | 926241-24-9 | unknown |
| Organic acids and derivatives | level2 | 246.1433 | 179.9 | Ile-Asn | 2.58 | 59652-59-4 | It has a role as a metabolite |
| Organic acids and derivatives | level1 | 244.129 | 179.8 | Gly-Gly-Leu | 3.84 | 14857-82-0 | It has a role as a metabolite |
| Organic acids and derivatives | level1 | 116.0698 | 183.2 | Proline | 3.87 | 147-85-3 | as a micronutrient, a nutraceutical |
| Organic acids and derivatives | level2 | 255.1438 | 188.9 | His-Val | 2.56 | 76019-15-3 | It has a role as a metabolite |
| Organic acids and derivatives | level1 | 173.0923 | 185.3 | Gly-Val | 3.83 | 1963-21-9 | It has a role as a human metabolite |
| Organic acids and derivatives | level1 | 173.0923 | 185.3 | N5-acetyl-l-ornithine | 3.68 | 2185-16-2 | unknown |
| Organic acids and derivatives | level2 | 172.0593 | 182.8 | 2-Amino-5,5,5-trifluoropentanoic acid | 2.61 | 2365-80-2 | unknown |
| Organic acids and derivatives | level2 | 274.1857 | 224.3 | Arg-Val | 2.58 | 2896-20-0 | It has a role as a metabolite |
| Organic acids and derivatives | level2 | 269.1592 | 180.1 | His-Ile | 2.64 | 129050-48-2 | unknown |
| Organic acids and derivatives | level2 | 497.2804 | 269.1 | APGPR_Enterostatin | 2.38 | 117830-79-2 | can reduce fat intake |
| Organic acids and derivatives | level1 | 156.0758 | 239.4 | Histidine | 3.7 | 71-00-1 | Pharmaceutical Intermediates |
| Organic acids and derivatives | level1 | 213.097 | 221.4 | Gly-His | 3.82 | 2489-13-6 | It has a role as a metabolite |
| Organic acids and derivatives | level1 | 163.1067 | 293.5 | 5-Hydroxylysine | 3.86 | 1190-94-9 | It has a role as a human metabolite |
| Organic acids and derivatives | level1 | 147.1119 | 299.9 | Lysine | 3.81 | 56-87-1 | unknown |
| Organic acids and derivatives | level2 | 182.08 | 188.7 | Amino(4-methoxyphenyl)acetic acid | 2.36 | 19789-59-4 | Pharmaceutical Intermediates |
| Organic acids and derivatives | level2 | 213.0162 | 44.4 | 2-Deoxyribose 5-phosphate | 2.3 | 7685-50-9 | It has a role as a metabolite |
| Organic acids and derivatives | level2 | 188.1381 | 59 | N6-(1-Iminoethyl)-L-lysine | 2.18 | 53774-63-3 | Inhibitors of inducible nitric oxide synthase |
| Organic acids and derivatives | level2 | 525.3097 | 178.9 | VPGPR_Enterostatin | 2.34 | 144964-56-7 | unknown |
| Organic acids and derivatives | level2 | 276.1537 | 174 | Lys-Glu | 2.54 | 45234-02-4 | flavouring agent |
| Organic acids and derivatives | level1 | 88.0399 | 204.9 | beta-Alanine | 3.9 | 107-95-9 | It has a role as an inhibitor and an agonist |
| Organic oxygen compounds | level2 | 121.064 | 136 | Acetophenone | 2.63 | 98-86-2 | Used as a flavoring, solvent, and polymerization catalyst |
| Organic oxygen compounds | level1 | 179.0552 | 174.8 | Galactose | 3.83 | 10257-28-0 | used in trials studying the treatment and diagnosis of Hepatitis C, Hepatic Cancer, Wilsons Disease, Diabetic Macular Oedema, and Focal Segmental Glomerulosclerosis |
| Organic oxygen compounds | level2 | 245.0935 | 49 | Ethanone, 1-(3,4-dihydroxyphenyl)-2-(2-ethyl-1H-imidazol-1-yl)- | 2.72 | 1135683-24-7 | unknown |
| Organic oxygen compounds | level2 | 367.1033 | 167.9 | 3-O-Feruloylquinic acid | 2.38 | 62929-69-5 | It has a role as a plant metabolite |
| Organic oxygen compounds | level2 | 164.0708 | 156.2 | 1-(4-Hydroxyphenyl)-2-methylaminoethanone | 2.66 | 21213-89-8 | unknown |
| Organic oxygen compounds | level2 | 367.1033 | 145.5 | Cnidioside A | 2.35 | 141896-53-9 | unknown |
| Organic oxygen compounds | level2 | 593.2705 | 153.4 | Curine | 2.56 | 436-05-5 | Anti-inflammatory and analgesic |
| Organic oxygen compounds | level2 | 308.0972 | 175 | N-Acetylneuraminic acid | 2.31 | 131-48-6 | In neural transmission, white blood cell vascular exudation, viral or bacterial infections play a biological role |
| Organic oxygen compounds | level2 | 226.0811 | 227.7 | Cyclocytidine | 2.66 | 31698-14-3 | It has a role as a prodrug, an antimetabolite and an antineoplastic agent |
| Organic oxygen compounds | level2 | 499.296 | 232 | 5,6,16-Trihydroxygrayanotox-10-en-3-yl hexopyranoside | 2.46 |  | unknown |
| Organic oxygen compounds | level2 | 590.2917 | 280.3 | Norbuprenorphine glucuronide | 2.62 | 469887-29-4 | unknown |
| Organic oxygen compounds | level2 | 663.2889 | 235.3 | ACARBOSE | 2.14 |  | unknown |
| Organic oxygen compounds | level2 | 570.2968 | 276.1 | isepamicin | 2.44 | 58152-03-7 | exerts a long postantibiotic effect |
| Organic oxygen compounds | level2 | 513.269 | 289 | Andrographiside | 2.25 | 82209-76-5 | unknown |
| Organoheterocyclic compounds | level2 | 222.9869 | 200.7 | 3-Bromoquinolin-4-amine | 2.69 | 36825-36-2 | unknown |
| Organoheterocyclic compounds | level2 | 188.0694 | 157.6 | Atrazine desethyl | 2.71 | 6190-65-4 | cause developmental toxicity and female reproductive toxicity |
| Organoheterocyclic compounds | level2 | 542.3191 | 121.8 | 1H-Indazole-4-carboxamide, N-[(1,2-dihydro-6-methyl-2-oxo-4-propyl-3-pyridinyl)methyl]-1-(1-methylethyl)-6-[2-(4-methyl-1-piperazinyl)-4-pyridinyl]- | 2.13 | 1346704-33-3 | cause developmental toxicity and female reproductive toxicity |
| Organoheterocyclic compounds | level1 | 111.0195 | 35.6 | Uracil | 3.91 | 66-22-8 | Pharmaceutical Intermediates |
| Organoheterocyclic compounds | level1 | 151.0254 | 116.5 | Xanthine | 3.84 | 69-89-6 | It has a role as a Saccharomyces cerevisiae metabolite. |
| Organoheterocyclic compounds | level1 | 151.0254 | 116.5 | Oxypurinol | 3.67 | 2465-59-0 | It has a role as an xanthine oxidase inhibitor and a drug metabolite. |
| Organoheterocyclic compounds | level2 | 415.1377 | 40.1 | [(6R,7R)-7-Hydroxy-7-methyl-8-oxo-3-[(E)-prop-1-enyl]-5,6-dihydro-1H-isochromen-6-yl] 3,6-dihydroxy-4-methoxy-2-methylbenzoate | 2.17 |  | unknown |
| Organoheterocyclic compounds | level2 | 248.0752 | 155.1 | Ethyl 2-amino-4-phenylthiophene-3-carboxylate | 2.65 | 4815-36-5 | unknown |
| Organoheterocyclic compounds | level1 | 161.1064 | 119.2 | Tryptamine | 3.7 | 61-54-1 | small-molecule inhibitor |
| Organoheterocyclic compounds | level2 | 589.2953 | 284.4 | 7-Hydroxy-10-(2-hydroxypropan-2-yl)-2,2a,4,7b,13a-pentamethyl-6-oxo-2a,3,4,6,7,7a,7b,8,9,10,11a,12,13,13a-tetradecahydro-2H-oxeto[2',3':1,5]cyclopenta[1,2-h]pyrano[3,2-a]xanthene-3,8-diyl diacetate | 2.31 |  | unknown |
| Organoheterocyclic compounds | level2 | 424.1572 | 202.5 | 4H-[1,2,4]Triazolo[4,3-a][1,4]benzodiazepine-4-acetamide, 6-(4-chlorophenyl)-N-ethyl-8-methoxy-1-methyl-, (4S)- | 2.53 | 1260907-17-2 | as a bromodomain-containing protein 4 inhibitor, an antineoplastic agent and an apoptosis inducer |
| Organoheterocyclic compounds | level2 | 148.0347 | 41.7 | 4-(Trifluoromethyl)pyridine | 2.5 | 3796-24-5 | unknown |
| Organoheterocyclic compounds | level2 | 470.233 | 279.7 | Nefazodone | 2.65 | 83366-66-9 | It has a role as an antidepressant, a serotonergic antagonist, a serotonin uptake inhibitor, an alpha-adrenergic antagonist and an analgesic. |
| Organoheterocyclic compounds | level2 | 391.0754 | 200.6 | 5H-Thieno[2,3-c]pyran-3-carboxylic acid, 2-[[(benzoylamino)thioxomethyl]amino]-4,7-dihydro-5,5-dimethyl- | 2.14 | 314042-01-8 | can competitively inhibit RAS-related proteins in Brain 7 (Rab7) |
| Organoheterocyclic compounds | level2 | 333.0829 | 203.8 | 2-Hydroxyethylflurazepam | 2.6 | 20971-53-3 | unknown |
| Organoheterocyclic compounds | level2 | 219.0764 | 32.5 | (S)-5-(4-Hydroxyphenyl)-5-ethylhydantoin | 2.66 | 65567-35-3 | unknown |
| Organoheterocyclic compounds | level1 | 158.0604 | 18.5 | 4-Methylquinolin-2-ol | 3.91 | 607-66-9 | unknown |
| Organoheterocyclic compounds | level2 | 452.1884 | 183.7 | Doxazosin | 2.58 | 74191-85-8 | an antihypertensive agent, an alpha-adrenergic antagonist, an antineoplastic agent, a vasodilator agent and an antihyperplasia drug |
| Organoheterocyclic compounds | level1 | 205.0958 | 157.8 | Tryptophan | 3.83 | 73-22-3 | unknown |
| Organoheterocyclic compounds | level2 | 369.119 | 166.7 | 2-[3-(2-Quinolylmethoxy)anilino]benzoic acid | 2 | 105350-26-3 | unknown |
| Organoheterocyclic compounds | level2 | 393.1745 | 191.9 | Sparfloxacin | 2.2 | 110871-86-8 | antibacterial activity |
| Organoheterocyclic compounds | level2 | 205.0806 | 242.4 | 5,6,7,8-Tetrahydrothieno[2,3-b]quinolin-4-amine | 2.65 | 122914-50-5 | unknown |
| Organoheterocyclic compounds | level2 | 309.1231 | 26.3 | 1H-Pyrrole-3-propanoic acid, 5-[(1,2-dihydro-2-oxo-3H-indol-3-ylidene)methyl]-2,4-dimethyl- | 2.58 | 252916-29-3 | used in trials studying the treatment of Lung Cancer, Breast Cancer, Kidney Cancer, Gastric Cancer, and Prostate Cancer |
| Organoheterocyclic compounds | level2 | 443.2307 | 203.3 | Rhodamine B cation | 2.66 | 64381-98-2 | used as tracer dyes |
| Organoheterocyclic compounds | level2 | 390.2097 | 289 | Alfuzosin | 2.58 | 81403-68-1 | is indicated for benign prostatic hyperplasia |
| Organoheterocyclic compounds | level2 | 448.2193 | 258.3 | Aspergamide B | 2.58 | 863132-99-4 | unknown |
| Organoheterocyclic compounds | level2 | 274.1395 | 174.3 | 5-Heptyl-2,4-dihydro-4-phenyl-3H-1,2,4-triazole-3-thione | 2.65 | 111650-94-3 | unknown |
| Organoheterocyclic compounds | level2 | 443.2224 | 204.2 | LOVASTATIN | 2.16 | 75330-75-5 | an anticholesteremic drug and an antineoplastic agent |
| Organoheterocyclic compounds | level2 | 175.0854 | 57.4 | Indole-3-acetamide | 2.45 | 879-37-8 | unknown |
| Organoheterocyclic compounds | level2 | 198.1225 | 206.7 | 1,3-Dimethyl-6-(propylamino)-2,4(1H,3H)-pyrimidinedione | 2.64 | 5770-45-6 | unknown |
| Organoheterocyclic compounds | level2 | 275.1389 | 26.4 | 5-[4-(Isopentyloxy)phenyl]-5-methyl-2,4-imidazolidinedione | 2.68 | 68524-21-0 | unknown |
| Organoheterocyclic compounds | level2 | 340.1447 | 152.1 | N-(1,3-Diphenyl-1H-pyrazol-5-yl)benzamide | 2.72 | 77746-90-8 | unknown |
| Organoheterocyclic compounds | level2 | 372.222 | 259.2 | 7H-Pyrrolo[3,2-f]quinazoline-1,3-diamine, N3-cyclopropyl-7-[[4-(1-methylethyl)phenyl]methyl]- | 2.58 | 245520-69-8 | as an antibacterial agent, a protease-activated receptor-1 antagonist, a cardioprotective agent and an apoptosis inducer |
| Organoheterocyclic compounds | level1 | 72.0803 | 178.4 | Pyrrolidine | 3.79 | 123-75-1 | unknown |
| Organoheterocyclic compounds | level2 | 205.0629 | 143.2 | 1-(5-Nitro-1H-indol-3-yl)ethan-1-one | 2.57 | 4771-10-2 | unknown |
| Organoheterocyclic compounds | level2 | 374.1666 | 207.6 | 1H-Indazole-3-carboxamide, 1-(5-fluoropentyl)-N-1-naphthalenyl- | 2.6 | 1445581-91-8 | unknown |
| Organoheterocyclic compounds | level1 | 130.0644 | 22.2 | Isoquinoline | 3.86 | 119-65-3 | unknown |
| Organoheterocyclic compounds | level2 | 301.128 | 105.5 | 3-(1H-Indol-4-yl)-N-(3-methoxypropyl)-1,2,4-oxadiazole-5-carboxamide | 2.31 | 1010925-99-1 | unknown |
| Organoheterocyclic compounds | level2 | 315.1645 | 206.3 | Safranine cation | 2.52 | 7006-08-8 | It has a role as a fluorochrome and a histological dye. |
| Organoheterocyclic compounds | level2 | 535.2699 | 289.4 | Pyropheophorbide-a | 2.73 | 24533-72-0 | unknown |
| Organoheterocyclic compounds | level2 | 149.0448 | 41.9 | 1-Methyl-1H-pyrazolo[3,4-d]pyrimidin-4-ol | 2.52 | 5334-56-5 | unknown |
| Organoheterocyclic compounds | level1 | 123.0546 | 28.8 | Nicotinamide | 3.86 | 98-92-0 | an antioxidant, a neuroprotective agent, an anti-inflammatory agent |
| Organoheterocyclic compounds | level2 | 380.1882 | 153.6 | 1,3,5-Trihydroxy-2,4-bis(3-methylbut-2-enyl)-10H-acridin-9-one | 2.15 | 28233-35-4 | unknown |
| Organoheterocyclic compounds | level2 | 156.0646 | 25.7 | 5-Isopropyl-3-isoxazolecarboxylic acid | 2.65 | 89776-74-9 | unknown |
| Organoheterocyclic compounds | level2 | 411.2146 | 281.4 | Risperidone | 2.45 | 106266-06-2 | antipsychotic, a dopaminergic antagonist |
| Organoheterocyclic compounds | level2 | 531.2745 | 195 | 4-(2-Oxo-1,2,3,4-tetrahydroquinolin-6-yl)oxycilostazol | 2.22 | 1796891-27-4 | unknown |
| Organoheterocyclic compounds | level2 | 331.1609 | 266.7 | [4-[(E)-2-(1H-Indazol-3-yl)vinyl]phenyl]piperazinomethanone | 2.52 | 1000669-72-6 | potential antineoplastic activity |
| Organoheterocyclic compounds | level2 | 189.0858 | 203.1 | 6-(2-Pyridinyl)-1,3,5-triazine-2,4-diamine | 2.53 | 25007-79-8 | unknown |
| Organoheterocyclic compounds | level2 | 451.123 | 113.8 | [(3R,3'R,4R,6'S,7R)-4,7-Diacetyloxy-5-hydroxy-6',7-dimethyl-6,8-dioxospiro[4H-isochromene-3,2'-oxane]-3'-yl] acetate | 2.63 | 2117753-48-5 | unknown |
| Organoheterocyclic compounds | level2 | 456.2444 | 184.2 | (3E)-10b-(2-Methylbut-3-en-2-yl)-3-((5-(2-methylbut-3-en-2-yl)-1H-imidazol-4-yl)methylidene)-6,10b,11,11a-tetrahydro-2H-pyrazino[1',2':1,5]pyrrolo[2,3-b]indole-1,4(3H,5aH)-dione | 2.6 | 871982-52-4 | unknown |
| Organoheterocyclic compounds | level2 | 345.1749 | 205 | 10H-Phenoxazine-10-butanamine, 2-chloro-N,N-diethyl- | 2.25 | 201788-90-1 | unknown |
| Phenylpropanoids and polyketides | level2 | 593.1485 | 54.1 | Oroxin B | 2.67 | 114482-86-9 | pharmaceutical intermediate |
| Phenylpropanoids and polyketides | level2 | 533.1278 | 23.3 | 5,7-Dihydroxy-2-(4-hydroxyphenyl)-6,8-bis(3,4,5-trihydroxyoxan-2-yl)chromen-4-one | 2.26 | 1236257-31-0 | unknown |
| Phenylpropanoids and polyketides | level2 | 609.143 | 39.8 | 2-Mercaptobenzothiazole | 2.18 | 26544-34-3 | It has a role as an EC 3.2.1.18 (exo-alpha-sialidase) inhibitor |
| Phenylpropanoids and polyketides | level2 | 475.1218 | 54.6 | Cirsimarin | 2.15 | 13020-19-4 | It has an effective anti-fat effect |
| Phenylpropanoids and polyketides | level2 | 255.0637 | 54.7 | 7,2'-Dihydroxyflavone | 2.66 | 77298-66-9 | unknown |
| Phenylpropanoids and polyketides | level2 | 477.1017 | 39.5 | Ethylparaben | 2.54 | 20486-34-4 | unknown |
| Phenylpropanoids and polyketides | level2 | 461.1067 | 55 | Flavone base + 3O, 1MeO, C-Hex | 2.71 | 6980-25-2 | unknown |
| Phenylpropanoids and polyketides | level1 | 565.1526 | 101 | Apiin | 3.83 | 26544-34-3 | It has a role as an EC 3.2.1.18 (exo-alpha-sialidase) inhibitor |
| Phenylpropanoids and polyketides | level2 | 415.1012 | 54.1 | Puerarin | 2.68 | 3681-99-0 | an autophagy inducer, a cardioprotective agent, an antioxidant, an anti-inflammatory agent, an antipyretic and a ferroptosis inhibitor |
| Phenylpropanoids and polyketides | level2 | 405.1171 | 37.7 | 2,3,4',5-Tetrahydroxystilbene 2-glucoside | 2.67 | 82373-94-2 | It has a role as an antioxidant, a cyclooxygenase 2 inhibitor, an anti-inflammatory agent, a cardioprotective agent, a platelet aggregation inhibitor and an apoptosis inhibitor |
| Phenylpropanoids and polyketides | level2 | 623.156 | 54.1 | 5-Hydroxy-2-(4-hydroxyphenyl)-7-methoxy-4-oxo-4H-chromen-3-yl 2-O-.beta.-D-galactopyranosyl-.beta.-D-glucopyranoside | 2.1 |  | unknown |
| Phenylpropanoids and polyketides | level2 | 435.1263 | 115.8 | Coatline A | 2.6 | 87441-88-1 | unknown |
| Phenylpropanoids and polyketides | level2 | 463.1211 | 39.7 | 4-(3,4-Dihydroxyphenyl)-7-methoxy-2-oxo-2H-chromen-5-yl .beta.-D-glucopyranoside | 2.66 | 116310-58-8 | unknown |
| Phenylpropanoids and polyketides | level2 | 469.1072 | 54.7 | Trifolirhizin | 2.65 | 6807-83-6 | antifungal effect |
| Phenylpropanoids and polyketides | level2 | 279.0371 | 146.1 | (E)-1-[4-4-Methoxyphenyl]-2-(3,5-dichlorophenyl)ethene | 2.53 | 688348-37-0 | unknown |
| Phenylpropanoids and polyketides | level1 | 255.0638 | 24 | 4',5-Dihydroxyflavone | 3.79 | 6665-67-4 | LOX-1/α-glucosidase inhibitor |
| Phenylpropanoids and polyketides | level2 | 305.0655 | 79.9 | (-)-Epigallocatechin | 2.64 | 970-74-1 | It has a role as an antioxidant |
| Phenylpropanoids and polyketides | level1 | 447.1259 | 54.7 | Glycitin | 3.86 | 40246-10-4 | It has a role as a plant metabolite |
| Phenylpropanoids and polyketides | level1 | 253.0494 | 24 | 7,8-Dihydroxyflavone | 3.9 | 38183-03-8 | it has shown efficacy against several diseases of the nervous system, including Alzheimer's, Parkinson's, and Huntington's. |
| Phenylpropanoids and polyketides | level2 | 509.2093 | 192.8 | (7'R,8'R)-4,7'-Epoxy-3'-methoxy-4',5,9,9'-lignanetetrol_9'-glucoside | 2.31 |  | unknown |
| Phenylpropanoids and polyketides | level2 | 265.1255 | 153.8 | Benzenepropanoic acid, 4-[2-(2-methylphenyl)ethynyl]- | 2.55 | 1082058-99-8 | unknown |
| Phenylpropanoids and polyketides | level2 | 641.3328 | 254.6 | N1,N5,N10-Tris-trans-p-coumaroylspermine | 2.41 |  | unknown |
| Phenylpropanoids and polyketides | level1 | 273.0742 | 74.5 | (±)-Naringenin | 3.65 | 67604-48-2\|480-41-1 | unknown |
| Phenylpropanoids and polyketides | level2 | 283.0599 | 23.9 | Wogonin | 2.67 | 632-85-9 | It has a role as a cyclooxygenase 2 inhibitor, an antineoplastic agent, an angiogenesis inhibitor |
| Phenylpropanoids and polyketides | level2 | 285.0742 | 54.8 | 5,6-Dihydroxy-7-methoxyflavone | 2.66 | 29550-13-8 | neuroprotective agents, anti-inflammatory |
| Phenylpropanoids and polyketides | level2 | 431.0963 | 60.7 | Apigenin-7-O-glucoside | 2.75 | 578-74-5 | It has a role as a non-steroidal anti-inflammatory drug, a metabolite and an antibacterial agent. |
| Phenylpropanoids and polyketides | level2 | 449.1066 | 149.2 | Hovetrichoside C | 2.64 | 210050-28-5 | unknown |
| Phenylpropanoids and polyketides | level2 | 547.1437 | 31.6 | 5,7-Dihydroxy-2-phenyl-6-[3,4,5-trihydroxy-6-(hydroxymethyl)oxan-2-yl]-8-(3,4,5-trihydroxyoxan-2-yl)chromen-4-one | 2.27 | 1214688-92-2 | unknown |
| Phenylpropanoids and polyketides | level1 | 489.1366 | 31.7 | 6''-O-Acetylglycitin | 3.84 | 134859-96-4 | unknown |
| Phenylpropanoids and polyketides | level2 | 271.0587 | 110 | 5,7,2'-Trihydroxyflavone | 2.66 | 73046-40-9 | unknown |
| Others | level3.1 | 476.2764 | 125.2 | 1-(linoleoyl)-sn-glycero-3-phosphoethanolamine | 1.83 |  | unknown |
| Others | level2 | 491.1171 | 58.8 | Oenin | 2.67 |  | unknown |
| Others | level3.1 | 317.1453 | 203 | Imidazolone A | 1.83 |  | unknown |
| Others | level3.1 | 519.1121 | 44.1 | Eujambolin | 1.75 |  | unknown |
| Others | level3.2 | 331.1955 | 172.9 | M331T173 | 1.71 |  | unknown |
| Others | level2 | 851.4344 | 259.2 | (2S,3R)-3-((2'R,3S,4b'R,7'S,10a'R)-7'-((2-O-(6-Deoxy-.alpha.-L-mannopyranosyl)-.beta.-D-glucopyranosyl)oxy)-4b',8',8',10a'-tetramethyl-5-oxotetradecahydro-2'H-spiro[furan-3,1'-phenanthren]-2'-yl)-1-((2R)-4-methyl-5-oxo-2,5-dihydrofuran-2-yl)butan-2-yl acetate | 2.28 |  | unknown |
| Others | level3.1 | 481.1788 | 205 | 1-(glutathion-S-yl)-N-hydroxy-omega-(methylsulfanyl)heptan-1-imine | 1.75 |  | unknown |
| Others | level3.1 | 245.092 | 87.8 | (indol-3-yl)acetyl-alanine | 1.64 |  | unknown |
| Others | level3.2 | 458.2582 | 183.9 | M458T184 | 1.8 |  | unknown |
| Others | level2 | 273.1192 | 206.9 | Gln-Gln | 2.63 | 54419-93-1 | It has a role as a Mycoplasma genitalium metabolite |
| Others | level2 | 334.1778 | 253 | 4'-Cyano[1,1'-biphenyl]-4-yl 4-ethylcyclohexanecarboxylate | 2.15 | 67284-56-4 | unknown |
| Others | level3.1 | 242.0787 | 227.2 | gamma-Glutamyl-beta-cyanoalanine | 1.65 |  | unknown |
| Others | level2 | 579.2887 | 151.7 | (4E,10E)-13,21-Dihydroxy-8,14,19-trimethoxy-4,10,12,16-tetramethyl-3,20,22-trioxo-2-azabicyclo[16.3.1]docosa-1(21),4,10,18-tetraen-9-yl carbamate | 2.27 | 1467661-98-8 | unknown |
| Others | level3.2 | 343.1957 | 185.4 | M343T185 | 1.82 |  | unknown |
| Others | level2 | 303.1757 | 254.8 | Arg-Gln | 2.62 | 2483-17-2 | It has a role as a metabolite. |
| Others | level2 | 597.2996 | 151.7 | LPI(18:2) | 2.64 |  | unknown |
| Others | level2 | 595.2862 | 152.2 | D-myo-Inositol, 1-[2-hydroxy-3-[(1-oxo-9,12-octadecadienyl)oxy]propyl hydrogen phosphate], [S-(Z,Z)]- | 2.64 | 149056-39-3 | unknown |
| Others | level3.1 | 516.2254 | 162.2 | Alpha-Trisaccharide | 1.83 |  | unknown |
| Others | level2 | 404.2119 | 264.9 | N-[(3s,5s,7s)-Adamantan-1-yl]-1-(4-fluorobenzyl)-1H-indazole-3-carboxamide | 2.2 |  | unknown |

Table S1 The list of antimicrobial from up-regulate Secondary metabolites

| **Figure S1** | **MS2 name** | **MS2 score** | **level** | **mz** | **rt** | **Formula** | **CAS** | **Fold_Change** |
| --- | --- | --- | --- | --- | --- | --- | --- | --- |
| A | Clinafloxacin | 2.27 | level2 | 366.1058 | 212.7 | C17H17ClFN3O3 | 105956-97-6 | 1.337270342 |
| B | Dimoxystrobin | 2.24 | level2 | 327.17 | 286.4 | C19H22N2O3 | 149961-52-4 | 3.991892525 |
| C | Euphorbiasteroid | 2.27 | level2 | 553.2778 | 260.6 | C32H40O8 | 28649-59-4 | 9.137104178 |
| D | Nalidixic Acid | 3.79 | Level1 | 233.0908 | 33.2 | C12H12N2O3 | 389-08-2 | 4.739996659 |
| E | Pipemidic acid | 2.64 |  | 304.1419 | 254.3 | C14H17N5O3 | 51940-44-4 | 1.755255002 |
