# Supplementary figures and images for: The anti-Candida haemulonii activity and bioactive metabolites of Streptomyces anandii NC-SA6
Source: Front Cell Infect Microbiol. 2026 Jun 30;16:1851966. doi: 10.3389/fcimb.2026.1851966 (PMC13364919; doi:10.3389/fcimb.2026.1851966)

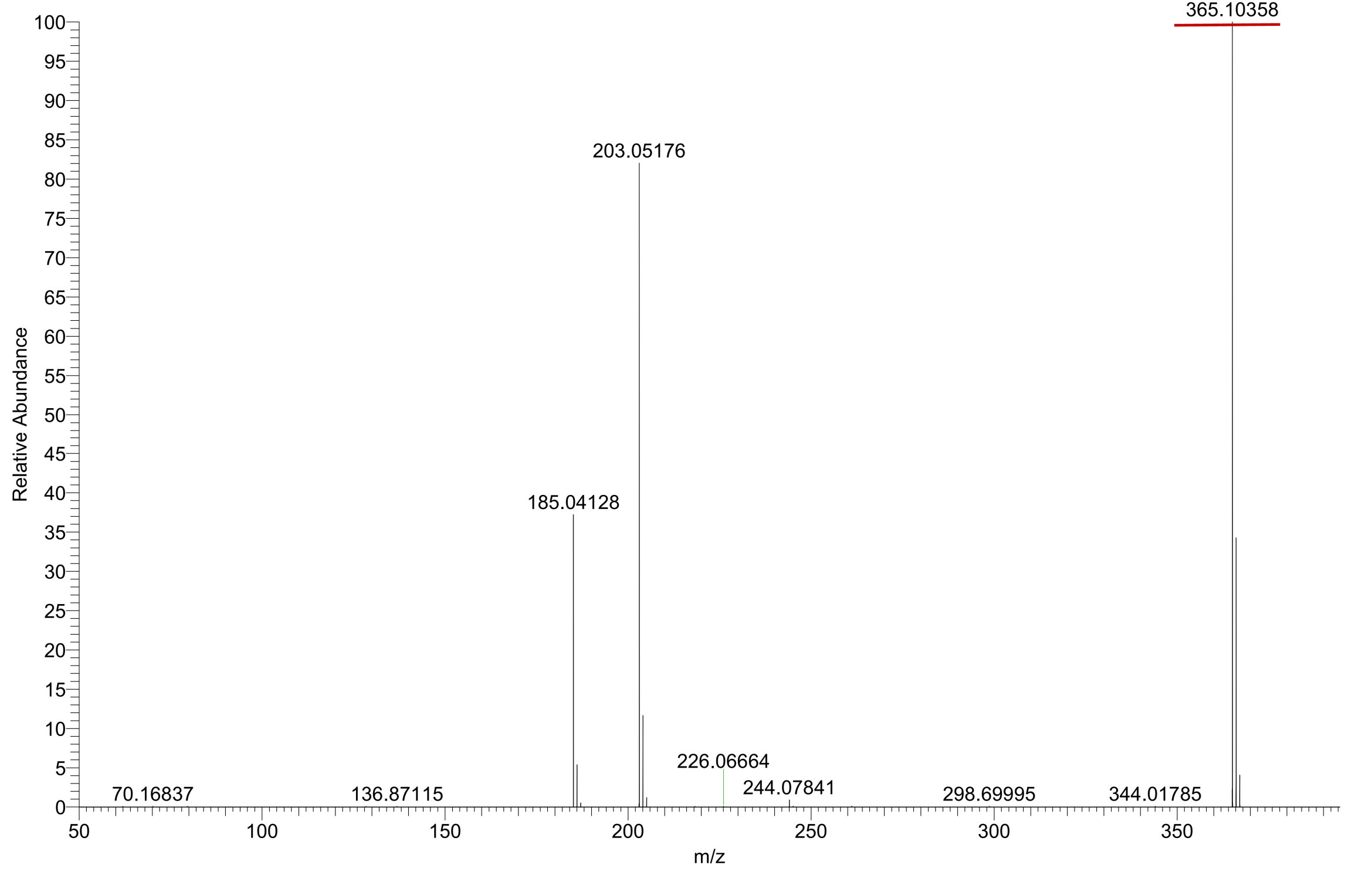

Supplement: Supplementary Figure 1 — Mass spectrogram of antimicrobial from up-regulate Secondary metabolites. [file Image1.png]

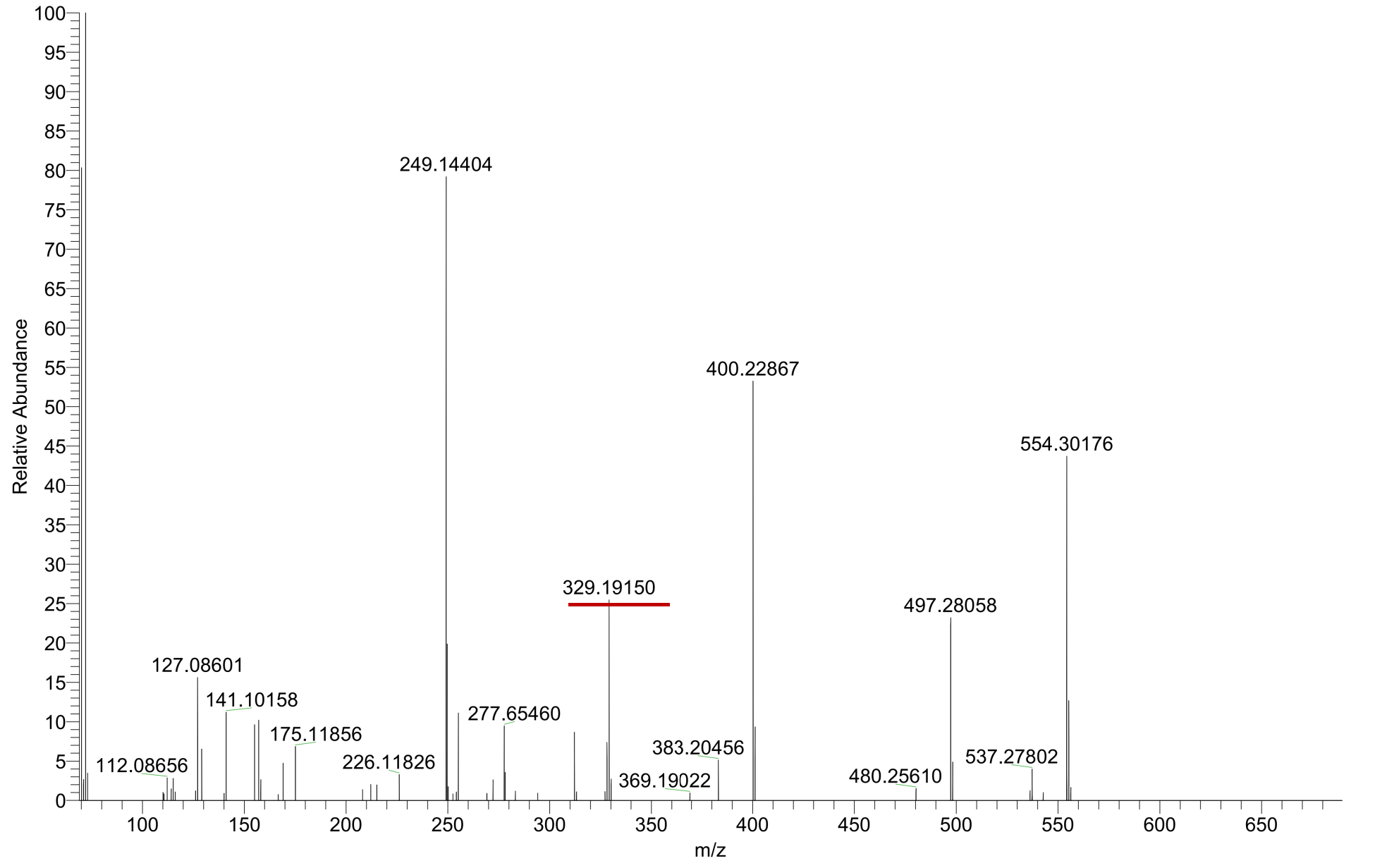

Supplement: Supplementary file 3 [file Image2.png]

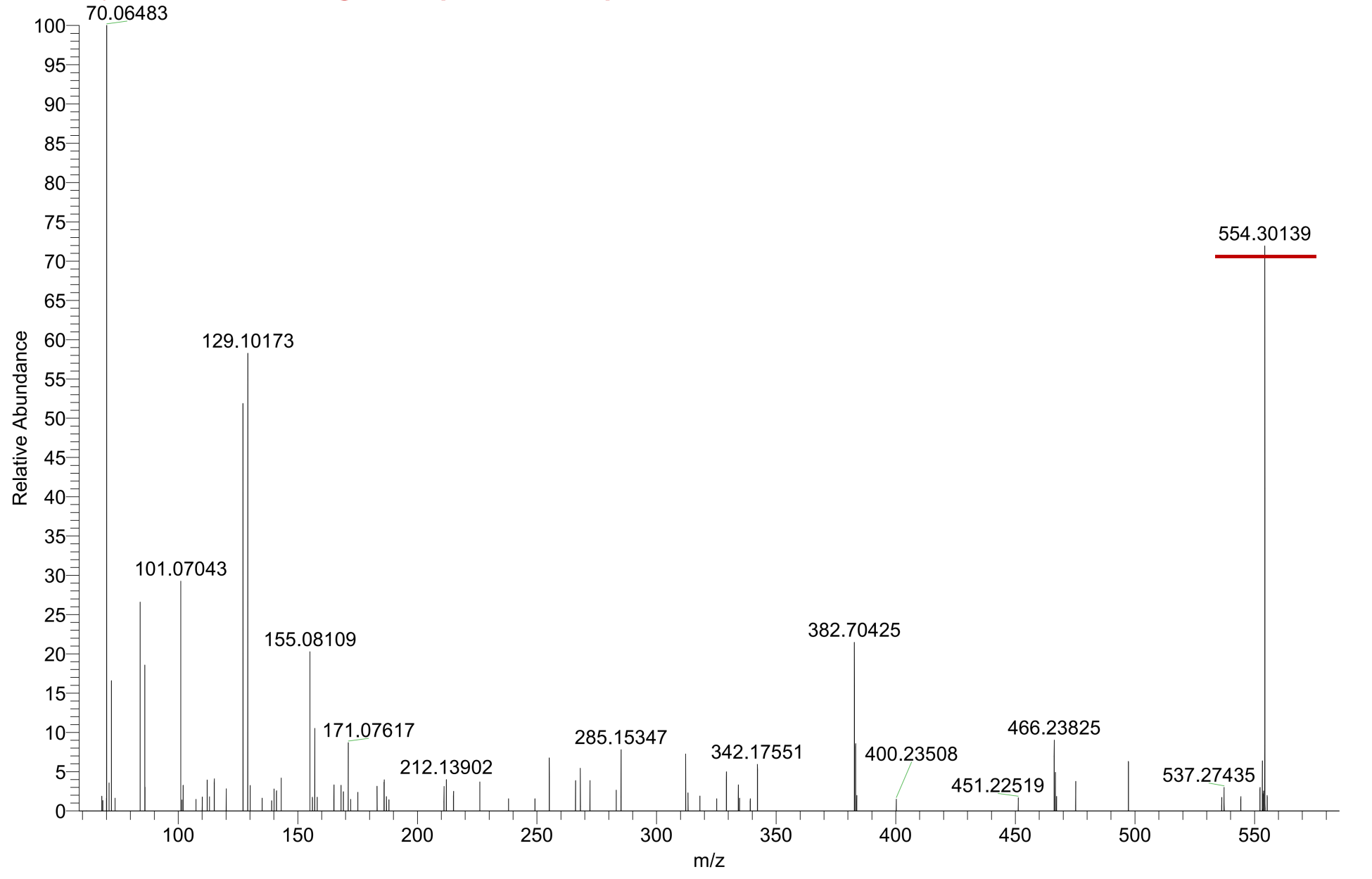

Supplement: Supplementary file 4 [file Image3.png]

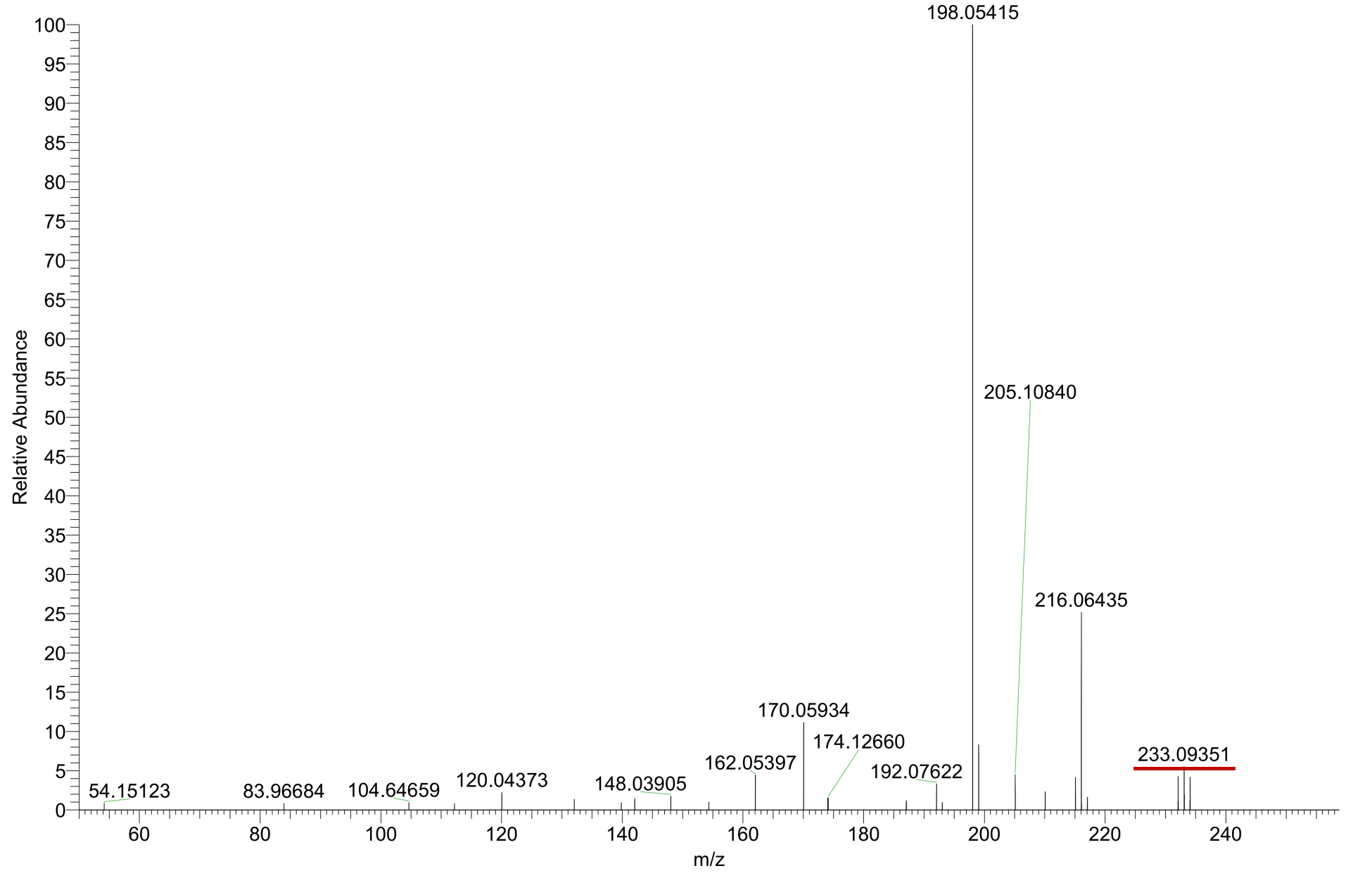

Supplement: Supplementary file 5 [file Image4.png]

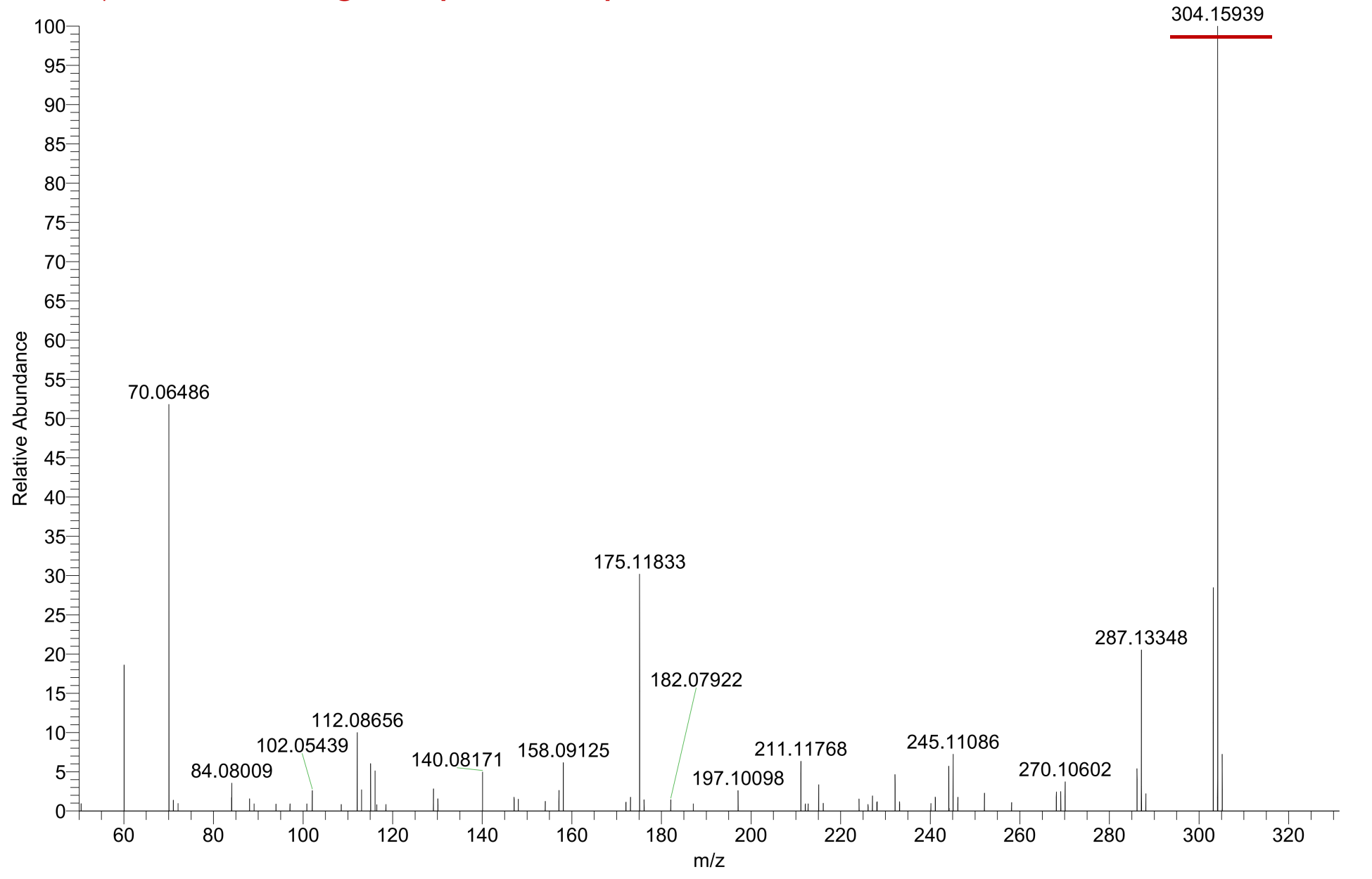

Supplement: Supplementary file 6 [file Image5.png]
